# Rhenium Chalcogenide Clusters Containing *para*-Substituted Phenylacetylide Ligands: Synthesis, Characterization, and Investigation of Substituent Effects on Spectroscopic and Electrochemical Properties

**DOI:** 10.1021/acs.organomet.5c00245

**Published:** 2025-09-25

**Authors:** Katherine L. Helmink, Cory A. Hicks, B. Sage Lauper-Cook, Steven J. Peters, Christopher J. A. Daley, Yann Molard, Lisa F. Szczepura

**Affiliations:** † Department of Chemistry, 6049 Illinois State University, Normal, Illinois 61790-4160, United States; ‡ Department of Chemistry and Biochemistry, 7119 University of San Diego, 5998 Alcalá Park, San Diego, California 92110, United States; § Université de Rennes, CNRS, ISCR − UMR 6226, ScanMAT − 2025, UAR 2025, Rennes F-35000, France

## Abstract

Four new organometallic [Re_6_Se_8_]^2+^-based cluster complexes containing *para*-substituted phenylacetylide ligands, [Re_6_Se_8_(PEt_3_)_5_(C≡C–C_6_H_4_–X)]­(SbF_6_) where X = NO_2_, CO_2_Me, CH_3_, and OMe, have been synthesized and fully characterized using ^1^H, ^13^C­{^1^H} and ^31^P­{^1^H} NMR, IR, and UV–vis spectroscopy, HRMS, and elemental analysis. Three of these cluster complexes, [Re_6_Se_8_(PEt_3_)_5_(C≡C–C_6_H_4_–NO_2_)]^+^, [Re_6_Se_8_(PEt_3_)_5_(C≡C–C_6_H_4_–CO_2_Me)]^+^, and [Re_6_Se_8_(PEt_3_)_5_(C≡C–C_6_H_4_–CH_3_)]^+^ were also characterized via single-crystal X-ray diffraction. Electrochemical studies of these newly reported complexes, as well as the unsubstituted phenylacetylide complex, [Re_6_Se_8_(PEt_3_)_5_(C≡C–C_6_H_5_)]­(SbF_6_), reveal a correlation between the Hammett parameter σ_p_ and the [Re_6_Se_8_]^3+/2+^ couple. The reversibility of this oxidative process is also significantly impacted by the nature of the *para*-substituent. The photophysical properties of these phenylacetylide cluster complexes (including *cis*- and *trans*-[Re_6_Se_8_(PEt_3_)_4_(C≡C–C_6_H_5_)_2_]) in solution and in the powder-phase are reported. Our findings confirm *para*-substituent effects on emission; however, the trend is opposite to that found in previous studies involving [Re_6_Se_8_]^2+^ clusters containing *para*-substituted benzonitrile ligands.

## Graphical abstract

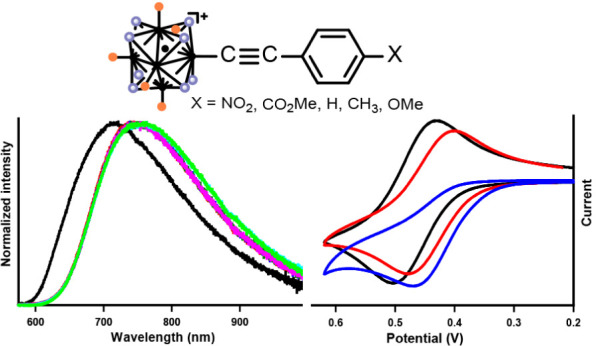

## Introduction

Alkynyl ligands are versatile ligands in terms of their bonding modes and reactivity, and much of the sustained interest in transition metal complexes containing M–C≡C–R moieties has been fueled by their physical properties and potential applications in materials.^1–3^ Numerous reports detail the luminescent properties of metal complexes containing σ-bound alkynyl ligands, as well as the preparation of functional materials containing metal alkynyl or polymetallyne complexes.^4–6^ Folded into these application studies are investigations into how changes to the alkynyl substituent (M–C≡C–R),^7,8^ chain length,^9,10^ or the substituent between alkynyl moieties (M–C≡C–R–C≡C–M)^11,12^ can impact the physical properties or reactivity of these organometallic alkynyl compounds or materials. These studies have led to a deeper understanding of the fundamental properties of these metal-alkynyl moieties.

The seminal reports by Gladysz and coworkers proved that terminal alkynes bonded to transition metal centers can be oxidatively coupled to generate bridging alkynyl species.^9,13,14^ Much of this early work was conducted using cyclopentadienyl rhenium­(I) acetylide complexes. For example, [Cp*Re­(PPh_3_)­(NO)­(C≡C–C≡C)­(NO)­(PPh_3_)­ReCp*] is generated in high yield when [ReCp*­(PPh_3_)­(NO)­(C≡CH)] undergoes homocoupling in the presence of Cu­(OAc)_2_.^13^ Other studies involving low oxidation state rhenium complexes include carbonyl containing rhenium­(I) acetylide complexes such as those studied by Yam and coworkers who investigated the luminescent properties and reactivity of these and related complexes.^15,16^ As outlined in various reviews, a wide range of single transition metal acetylide complexes, as well as small nuclearity acetylide clusters have been investigated. Owing to the tendency of group 11 metals to form atom-precise nanoclusters and the unique bonding interactions of the d^10^ metals (e.g., Cu­(I), Ag­(I), and Au­(I)) with alkynyl ligands which interact with several alkynyl ligands at once (σ-bound anionic ligands can stabilize the nanoparticle, whereas π-interactions have been shown to influence photophysical properties) there has been ongoing interest in alkynyl containing clusters of the coinage metals.^3,17^ More recently, the structures and properties of alkynyl protected nanoclusters (such as Ag_22_Cu_7_) have been examined.^18^ Although there are a few examples of the related [Mo_6_X_8_]^4+^ clusters containing organometallic ligands^19,20^ the literature on rhenium­(III) acetylides and the organometallic chemistry of [Re_6_Se_8_]^2+^-based clusters, in general, is noticeably lacking.

In 2022, we reported the first [Re_6_Se_8_]^2+^-acetylide containing cluster complexes.^21^ This study focused on the preparation of mono- and diacetylide rhenium selenide cluster complexes as well as the reactivity of [Re_6_Se_8_(PEt_3_)_5_(C≡CPh)]^+^. Of significance was our finding that the reaction of [Re_6_Se_8_(PEt_3_)_5_(C≡CPh)]^+^ with electrophilic alkylating reagents (such as Me_2_SO_4_) in MeCN led to the formation of free Me–C≡C–Ph and [Re_6_Se_8_(PEt_3_)_5_(N≡CCH_3_)]^2+^. Notably, alkylation at C_α_, and subsequent loss of alkyne, was unexpected in comparison with most single metal acetylides where alkylation is known to occur at C_β_ leading to the formation of vinylidene species. These findings prompted us to further investigate the physical properties of this [Re_6_Se_8_]^2+^-based phenylacetylide cluster complex, which is the focus of this report.

Lapinte and coworkers utilized various *para*-substituted phenylacetylide ligands to conduct a detailed exploration into the nature of the metal-acetylide bond in monometallic [M–C≡C–C_6_H_4_–X]^*n*^^+^ systems where M = Fe­(II or III) or Ru­(II).^22,23^ Here we apply a similar strategy by varying the *para*-substituent of the phenylacetylide ligand in order to probe the electrochemical (cyclic voltametric) and photophysical properties (luminescence) of [Re_6_Se_8_(PEt_3_)_5_(C≡C–C_6_H_4_–X)]^+^. Specifically, we report the preparation of four different *p*-substituted phenylacetylide cluster complexes. The synthesis, characterization and luminescent properties of these *para*-substituted monophenylacetylide complexes (**1**, **2**, **4**, **5**) are discussed along with that of the unsubstituted phenylacetylide complex^21^ (**3**) and the two bis-phenylacetylide cluster complexes, *cis*-[Re_6_Se_8_(PEt_3_)_4_(C≡CPh)_2_] (**6**) and *trans*-[Re_6_Se_8_(PEt_3_)_4_(C≡CPh)_2_] (**7**). These studies provide insight into the nature of the cluster-acetylide interaction. In addition, our studies are significant in that they expand the fundamental organometallic chemistry of this novel cluster core.
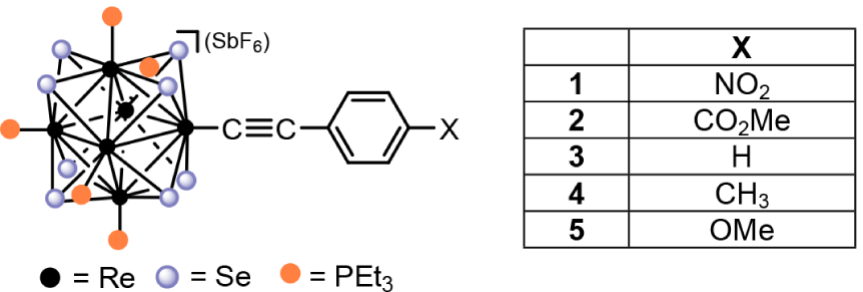


## Results and Discussion

### Synthesis

All of the newly reported complexes (**1**, **2**, **4** and **5**) are air-stable and were prepared by reaction of [Re_6_Se_8_(PEt_3_)_5_I]I with the *para*-substituted phenylacetylene of interest in the presence of triethylamine and a silver­(I) salt. This procedure is similar to that used in the preparation of the previously reported unsubstituted phenylacetylide complex **3**.^21^ As expected, the substituted phenylacetylide complexes have one less aromatic hydrogen in their ^1^H NMR spectra compared to **3** due to substitution at the *para*-position of the phenyl ring. In addition, compounds **2**, **4**, and **5** contain a singlet assigned to the methyl protons on the different substituents (CO_2_Me, CH_3_, and OMe, respectively). The number of resonances observed in the ^13^C {^1^H} NMR spectra (and the chemical shift of these resonances) also supports the proposed formulation of [Re_6_Se_8_(PEt_3_)_5_(C≡C–C_6_H_4_–X)]­(SbF_6_) (X = NO_2_, CO_2_Me, CH_3_, and OMe). The α carbon of the phenylacetylide moiety (−C_α_≡C_β_–C_6_H_4_–X) appears as a doublet (due to long-range coupling with the ^31^P in the phosphine ligand *trans* to the phenylacetylide moiety) between 85 and 105 ppm; again, this is similar to what was observed in the ^13^C {^1^H} NMR spectrum of **3**. Empirically derived Hammett parameters are often useful when investigating the influence of substituents on the properties or reactivity of a series of compounds. We note a strong linear correlation between the chemical shift of C_α_ and the Hammett parameter σ_p_ (δC_α_ (ppm) = 14.98σ_p_ + 90.72; *R* ^2^ = 0.991). In terms of electrostatic effects, electron withdrawing substituents are predicted to lead to increased deshielding (causing resonances to undergo a downfield shift) which is what is observed. For example, C_α_ for **1** (the compound with the most electron withdrawing substituent) appears at 102.8 ppm, whereas C_α_ for **5** (the compound with the most electron donating substituent) appears at 87.35 ppm. As with other 5:1 site-differentiated [Re_6_Se_8_]^2+^ cluster complexes, the ^31^P­{^1^H} NMR data of compounds **1**–**5** show two resonances in ∼4:1 ratio around −30 ppm. The more intense resonance (which appears more upfield) is assigned to the four PEt_3_ ligands *cis*- to the unique phenylacetylide ligand, and the other resonance is assigned to the PEt_3_ ligand *trans*- to the phenylacetylide ligand. The shift observed for the ^31^P­{^1^H} resonances as the *para*-substituent is varied is similar to that observed for the ^13^C resonances (that is, the resonances shift downfield as the substituent become more electron withdrawing).

The ν­(C≡C) stretch is easily identified in the IR spectral data. This resonance appears between 2084 and 2097 cm^–1^, but there is no apparent correlation with the electronic nature of the *para* substituent on the ν­(C≡C) stretch. However, we do observe a gradual increase in intensity of the ν­(C≡C) stretch with **5** showing the least intense stretch and **1** showing the most intense stretch. This trend in υ­(C≡C) becoming more intense as the *p*-substituent of a coordinated phenylacetylide ligand becomes more electron withdrawing was noted previously in a series of *para*-substituted Fe­(II) complexes (i.e., [Fe­(Cp*)­(dppe)­(C≡C–C_6_H_4_–X)], X = NO_2_, H, OMe, etc.).^24^

### Structural Analysis

Single crystals of **1**, **2** and **4** (Figure 1) were grown and analyzed by X-ray diffraction and key crystallographic information is provided in Table S1. The Re–Re distances of [Re_6_Se_8_(PEt_3_)_5_(C≡C–C_6_H_4_–NO_2_)]^+^, [Re_6_Se_8_(PEt_3_)_5_(C≡C–C_6_H_4_–CO_2_Me)]^+^, and [Re_6_Se_8_(PEt_3_)_5_(C≡C–C_6_H_4_–CH_3_)]^+^, range from 2.6350(3)–2.6565(3) Å, 2.6308(13)–2.6560(13) Å, and 2.6334(5)–2.6458(3) Å respectively, and the Re–Se distances range from 2.5132(5)–2.5285(5) Å, 2.518(3)–2.532(3) Å, and 2.5113(9)–2.5277(9) Å respectively. These [Re_6_Se_8_]^2+^ core dimensions fall within the range of those reported for other [Re_6_Se_8_]^2+^ core-containing complexes (Re–Re: 2.571 – 2.674 Å; Re–Se: 2.464–2.574 Å).^25^ The bond lengths of the terminal phosphine ligands (Re–P) of each complex: 2.4699(13)–2.4931(14) Å, 2.460(2)–2.480(2) Å, and 2.477(6)–2.499(7) Å respectively, also fall within the range of reported 24 electron rhenium selenide clusters containing coordinated triethylphosphine ligands (2.454–2.512 Å).^25^

**Figure 1:**
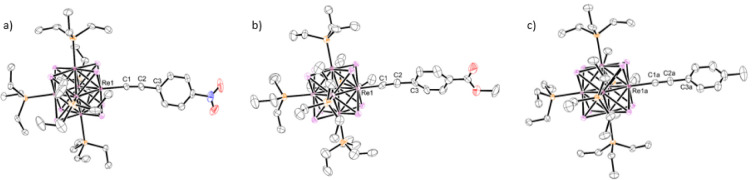
ORTEP diagrams of a) [Re_6_Se_8_(PEt_3_)_5_(C≡C–C_6_H_4_–NO_2_)]^+^, b) [Re_6_Se_8_(PEt_3_)_5_(C≡C–C_6_H_4_–CO_2_Me)]^+^and c) [Re_6_Se_8_(PEt_3_)_5_(C≡C–C_6_H_4_–CH_3_)]^+^. The hexafluoroantimonate (SbF_6_^–^) counterions and H atoms were omitted for clarity and ellipsoids are at 50% probability.

The [Re_6_Se_8_(PEt_3_)_5_(C≡C–C_6_H_4_–NO_2_)]^+^, [Re_6_Se_8_(PEt_3_)_5_(C≡C–C_6_H_4_–CO_2_Me)]^+^, and [Re_6_Se_8_(PEt_3_)_5_(C≡C–C_6_H_4_–CH_3_)]^+^ structures contain Re–C1­(alkyne) bond lengths of 2.090(5) Å, 2.06(3), and 2.110(10) Å and 2.095(10) Å (two independent molecules in unit cell), respectively. These fall within the range of those we reported (Re–C1­(alkyne): 2.088–2.113 Å) for the unsubstituted phenylacetylene clusters, [Re_6_Se_8_(PEt_3_)_5_(C≡C–Ph)]^+^, *cis*-[Re_6_Se_8_(PEt_3_)_4_(C≡C–Ph)_2_], and *trans*-[Re_6_Se_8_(PEt_3_)_4_(C≡C–Ph)_2_].^21^ Similarly, the C1–C2­(alkyne) bond lengths and Re–C–C bond angles of [Re_6_Se_8_(PEt_3_)_5_(C≡C–C_6_H_4_–NO_2_)]^+^ (1.206 Å, 174.0°), [Re_6_Se_8_(PEt_3_)_5_(C≡C–C_6_H_4_–CO_2_Me)]^+^ (1.22 Å, 174°) and [Re_6_Se_8_(PEt_3_)_5_(C≡C–C_6_H_4_–CH_3_)]^+^ (1.184 Å and 1.201 Å, 176.9° and 176.0°) also fall within the ranges reported for the [Re_6_Se_8_(PEt_3_)_6‑x_(C≡C–Ph)_*x*_]^*n*^ (*x* = *n* = 1; *x* = 2, *n* = 0) complexes (C–C alkyne: 1.173 – 1.216 Å, ∠Re–C–C alkyne: 171.0–176.6°).^21^ More broadly, based on a CSD search, the Re^III^–C­(alkyne) and C≡C alkyne bond lengths and Re–C≡C bond angles are also consistent with reported noncluster, mononuclear Re^III^–C≡C complexes (Re^III^–C­(alkyne): 1.973–1.980 Å, C–C alkyne: 1.224–1.232 Å, ∠Re–C–C alkyne: 174.6–176.7°).^26^

### Electrochemical Data

Cyclic voltammetry was used to probe the redox properties of the *para*-substituted complexes (compounds **6** and **7** show limited solubility and were not investigated electrochemically). Starting at the rest potential and scanning in a positive direction all five monophenylacetylide complexes showed a quasi-reversible (**1**–**4**) or irreversible (**5**) oxidative couple (see Table 1 and Figures 2 and S1). Previous reports involving electrochemical studies of related rhenium chalcogenide cluster complexes, such as [Re_6_Se_8_(PEt_3_)_5_I]^+^, [Re_6_Se_8_(PEt_3_)_5_(N≡CCH_3_)]^2+^, and [Re_6_S_8_Cl_4_L_2_]^2–^ (L = PEt_3_), show the presence of an oxidative couple involving the one electron oxidation of the 24-electron cluster core ([Re^III^_6_Q_8_]^2+^ Q = S or Se).^27–29^ Therefore, the oxidative couple observed in these complexes is assigned to the oxidation of the cluster complex (i.e., oxidation of [Re^III^_6_Se_8_]^2+^ to [Re^IV^Re^III^_5_Se_8_]^3+^). Scanning reductively (out to −1.90 V vs FeCp_2_^+/0^), [Re_6_Se_8_(PEt_3_)_5_(C≡C–C_6_H_4_–NO_2_)]­(SbF_6_) (**1**) is the only complex to show an inherent reductive couple (Figure S2). This reversible couple with an *E*_1/2_ = −1.509 V is assigned to the reduction of the nitroaryl group.^22^

**Table 1:** Electrochemical Data for Compounds **1**–**5** in MeCN^a^

	*E*_p,a_ (V)	*E*_p,c_ (V)	*E*_1/2_ (V)	Δ*E*_p_ (mV)	*i*_p,c_/*i*_p,a_
**1** ^b^	0.505	0.432	0.469	73	0.682
**2**	0.493	0.419	0.456	74	0.631
**3**	0.481	0.403	0.442	78	0.543
**4**	0.473	0.398	0.436	75	0.221
**5**	0.469	-	-	n/a	-

a All potentials referenced to the FeCp_2_^+/0^ couple.

b A reductive couple was also observed at *E*_1/2_ = −1.509 V (*i*_p,a_/*i*_p,c_ = 0.971).

**Figure 2:**
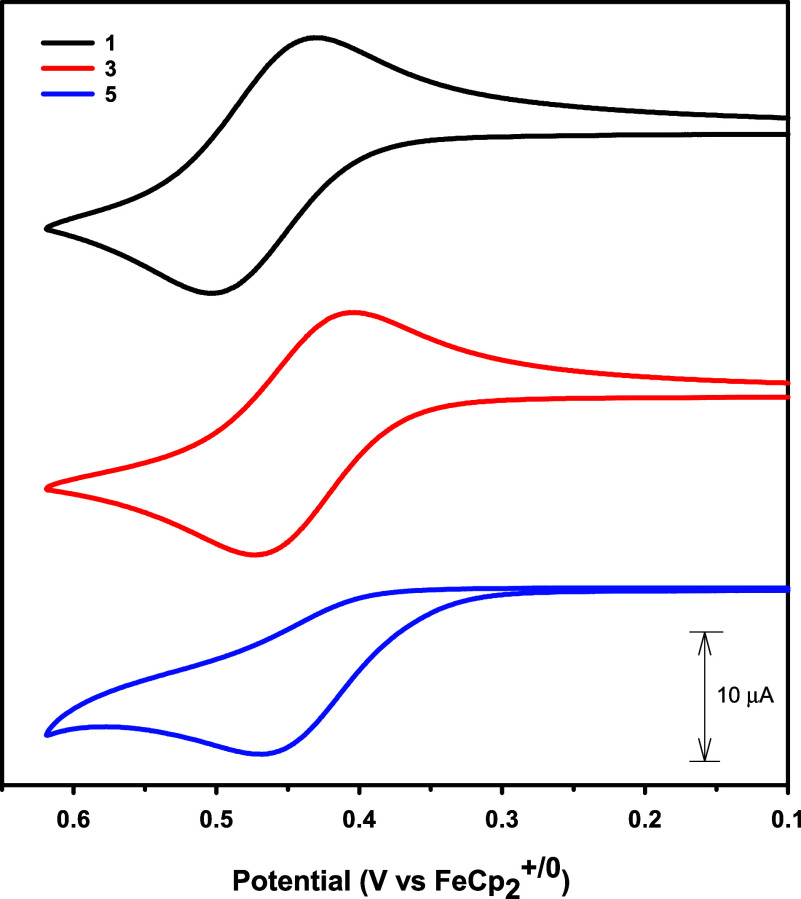
Cyclic voltammetric data for compounds **1** (black), **3** (red), and **5** (blue) (deaerated 0.2 M Bu_4_NBF_4_ MeCN at 100 mV/s).

The *para*-substituent has a measurable impact on the potential of the oxidative couple. Specifically, as the *p*-substituent becomes more electron withdrawing, the redox potential increases, indicating the species is harder to oxidize. A Hammett analysis shows a strong correlation between *E*_1/2_ and σ_p_ of **1**–**4** (Figure 3, *R* ^2^ = 0.999). In order to include compound **5**, which displays an irreversible oxidative couple, we also plotted *E*_p,a_ vs σ_p_ and that shows a similarly strong correlation (Figure S3, *R* ^2^ = 0.997). A similar trend in redox potentials was reported previously for the following series of iron­(II) and ruthenium­(II) complexes, [FeCp*­(dppe)­(C≡C–1,4-C_6_H_4_–X)] and [RuCp*­(dppe)­(C≡C–1,4-C_6_H_4_–X)].^22,23^ However, it is important to note that the impact of the *para*-substituent on the potential of the [Re_6_Se_8_]^2+^ cluster core is less pronounced than that observed for the previously reported single-metal (Fe and Ru) systems. For example, we observe a difference of 0.033 V between the *E*_1/2_ values of **1** to **4**, whereas Lapinte and coworkers report a difference of 0.16 V between the redox potentials of [FeCp*­(dppe)­(C≡C–C_6_H_4_–CH_3_)] and [FeCp*­(dppe)­(C≡C–C_6_H_4_–NO_2_)].^22^ Since the redox processes reported here involve oxidation of the 24e^–^ cluster core, which contains six metal centers, it is not surprising that the impact of a single substituted phenylacetylide ligand is diminished compared to the impact it would have on a single metal center.

**Figure 3:**
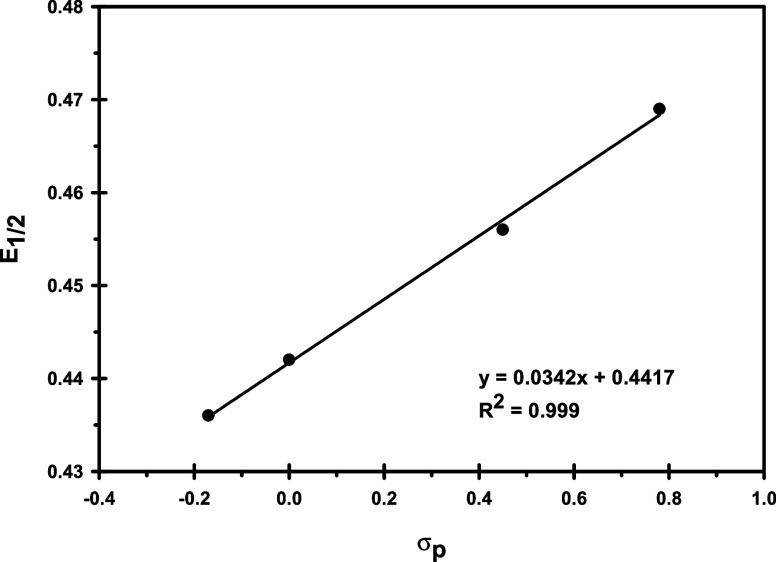
Plot of *E*_1/2_ vs σ_p_ for [Re_6_Se_8_(PEt_3_)_5_(C≡C–C_6_H_4_–X)]­(SbF_6_) (X = NO_2_ (**1**), CO_2_Me (**2**), H (**3**) and CH_3_ (**4**)).

It is worthwhile noting that the impact of the substituents on the electrochemistry of **1** – **5** is different from that observed with a series of *para*-substituted benzonitrile complexes, [Re_6_Se_8_(PEt_3_)_5_(N≡C–C_6_H_4_–X)]­(BF_4_)_2_ (X = NO_2_, COMe, H, OMe and NH_2_).^30^ For this system, it was found that the *para* substituents had little effect on cluster oxidation (i.e., there was a minimal shift in the redox potential with a change in substituent, and no correlation with σ_p_), and notably, the reversibility of the couple was *not influenced* by the different *p*-substituents. Computational studies (*vide infra)* show that the HOMOs for the *para*-substituted benzonitrile complexes are localized on the cluster core, whereas the HOMOs of **1**–**5** are localized on the phenylacetylide ligands justifying why the *para*-substituent in these organometallic complexes have a greater impact on the oxidative couple, than their benzonitrile counterparts.

However, the most notable trend observed in the electrochemical data is how the reversibility of the [Re^IV^Re^III^_5_Se_8_]^3+^/[Re^III^_6_Se_8_]^2+^ couple decreases as the *para*-substituent becomes more electron donating. While increasing the scan rate from 50 to 400 mV/s provides a slight improvement in the peak current ratio, the general trend of decreasing reversibility from compound **1**–**5** holds true at all scan rates tested (50, 100, 200, and 400 mV/s). Of the five cluster complexes studied, **1** displays the most reversible oxidative process in this series with *i*_p,c_/*i*_p,a_ = 0.682. However, this peak current ratio is substantially lower than what is typically seen in cyclic voltammograms of other [Re^III^_6_Q_8_]^2+^ Q = S or Se cluster complexes,^27,31,32^ as well as for previously reported iron­(II) and ruthenium­(II) phenylacetylide containing complexes, where peak current ratios typically range from 0.95–1.0.^22,23^ This indicates that the [Re_6_Se_8_]^2+^-based phenylacetylide clusters are uniquely impacted by the oxidation process. Although the impact of the *para*-substituent on the oxidation potential can be explained, rationalizing why electron donating substituents destabilize the oxidized cluster core is more challenging and is the subject of further studies.

### Spectroscopic Properties

The UV–vis spectral data of the five monophenylacetylide complexes (**1**–**5**) dissolved in acetonitrile are shown in Figure 4. All five compounds show multiple absorptions in the 200–500 nm range. The spectra of **3**–**5** look almost identical with absorptions at ∼225 nm and at ∼275 nm, a shoulder at ∼250 nm and some broad absorptions around 350 and 380 nm. The spectra of **1** and **2** show some similar features as compounds **3**–**5**, with the key difference that the absorption at ∼275 nm appears to have undergone a red shift to longer wavelengths (393 nm for **1**, and 315 nm for **2**). The UV–vis spectra of compounds **6** and **7** were reported previously and show characteristics similar to those seen in the spectra of compounds **3**–**5**.

**Figure 4:**
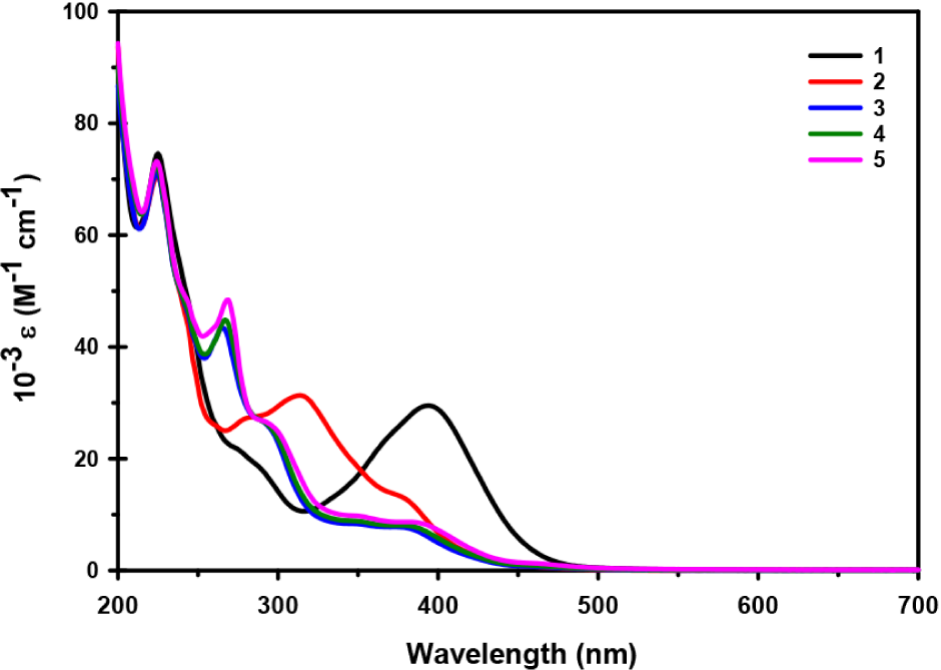
UV–vis spectral data of compounds **1**–**5** in MeCN.

Rhenium chalcogenide cluster complexes are well-known to display red phosphorescence, and the emissive state has been identified as a triplet state which is readily quenched by molecular oxygen. Therefore, we investigated the photophysical properties of complexes **1**–**7** in solution as well as in the solid state. Table 2 provides the emission maxima (λ_em_), excited state lifetimes (τ) and absolute quantum yields (AQY) for all compounds investigated in both phases. The solution phase emission spectra of these cluster complexes are shown in Figure 5 and the solid- state emission spectra are shown in Figure S4. Each of these acetylide complexes contains a broad emission band (λ_em_) centered around 750 nm with a low absolute quantum yield (AQY < 5%). Molecular oxygen was shown to quench the emission process, therefore, solution phase photophysical studies were conducted on deaerated solutions. Quenching by molecular oxygen is indicative of a photoemissive excited triplet state, which is consistent with previous experimental and computational studies on [Re_6_Q_8_]^2+^ cluster complexes.^29,32–34^

**Table 2:** Photophysical Data of Compounds in MeCN and in the Solid State

	CH_3_CN			solid state				
cmpd	λ_em_	τ, μs	AQY	λ_em_	τ_1_, μs (A_1_)	τ_2_, μs (A_2_)	τ_avg_, μs^a^	AQY
**1**	722	11.69^a^,^b^	0.01	740	5.70 (0.33)	9.70 (0.67)	8.8	0.07
**2**	740	5.83	0.04	744	4.17 (0.07)	8.76 (0.93)	8.6	0.07
**3**	739	4.84	0.03	745	4.16 (0.31)	8.27 (0.69)	7.51	0.07
**4**	750	3.79	0.02	762	4.13 (0.25)	9.00 (0.75)	8.3	0.07
**5**	755	3.10	0.03	752	6.04 (0.25)	9.62 (0.75)	9.0	0.08
**6**	757^c^	4.80^c^	0.04^c^	770	1.47 (0.57)	4.43 (0.43)	3.5	0.04
**7**	759^c^	3.10^c^	0.03^c^	792	2.40 (0.49)	6.90 (0.51)	5.8	0.06

a For multiexponential decays the intensity-weighted τ_avg_ values are reported which were calculated using the equation τ_avg_ = (A_1_τ_1_^2^ + A_2_τ_2_^2^)/(A_1_τ_1_+ A_2_τ_2_).^38^

b The τ_avg_ is reported (calculated from τ_1_ = 2.29 (0.24) μs and τ_2_ = 12.24 (0.76) μs).

c Data collected in CH_2_Cl_2_.

**Figure 5:**
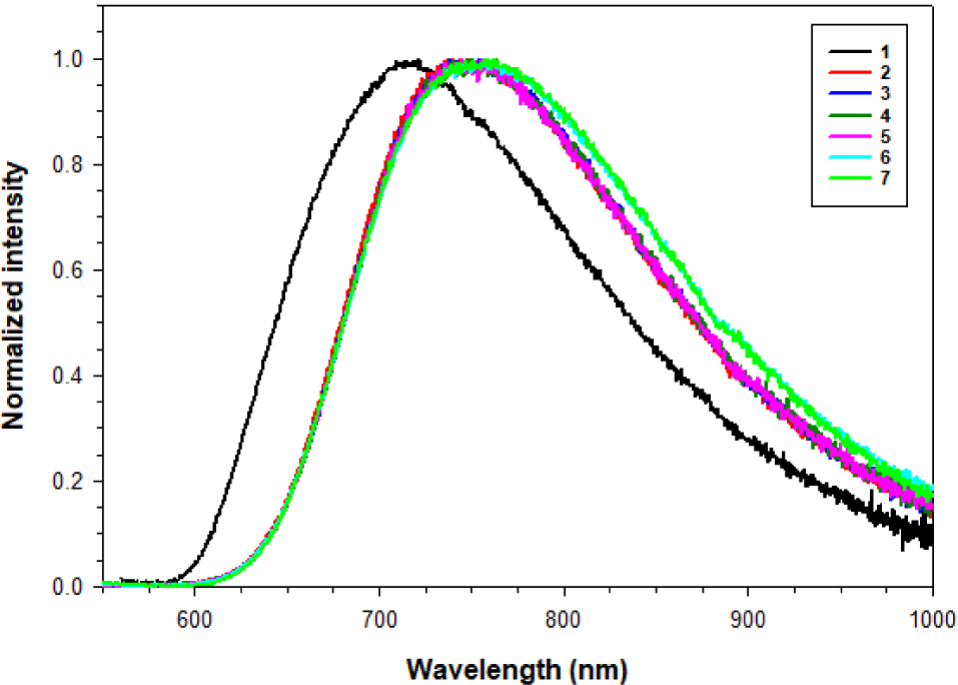
Emission spectra for compounds **1**–**5** in deaerated MeCN, and **6**–**7** in deaerated CH_2_Cl_2_.

In solution and in the solid state, the phosphorescent properties observed for these compounds compare well with previously reported [Re_6_Q_8_]^2+^ clusters which also emit around 750–780 nm with lifetimes (τ) of 1–15 μs and quantum yields that vary, but are generally <10%.^29,31,33,35^ In the solid state, we observed that all complexes emit via a biexponential decay. Previous examples of 24 electron Re_6_ and Mo_6_ clusters displaying monoexponential decay in solution and multiexponential decay in the solid state have been reported in the literature.^28,32,36,37^ This behavior has been attributed to the fact that in solution, solvation prevents interactions between emissive cluster units; however, various phenomena, such as crystal defects or energy traps, which exist in the solid-state can lead to excitation energy migration and subsequent multiexponential decay.^28,36^ The *para*-substituent appears to have minimal impact on the solid-state photophysical properties of compounds **1**–**5**. Therefore, our remaining discussion will focus on the solution phase emission properties.

Within this series of organometallic cluster complexes, **1** stands out in solution as the only compound that has an optimal excitation wavelength (λ_ex_) of 280 nm; **2**–**7** show an optimal λ_ex_ in the visible region (380–420 nm). In addition, **1** emits via a double exponential decay (**2**–**7** emit via a single-exponential decay), has the shortest λ_em_ (722 nm), and the longest average excited state lifetime. Although single-exponential decay is most frequently observed in solution, there are reports of pure [Re_6_Q_8_]^2+^-based clusters displaying multiexponential decay, one possible explanation for this behavior is that the compound undergoes slow decomposition during the irradiation process.^34^ In order to gain a better understanding of factors that impact these properties we calculated the rate constants for radiative (*k*_r_) and nonradiative (*k*_nr_) decay using the equations *k*_r_ = AQY/τ and *k*_nr_ = (1 – AQY)/τ (Table 3). Notably, a linear correlation exists between ln *k*_nr_ and *E*_em_ for compounds **1**–**7** (Figure S5, *R* ^2^ = 0.825) supporting adherence to the energy gap law. Some of the early reports involving the phosphorescence of rhenium selenide and sulfide clusters also noted linear relationships between ln *k*_nr_ and *E*_em_.^33,39^

**Table 3:** Radiative (*k*_r_) and Nonradiative (*k*_nr_) Rate Constants for **1**–**5** in Deaerated MeCN

	*k*_r_ (× 10^3^ s^–1^)	*k*_nr_ (× 10^5^ s^–1^)
**1**	0.855	0.847
**2**	6.86	1.65
**3**	6.20	2.00
**4**	5.28	2.59
**5**	9.68	3.13

The impact of the substituents on the luminescent properties of the *para*-substituted phenylacetylide containing cluster complexes (**1**–**5**), was investigated through the use of Hammett correlations. Examination of our complexes reveals the strongest Hammett correlation between *k*_nr_ and σ_p_ (*k*_nr_ = −1.9 × 10^5^σ_p_ + 2.3 × 10^5^; Figure S6, *R* ^2^ = 0.928). Excited state lifetimes are significantly impacted by nonradiative pathways, since these pathways provide alternative ways to dissipate energy. We also observed a strong correlation between τ and σ_p_ (Figure S7, *R* ^2^ = 0.860) which is expected as slower rates of nonradiative decay are expected to lead to longer excited state lifetimes. The *negative* slope in the correlation between *k*_nr_ and σ_p_ reveals that increasing the electron withdrawing character of the *para* substituent *decreases* the rate of nonradiative decay, thereby, increasing τ. Therefore, it is not surprising that the compound with the most electron *withdrawing* substituent, **1** (X = NO_2_), has the smallest *k*_nr_ and the longest τ_avg_ (11.69 μs). These results provide further evidence that the terminal ligands coordinated to rhenium chalcogenide cluster cores can impact the emission properties of these complexes.^28,29,32,40^

Considering the previously reported series of *para*-substituted benzonitrile complexes, [Re_6_Se_8_(PEt_3_)_5_(N≡C–C_6_H_4_–X)]­(BF_4_)_2_ (X = NO_2_, COMe, H, OMe and NH_2_)^30^ a linear correlation was also observed between τ and σ_p_ (*R* ^2^ = 0.873) and a weaker, but discernible correlation between *k*_nr_ and σ_p_ (*k*_nr_ = +1.6 × 10^5^σ_p_ + 1.2 × 10^5^; *R* ^2^= 0.605). A notable difference between these two sets of complexes is reflected in the Hammett correlations, specifically between *k*_nr_ and σ_p_, which are linearly correlated via a *positive* slope. This is the opposite of what was observed for the substituted phenylacetylide complexes (**1**–**5**). Thus, for the benzonitrile complexes, the compound with the most electron *donating* substituent (NH_2_) has the smallest *k*_nr_ and the longest excited state lifetime (14.88 μs). The fact that a substituent is having a different effect depending on the nature of the ligand itself (phenylacetylide vs benzonitrile) indicates a difference in the impact the substituent is having on the HOMO–LUMO gap.

Previously, we performed calculations on the PMe_3_ analog of [Re_6_Se_8_(PEt_3_)_5_(C≡C–C_6_H_5_)]^+^ and reported that the HOMO was primarily ligand-based, whereas the LUMO was solely cluster based.^21^ Calculations on **1**, **2**, **4** and **5** show similar results (Figures S8–S12), i.e., that the HOMO of these *para*-substituted phenylacetylide complexes contain significant ligand character. In contrast, the LUMOs of compounds **1**–**5** do not show any ligand component, therefore, the LUMOs are cluster based. The one exception is the LUMO for **1** which shows some charge density on the substituted phenylacetylide ligand (Figure S8). (This is not surprising due to the strong electron withdrawing *para*-nitro substituent.) Plotting the HOMO–LUMO energy gap vs σ_p_, we see a strong correlation (*R* ^2^ = 0.964) with a positive slope indicating that as the substituent becomes more electron withdrawing, the energy gap increases. These calculations also justify the trends observed in the electrochemical data in that the clusters with more electron withdrawing substituents are harder to oxidize (due to lowering of the HOMO).

Although we anticipated that changing the *para*-substituent on the phenylacetylide ligand from electron donating to electron withdrawing would decrease the molecular orbital energies, we were uncertain what impact this would have on the HOMO to LUMO energy gap. As mentioned above, our calculations reveal that substantial electron density is found to reside on the ligand in the HOMO of compounds **1**–**5**, which greatly augments the HOMO energy by the *para*-substituent. (In the case of **1** (X = NO_2_), the electron withdrawing effect results in the lowest energy HOMO.) This trend results in an increase in the energy of the HOMO–LUMO gap as the electron withdrawing nature of the substituent increases. Based on this and the adherence to the EGL (Energy Gap Law or correlation between ln *k*_nr_ and *E*_em_), the Hammett correlation between τ and σ_p_ is justified. Notably, these findings for complexes **1**–**5** show a complete reversal in the impact that the *p*-substituents have on τ when compared to the *para*-substituted benzonitrile complexes, [Re_6_Se_8_(PEt_3_)_5_(N≡C–C_6_H_4_–X)]­(BF_4_)_2_ (X = NO_2_, COMe, H, OMe and NH_2_).^30^ In this latter set of complexes, the HOMO was reported to be solely cluster based; thus, the HOMO remained relatively constant regardless of the *para*-substituent. However, this was not the case for the LUMO, which showed substantial electron density residing on the benzonitrile ligand, causing the energy of the LUMO to be impacted by the *para*-substituent (i.e., lowered by electron withdrawing substituents).

The fact that the *para*-substituents have a greater impact on the HOMO of the phenylacetylide complexes, whereas the greater impact is with the LUMO of the *p*-substituted benzonitrile complexes, leads to substituents impacting the photophysical properties differently (see Table 4). Specifically, the phenylacetylide complex with the most electron withdrawing substituent, i.e., [Re_6_Se_8_(PEt_3_)_5_(C≡C–C_6_H_4_–NO_2_)]­(SbF_6_) has the largest HOMO–LUMO gap (and the longest excited state lifetime) in this series (**1**–**5**). However, for the benzonitrile series, substituents that are more electron donating have the larger HOMO–LUMO gap, with [Re_6_Se_8_(PEt_3_)_5_(N≡C–C_6_H_4_–NH_2_)]­(BF_4_)_2_ having the largest gap and the longest excited state lifetime. We predict that the change in how these *para*-substituents impact the magnitude of the energy gap is due to the nature of the ligand itself. Since the phenylacetylide ligands are anionic compared to the neutral benzonitrile ligands, the substituent has a greater impact on the nature of the M-L interactions. While it is known that the photophysical and electrochemical properties of [Mo_6_I_8_(RCOO)_6_]^2–^ are impacted when the carboxylate substituent (R) is varied,^41^ to the best of our knowledge this is the first example of such ligand substituents affecting both of these properties on rhenium chalcogenide cluster complexes.

**Table 4:** HOMO and LUMO Energies for the PMe_3_ Analogues of [Re_6_Se_8_(PEt_3_)_5_(C≡C–C_6_H_4_–X)]^+^ and [Re_6_Se_8_(PEt_3_)_5_(N≡C–C_6_H_4_–X)]^2+^ (X = NO_2_, H, OMe)

[Re_6_Se_8_(PMe_3_)_5_(C≡C–C_6_H_4_–X)]^+^	HOMO (eV)	LUMO (eV)	Δ*E* (eV)^a^
X = NO_2_	–7.001	–4.516	2.485
X = H	–6.549	–4.312	2.237
X = OMe	–6.412	–4.317	2.095
[Re_6_Se_8_(PMe_3_)_5_(N≡C–C_6_H_4_–X)]^2+^			
X = NO_2_	–9.428	–7.545	1.883
X = H	–9.307	–6.939	2.368
X = OMe	–9.209	–6.717	2.492

a Δ*E* = *E*_LUMO_ – *E*_HOMO_.

## Conclusions

With this report we expand the number of organometallic Re_6_-based cluster complexes through the preparation of a series of *para*-substituted phenylacetylide cluster complexes, [Re_6_Se_8_(PEt_3_)_5_(C≡C–C_6_H_4_-X)]­(SbF_6_) where X = NO_2_, CO_2_Me, CH_3_, and OMe. The nature of the *para*-substituent was shown to impact the physical properties of these complexes including NMR chemical shifts, electrochemical and emission properties. Computational studies provide insight into the nature of the HOMO and the LUMO, which allowed us to explain the observed trends. Most notably the nature of the *para* substituent was shown to impact the substituted phenylacetylide complexes differently than clusters containing *para*-substituted benzonitrile ligands. These findings emphasize that substituent properties cannot be generalized, i.e., that a given substituent can impact emission differently, depending on the nature of the ligand itself (e.g., benzonitrile vs phenylacetylide).

## Experimental Section

### General Methods and Materials

The rhenium cluster complex starting material, [Re_6_Se_8_(PEt_3_)_5_I]­I, was prepared using modifications of previously published methods.^42,43^ Specifically, crude (Bu_4_N)_4_[Re_6_Se_8_I_6_] was utilized without further purification. In addition, the reaction of (Bu_4_N)_4_[Re_6_Se_8_I_6_] with PEt_3_ in DMF was monitored by ^31^P­{^1^H} NMR spectroscopy and the reaction time (average 24 h) was determined based on optimizing the yield of the 5:1 product, [Re_6_Se_8_(PEt_3_)_5_I]­I. Dry THF was purchased from Sigma-Aldrich and stored and handled in an inert atmosphere glovebox. Dry THF, 1-ethynyl-4-nitrobenzene, methyl 4-ethynylbenzoate, 4-ethynyltoluene, and 4-ethnylanisole were all purchased from Sigma-Aldrich. Triethylamine was dried using a 2% weight per volume amount of 4 Å molecular sieves; all other materials were purchased and used as received. All experiments were conducted under an inert atmosphere, and no special precautions were taken to exclude air or moisture during workup since the compounds are air-stable. The brine solution was a saturated solution of NaCl. The elemental analysis data was collected by the Microanalysis Laboratory at the University of Illinois Urbana–Champaign.

### Instrumentation

All ^1^H, ^31^P {^1^H}, and ^13^C {^1^H} NMR spectra were recorded using a Bruker Avance III 500 MHz NMR spectrometer. The ^31^P­{^1^H} NMR spectral data were externally referenced to an 85% H_3_PO_4_ reference and collected at 202.5 MHz. The ^13^C­{^1^H} NMR spectral data were collected at 125.8 MHz. Mass spectral data were acquired using positive-mode electrospray ionization (ESI+) and a high-resolution time-of-flight mass spectrometer. UV–vis spectra were recorded on a Cary 3500 Multicell UV–vis spectrophotometer. Fourier-transform infrared (FT-IR) ATR were recorded on a Cary 630 FT-IR spectrometer with a zinc selenide crystal. Electrochemical measurements were conducted in an inert atmosphere glovebox using 0.2 M Bu_4_NBF_4_/MeCN. The three-electrode cell utilized Pt working and auxiliary electrodes and an Ag/AgNO_3_ (0.01 M) reference electrode. All potentials are referenced to the FeCp_2_^+^/FeCp_2_ couple, which was measured under identical conditions.

### Photophysical

The absolute quantum yields in the solid state and in solution were measured with a C9920-03 Hamamatsu system. Emission spectra, emission vs excitation map and excitation spectra were recorded with a Horiba Jobin Yvon Duetta spectrophotometer. The accuracy of the emission spectra was doubled checked on an Edinburgh Instrument FLS 1000 spectrometer. Lifetime in solution were measured with a FLS 1000 spectrometer equipped for the excitation, with a Xe Lamp, a μflash lamp and a double monochromator or a HPL 405 nm picosecond laser diode able to work on MCS mode; the emission signal was recorded after a double monochromator on a red NIR extended R13456 Hamamatsu photodetector. Lifetime measurements in the solid state were realized using a picosecond laser diode (Jobin Yvon DeltaDiode, 375 nm) and a Hamamatsu C10910-25 streak camera mounted with a slow single sweep unit. Signals were integrated on the whole emission decay (the full emission decay profiles are provided in the Supporting Information). Fits were calculated using origin software and the goodness of fit judge by the reduced χ^2^ value and residual plot shape. To cross check the validity of measurements with different experimental setups, some experiments were run twice on the FLS1000 and streak camera setup and gave the same lifetime with an accuracy in the 10% range. All solutions were fully deaerated during 20 min by bubbling N_2_.

### X-ray Crystallographic Data Collection and Refinement

Single crystals of [Re_6_Se_8_(PEt_3_)_5_(C≡C–C_6_H_4_–NO_2_)]­(SbF_6_) were obtained via vapor diffusion of Et_2_O into a concentrated solution of **1** in CH_2_Cl_2_, and single crystals of [Re_6_Se_8_(PEt_3_)_5_(C≡C–C_6_H_4_CH_3_)]­(SbF_6_)·2C_7_H_8_·0.375 H_2_O were obtained *via* vapor diffusion of toluene into a concentrated solution of **4** in CH_2_Cl_2_. [Re_6_Se_8_(PEt_3_)_5_(C≡C–C_6_H_4_–CO_2_Me)]­(SbF_6_)·C_7_H_8_·C_3_H_6_O was isolated from the vapor diffusion of toluene into a concentrated solution of **2** in acetone. Suitable crystals were selected, kept at 173 K, and data were collected on a Bruker APEX II CCD or a Venture Photon III diffractometer with monochromated Mo Κα (λ = 0.71073 Å) radiation. The structures were solved using SHELXT-2014/5 ^44^ and refined on Olex2 ^45^ with the XL^46^ refinement package using Least Squares minimization. For [Re_6_Se_8_(PEt_3_)_5_(C≡C–C_6_H_4_–NO_2_)]­(SbF_6_) the SbF_6_¯ counterion and one of the triethylphosphine ligands show disorder that are modeled in a major and minor orientation conformation. For [Re_6_Se_8_(PEt_3_)_5_(C≡C–C_6_H_4_–CH_3_)]­(SbF_6_), disordered, low resolution, solvent toluene and a small amount of unmodellable electron density were observed in the channels between the complexes such that a solvent mask was applied via BYPASS^47^ in Olex2. The solvent mask was calculated as 3 voids with 415 electrons found in a volume of 911 Å^3^ per unit cell for the largest void, 16 electrons found in a volume of 56 Å^3^ per unit cell in the second void, and 0 electrons found in a volume of 68 Å^3^ per unit cell for a final void. This first void is consistent with the presence of 8 toluene and the second void is consistent with 1.5 water per unit cell (4 toluene and 0.75 water per asymmetric unit), which account for 415 electrons. The unit cell contains two molecules of [Re_6_Se_8_(PEt_3_)_5_(C≡C–C_6_H_4_–CH_3_)]­(SbF_6_) where one of the SbF_6_¯ counterions and one of the triethylphosphine ligands on one of the Re_6_Se_8_ cores show disorder that are modeled in a major and minor orientation conformation. For [Re_6_Se_8_(PEt_3_)_5_(C≡C–C_6_H_4_–CO_2_Me)]­(SbF_6_), some residual electron density in the channels between the parent clusters was unresolvable such that a solvent mask was applied via BYPASS^47^ in Olex2. The solvent mask was calculated as 1 void with 328 electrons found in a volume of 912 Å^3^ per unit cell that has been assigned as 4 toluene and 4 acetone (1 each per asymmetric unit), accounting for 328 electrons. The toluene and acetone model is consistent with the crystallization method used to grow the crystals. The crystallographic data and details of the structure refinements for all compounds are summarized in Table S1.

### Computational Studies

The calculations for the PMe_3_ analogs of [Re_6_Se_8_(PEt_3_)_5_(C≡C–C_6_H_4_–X)]^+^ were carried out by using ORCA 5.0.3 code, where the scalar relativistic effects were incorporated by using the zero-order regular approximation (ZORA).^48–53^ The ethyl groups were replaced with methyl substituents on the phosphine ligands to simplify the calculation. An RHF geometry optimization of the cluster cation was performed with a small frozen core using a standard basis set with valence double-ζ plus polarization (ZORA-SVP for H through Se, and SARC-ZORA-SVP for Re)^51^. A single-point DFT calculation was performed on the optimized structure with electron correlation effects, which were treated within the LDA part of the GGA correction (PW91-LDA)^54^ and included local exchange and correlation gradient corrections (BP86)^55^ and with triple ζ plus polarization (ZORA-def2-TZVP for H through Se, and SARC-ZORA-TZVP for Re).^51^ The energies of the HOMO and LUMO and the respective electron charge density maps for each of the clusters were obtained from these DFT calculations.

#### [Re_6_Se_8_(PEt_3_)_5_(C≡C–C_6_H_4_–NO_2_)]­(SbF_6_) (**1**)

1-Ethynyl-4-nitrobenzene (17.1 mg, 0.116 mmol) was diluted in 4 mL THF in a round-bottom flask. Triethylamine (97.1 μL, 0.697 mmol) was added to the flask with another 12 mL of THF. This solution was sparged with N_2_ for 5 min before [Re_6_Se_8_(PEt_3_)_5_I]I (200.3 mg, 0.0772 mmol) and another 4 mL THF were added. Then, AgSbF_6_ (98.0 mg, 0.285 mmol) was dissolved in THF and added to the solution for a total volume of 55 mL THF. The flask was wrapped in aluminum foil, and the solution was heated at reflux for 3 h under N_2_ atmosphere. Next, the solution was filtered through Celite to remove AgI_(s)_ and then reduced to dryness on the rotary evaporator. The resulting oil was dissolved in CH_2_Cl_2_ and washed with a brine solution three times. The organic solution was then dried using MgSO_4_ and reduced to dryness on the rotary evaporator. The product was purified via column chromatography (silica gel, 100% CH_2_Cl_2_). After elution, the desired band was stripped dry, then reprecipitated using a 1:100 CH_2_Cl_2_:Et_2_O, and isolated (117.4 mg, 56% yield). ^1^H NMR (500 MHz, acetone-*d*_*6*_, δ): 1.16 (45H, m, P­(CH_2_CH_3_)_3_), 2.24 (30H, m, P­(CH_2_CH_3_)_3_), 7.29 (2H, dd, −C_6_ H_4_–NO_2_), 8.14 (2H, dd, −C_6_ H_4_–NO_2_). ^31^P­{^1^H} NMR (202.5 MHz, acetone-*d*_6_, ppm): −30.30 (1P), −31.03 (4P). ^13^C­{^1^H} NMR (125 MHz, acetone-*d*_6_, ppm): 9.50 and 9.59 (P­(CH_2_ CH_3_)), 26.72 and 26.83 (PCH_2_CH_3_), 102.85 (d, Re–C≡C–C_6_H_4_–NO_2_), 124.75, 133.45, 134.82, 135.33, 146.55. UV–vis (CH_3_CN) λ_max_, nm (ε): 225 (76,000), 282 (sh), 292 (sh), 394 (30,400). IR-ATR (ZnSe): ν­(C≡C) 2091 cm^–1^, ν­(−C_6_H_4_–NO_2_) 1504 cm^–1^ (asymmetric) and 1329 cm^–1^ (symmetric). MS (ESI­(+)) *m*/*z*: 2486.5 ([Re_6_Se_8_(PEt_3_)_5_(C≡C–C_6_H_4_–NO_2_)]^+^). Anal. Calcd for C_38_H_79_NP_5_SbF_6_Re_6_Se_8_O_2_: C, 16.77; H, 2.93; N, 0.51. Found: C, 16.82; H, 2.74; N, 0.59.

#### [Re_6_Se_8_(PEt_3_)_5_(C≡C–C_6_H_4_–CO_2_Me)]­(SbF_6_) (**2**)

**2** was prepared similarly to **1** using methyl 4-ethynylbenzoate (23.7 mg, 0.148 mmol) and [Re_6_Se_8_(PEt_3_)_5_I]I (249.9 mg, 0.0964 mmol). After the reaction work up, **2** was isolated (128.5 mg, 49%). ^1^H NMR (500 MHz, acetone-*d*_6_, δ): 1.15 (45H, m, P­(CH_2_CH_3_)_3_), 2.24 (30H, m, P­(CH_2_CH_3_)_3_), 3.85 (3H, s, −C_6_H_4_–COOCH_3_), 7.19 (2H, dd, −C_6_ H_4_–COOCH_3_), 7.88 (2H, dd, −C_6_ H_4_-COOCH_3_). ^31^P­{^1^H} NMR (202.5 MHz, acetone-*d*_6_, ppm): −30.46 (1P), −31.26 (4P). ^13^C­{^1^H} NMR (125 MHz, acetone-*d*_6_, ppm): 9.52 and 9.61 (P­(CH_2_ CH_3_)), 26.74 and 26.86 (PCH_2_CH_3_), 52.74 (−C_6_H_4_–COOCH_3_), 97.28 (d, Re-C≡C–C_6_H_4_–COOCH_3_), 128.29, 130.43, 132.86, 133.34, 135.43, 167.50 (−C_6_H_4_-COOCH_3_). UV–vis (CH_3_CN) λ_max_, nm (ε): 224 (69,500), 242 (sh), 280 (28,600), 314 (32,000), 380 (sh). IR-ATR (ZnSe): ν­(C≡C) 2084 cm^–1^, ν­(C=O) 1716 cm^–1^, ν­(C–O) 1278 cm^–1^. MS (ESI­(+)) *m*/*z*: 2499.6 ([Re_6_Se_8_(PEt_3_)_5_(C≡C–C_6_H_4_–COOCH_3_)]^+^). Anal. Calcd. for C_40_H_82_P_5_SbF_6_Re_6_Se_8_O_2_: C, 17.57; H, 3.02. Found: C, 17.86; H, 3.12.

#### [Re_6_Se_8_(PEt_3_)_5_(C≡C–C_6_H_4_-CH_3_)]­(SbF_6_) (**4**)

In a similar fashion to **1**, **4** was prepared using 4-ethynyltoluene (15 μL, 0.118 mmol) and [Re_6_Se_8_(PEt_3_)_5_I]I (199.9 mg, 0.0771 mmol). After the reaction work up, **4** was isolated (141.5 mg, 68%). ^1^H NMR (500 MHz, acetone-*d*_6_, δ): 1.15 (45H, m, P­(CH_2_CH_3_)_3_), 2.23 (30H, m, P­(CH_2_CH_3_)_3_), 2.32 (3H, s, −C_6_H_4_–CH_3_), 7.03 (4H, m, -C_6_ H_4_-CH_3_). ^31^P­{^1^H} NMR (202.5 MHz, acetone-*d*_6_, ppm): −30.57 (1P), −31.62 (4P). ^13^C­{^1^H} NMR (125 MHz, acetone-*d*_6_, ppm): 9.03 and 9.07 (P­(CH_2_ CH_3_)), 21.21 (s, −C_6_H_4_-CH_3_), 26.20 and 26.33 (PCH_2_CH_3_), 88.42 (d, Re–C≡C–C_6_H_4_–CH_3_), 125.45, 129.29, 132.40, 135.66, 135.95. UV–vis (CH_3_CN) λ_max_, nm (ε): 224 (72,700), 243 (sh), 267 (45,000), 297 (sh), 349 (8,900), 384 (7,950). IR-ATR (ZnSe): ν­(C≡C) 2093 cm^–1^. MS (ESI­(+)) *m*/*z*: 2455.6 ([Re_6_Se_8_(PEt_3_)_5_(C≡C–C_6_H_4_–CH_3_)]^+^). Anal. Calcd for C_39_H_82_P_5_SbF_6_Re_6_Se_8_: C, 17.41; H, 3.07. Found: C, 17.53; H, 3.02.

#### [Re_6_Se_8_(PEt_3_)_5_(C≡C–C_6_H_4_–OMe)]­(SbF_6_) (5)

In a similar fashion to **1**, **5** was prepared using 4-ethynylanisole (15.28 mg, 0.116 mmol) and [Re_6_Se_8_(PEt_3_)_5_I]I (200.0 mg, 0.077 mmol). After the reaction work up, **5** was isolated (99.7 mg, 48%). ^1^H NMR (500 MHz, acetone-*d*_*6*_, δ): 1.16 (45H, m, P­(CH_2_CH_3_)_3_), 2.24 (30H, m, P­(CH_2_CH_3_)_3_), 3.77 (3H, s, −C_6_H_4_–OCH_3_), 6.82 (2H, dd, −C_6_ H_4_-OCH_3_), 7.06 (2H, dd, −C_6_ H_4_–OCH_3_). ^31^P­{^1^H} NMR (202.5 MHz, acetone-*d*_*6*_, ppm): −30.59 (1P), −31.68 (4P). ^13^C­{^1^H} NMR (125 MHz, acetone-*d*_*6*_, ppm): 9.51 and 9.59 (P­(CH_2_ CH_3_), 26.71 and 26.85 (PCH_2_CH_3_), 56.15 (s, −C_6_H_4_–OCH_3_), 87.35 (d, Re–C≡C–C_6_H_4_–OCH_3_), 114.78, 121.41, 134.18, 135.94, 159.49. UV–vis (CH_3_CN) λ_max_, nm (ε): 224 (72,700), 244 (sh), 269 (49,000), 297 (sh), 352 (9,610), 389 (8,440). IR-ATR (ZnSe): ν­(C≡C) 2091 cm^–1^, ν­(C–O) 1239 cm^–1^. MS (ESI­(+)) *m*/*z*: 2471.6 ([Re_6_Se_8_(PEt_3_)_5_(C≡C–C_6_H_4_–OCH_3_)]^+^). Anal. Calcd for C_39_H_82_P_5_SbF_6_Re_6_Se_8_O: C, 17.31; H, 3.05. Found: C, 17.41; H, 2.87.

## Supplementary Material



## Notes

Crystallographic details (for **1**, **2**, and **4**) 4, cyclic voltammograms (for **1**, **2**, and **4**), HOMO and LUMO plots for PMe_3_ analogs of **1**–**5**, powder-phase emission spectra, characterization data for **1**, **2**, **4** and **5**) excitation and emission spectra for **1**–**7** and integrated emission decay profiles for **1**–**7** in solution and in the powder phase (PDF)

## References

[1] Zhang J., Xu L., Ho C.-L., Wong W.-Y. (2017). Functional Organometallic Poly­(Arylene
Ethynylene)­s: From Synthesis to Applications. Top. Curr. Chem..

[2] Ren T. (2005). Diruthenium
σ-Alkynyl Compounds: A New Class of Conjugated Organometallics. Organometallics.

[3] Long N. J., Williams C. K. (2003). Metal Alkynyl σ
Complexes: Synthesis and Materials. Angew. Chem.,
Int. Ed..

[4] Miller-Clark L. A., Ren T. (2021). Syntheses and Material Applications of Ru­(II)­(Bisphosphine)_2_ Alkynyls. J. Organomet. Chem..

[5] Yam, V. W.-W. ; Wong, K. M.-C. Luminescent Molecular Rods — Transition-Metal Alkynyl Complexes. Molecular Wires and Electronics; Springer: Berlin, NY, 2005; pp 1–32.10.1007/b13606922179333

[6] Powell C. E., Humphrey M. G. (2004). Nonlinear Optical
Properties of Transition Metal Acetylides
and Their Derivatives. Coord. Chem. Rev..

[7] Thakker P. U., Aru R. G., Sun C., Pennington W. T., Siegfried A. M., Marder E. C., Wagenknecht P. S. (2014). Synthesis
of Trans Bis-Alkynyl Complexes of Co­(III) Supported by a Tetradentate
Macrocyclic Amine: A Spectroscopic, Structural, and Electrochemical
Analysis of π-Interactions and Electronic Communication in the
CCMCC Structural Unit. Inorg. Chim. Acta.

[8] Benito J., Berenguer J. R., Forniés J., Gil B., Gómez J., Lalinde E. (2003). Synthesis, Characterization and Luminescence Properties
of Homoleptic Platinum­(II) Acetylide Complexes. Dalton Trans..

[9] Bartik T., Bartik B., Brady M., Dembinski R., Gladysz J. A. (1996). A Step-Growth Approach to Metal-Capped
One-Dimensional
Carbon Allotropes: Syntheses of C_12_, C_16_, and
C_20_ μ-Polyynediyl Complexes. Angew. Chem., Int. Ed..

[10] Bryce M. R. (2021). A Review
of Functional Linear Carbon Chains (Oligoynes, Polyynes, Cumulenes)
and Their Applications as Molecular Wires in Molecular Electronics
and Optoelectronics. J. Mater. Chem. C.

[11] Costuas K., Rigaut S. (2011). Polynuclear Carbon-Rich
Organometallic Complexes: Clarification
of the Role of the Bridging Ligand in the Redox Properties. Dalton Trans..

[12] Low P. J., Brown N. J. (2010). Electronic Interactions between and through Covalently-Bonded
Polymetallic Complexes. J. Clust. Sci..

[13] Zhou Y., Seyler J. W., Weng W., Arif A. M., Gladysz J. A. (1993). New Families
of Coordinated Carbon: Oxidative Coupling of an Ethynyl Complex to
Isolable and Crystallographically Characterized MC≡CC≡CM
and + M=C = C=C = C=M+ Assemblies. J.
Am. Chem. Soc..

[14] Brady M., Weng W., Gladysz J. A. (1994). New Families
of Coordinated Carbon:
Oxidative Coupling and Cross-Coupling of a Transition Metal Butadiynyl
Complex to Bimetallic M-C≡CC≡CC≡CC≡C-M
and M-C≡CC≡CC≡C-M Adducts. J. Chem. Soc., Chem. Commun..

[15] Heidrich J., Steimann M., Appel M., Beck W., Phillips J. R., Trogler W. C. (1990). Hydrocarbon-Bridged Complexes. 15. Molecular and Electronic
Structures of (.Mu.-Ethynediyl)­Bis­(Pentacarbonylrhenium), (OC)_5_ReC.Tplbond.CRe­(CO)_5_. Organometallics.

[16] Yam V. W.-W. (2001). Luminescent
Carbon-Rich Rhenium­(i) Complexes. Chem. Commun..

[17] Zhang M.-M., Dong X.-Y., Wang Y.-J., Zang S.-Q., Mak T. C. W. (2022). Recent
Progress in Functional Atom-Precise Coinage Metal Clusters Protected
by Alkynyl Ligands. Coord. Chem. Rev..

[18] Deng G., Lee K., Deng H., Malola S., Bootharaju M. S., Häkkinen H., Zheng N., Hyeon T. (2023). Alkynyl-Protected Chiral
Bimetallic Ag_22_Cu_7_ Superatom with Multiple Chirality
Origins. Angew. Chem., Int. Ed..

[19] Saito T., Nishida M., Yamagata T., Yamagata Y., Yamaguchi Y. (1986). Synthesis
of Hexanuclear Molybdenum Cluster Alkyl Complexes Coordinated with
Trialkylphosphines: Crystal Structures of *Trans*-[(Mo_6_Cl_8_)­Cl_4_{P­(*n*-C_4_H_9_)_3_}_2_] and All-*Trans*-[(Mo_6_Cl_8_)­Cl_2_(C_2_H_5_)_2_{P­(*n*-C_4_H_9_)_3_}_2_].Cntdot.2C_6_H_5_CH_3_. Inorg. Chem..

[20] Sokolov M. N., Mikhailov M. A., Brylev K. A., Virovets A. V., Vicent C., Kompankov N. B., Kitamura N., Fedin V. P. (2013). Alkynyl Complexes
of High-Valence Clusters. Synthesis and Luminescence Properties of
[Mo_6_I_8_(C≡CC­(O)­OMe)_6_]^2–^, the First Complex with Exclusively Organometallic Outer Ligands
in the Family of Octahedral {M_6_X_8_} Clusters. Inorg. Chem..

[21] Soto E., Helmink K. L., Chin C. P., Ferguson M., Peters S. J., Szczepura L. F. (2022). Rhenium
Selenide Clusters Containing Alkynyl Ligands:
Unexpected Reactivity of σ-Bound Phenylacetylide. Organometallics.

[22] Denis R., Toupet L., Paul F., Lapinte C. (2000). Electron-Rich Piano-Stool
Iron σ-Acetylides Bearing a Functional Aryl Group. Synthesis
and Characterization of Iron­(II) and Iron­(III) Complexes. Organometallics.

[23] Paul F., Ellis B. G., Bruce M. I., Toupet L., Roisnel T., Costuas K., Halet J.-F., Lapinte C. (2006). Bonding and Substituent
Effects in Electron-Rich Mononuclear Ruthenium σ-Arylacetylides
of the Formula [(η^2^-Dppe)­(η^5^-C_5_Me_5_)­Ru­(C≡C)-1,4-(C_6_H_4_)­X]­[PF_6_]^n^ (n = 0, 1; X = NO_2_, CN,
F, H, OMe, NH_2_). Organometallics.

[24] Paul F., Mevellec J.-Y., Lapinte C. (2002). Electron-Rich
Fe­(II) and Fe­(III)
Organoiron σ-Alkynyl Complexes Bearing a Functional Aryl Group.
Vibrational Spectroscopic Investigations of the Substituent Effect
on the C≡C Triple Bond. J. Chem. Soc.,
Dalton Trans..

[25] Groom C. R., Bruno I. J., Lightfoot M. P., Ward S. C. (2016). The Cambridge Structural
Database. Acta Crystallogr., Sect. B.

[26] Ozerov O. V., Watson L. A., Pink M., Baik M.-H., Caulton K. G. (2004). Terminal
Acetylenes React to Increase Unsaturation in [(*t*Bu_2_PCH_2_SiMe_2_)_2_N]­Re­(H)_4_. Organometallics.

[27] Gabriel J.-C. P., Boubekeur K., Uriel S., Batail P. (2001). Chemistry of Hexanuclear
Rhenium Chalcohalide Clusters. Chem. Rev..

[28] Yoshimura T., Suo C., Tsuge K., Ishizaka S., Nozaki K., Sasaki Y., Kitamura N., Shinohara A. (2010). Excited-State Properties of Octahedral
Hexarhenium­(III) Complexes with Redox-Active N-Heteroaromatic Ligands. Inorg. Chem..

[29] Szczepura L. F., Cedeno D. L., Johnson D. B., McDonald R., Knott S. A., Jeans K. M., Durham J. L. (2010). Substitution of the Terminal Chloride
Ligands of [Re_6_S_8_Cl_6_]^4–^ with Triethylphosphine: Photophysical and Electrochemical Properties
of a New Series of [Re_6_S_8_]^2+^ Based
Clusters. Inorg. Chem..

[30] Wilson W. B., Stark K., Johnson D. B., Ren Y., Ishida H., Cedeno D. L., Szczepura L. F. (2014). Photophysical
Properties of a Series
of Rhenium Selenide Cluster Complexes Containing Nitrogen-Donor Ligands. Eur. J. Inorg. Chem..

[31] Yoshimura T., Umakoshi K., Sasaki Y., Ishizaka S., Kim H.-B., Kitamura N. (2000). Emission and Metal- and Ligand-Centered-Redox Characteristics
of the Hexarhenium­(III) Clusters *Trans*- and *Cis*-[Re_6_(μ_3_-S)_8_Cl_4_(L)_2_]^2–^, Where L Is a Pyridine
Derivative or Pyrazine. Inorg. Chem..

[32] Yoshimura T., Nishizawa H., Nagata K., Ito A., Sakuda E., Ishizaka S., Kitamura N., Shinohara A. (2022). Tuning the
Ground- and Excited-State Redox Potentials of Octahedral Hexanuclear
Rhenium­(III) Complexes by the Combination of Terminal Halide and N-Heteroaromatic
Ligands. ACS Omega.

[33] Gray T. G., Rudzinski C. M., Meyer E. E., Holm R. H., Nocera D. G. (2003). Spectroscopic
and Photophysical Properties of Hexanuclear Rhenium­(III) Chalcogenide
Clusters. J. Am. Chem. Soc..

[34] Kitamura N., Ueda Y., Ishizaka S., Yamada K., Aniya M., Sasaki Y. (2005). Temperature Dependent
Emission of Hexarhenium­(III)
Clusters [Re_6_(μ_3_-S)_8_X_6_]^4–^ (X = Cl^–^, Br^–^, and I^–^): Analysis by Four Excited Triplet-State
Sublevels. Inorg. Chem..

[35] Gray T. G., Rudzinski C. M., Nocera D. G., Holm R. H. (1999). Highly Emissive
Hexanuclear Rhenium­(III) Clusters Containing the Cubic Cores [Re_6_S_8_]^2+^ and [Re_6_Se_8_]^2+^. Inorg. Chem..

[36] Molard Y., Dorson F., Brylev K. A., Shestopalov M. A., Le Gal Y., Cordier S., Mironov Y. V., Kitamura N., Perrin C. (2010). Red-NIR Luminescent Hybrid Poly­(Methyl
Methacrylate)
Containing Covalently Linked Octahedral Rhenium Metallic Clusters. Chem. – Eur. J..

[37] Carrasco I., Ehni P., Ebert M., Dumait N., Taupier G., Amela-Cortes M., Roiland C., Cordier S., Knöller J. A., Jacques E., Laschat S., Molard Y. (2023). Game of Crowns: Na^+^ Is Coming! Red NIR-Emissive Hybrid Liquid Crystals Containing
Discotic Crown Ethers and Na_2_Mo_6_X_8_
^i^Cl_6_ (X^i^ = Cl or Br). ACS Appl. Mater. Interfaces.

[38] Fišerová E., Kubala M. (2012). Mean Fluorescence
Lifetime and Its Error. J. Lumin..

[39] Yoshimura T., Chen Z.-N., Itasaka A., Abe M., Sasaki Y., Ishizaka S., Kitamura N., Yarovoi S. S., Solodovnikov S. F., Fedorov V. E. (2003). Preparation Structures, and Redox and Emission Characteristics
of the Isothiocyanate Complexes of Hexarhenium­(III) Clusters [Re_6_(μ_3_-E)_8_(NCS)_6_]^4–^ (E = S, Se). Inorg. Chem..

[40] Ivanov A.
A., Shestopalov M. A., Brylev K. A., Khlestkin V. K., Mironov Y. V. (2014). A Family of Octahedral
Rhenium Cluster Complexes Trans-[{Re_6_Q_8_}­(PPh_3_)_4_X_2_]
(Q = S or Se, X = Cl, Br or I): Preparation and Halide-Dependent Luminescence
Properties. Polyhedron.

[41] Mikhailov M. A., Brylev K. A., Abramov P. A., Sakuda E., Akagi S., Ito A., Kitamura N., Sokolov M. N. (2016). Synthetic Tuning of Redox, Spectroscopic,
and Photophysical Properties of {Mo_6_I_8_}^4+^ Core Cluster Complexes by Terminal Carboxylate Ligands. Inorg. Chem..

[42] Zheng Z., Long J. R., Holm R. H. (1997). A Basis
Set of Re_6_Se_8_ Cluster Building Blocks and Demonstration
of Their Linking
Capability: Directed Synthesis of an Re_12_Se_16_ Dicluster. J. Am. Chem. Soc..

[43] Long J. R., McCarty L. S., Holm R. H. (1996). A Solid-State
Route to Molecular
Clusters: Access to the Solution Chemistry of [Re_6_Q_8_]^2+^ (Q = S, Se) Core-Containing Clusters via Dimensional
Reduction. J. Am. Chem. Soc..

[44] Sheldrick G. M. (2015). SHELXT
– Integrated Space-Group and Crystal-Structure Determination. Found. Crystallogr..

[45] Dolomanov O. V., Bourhis L. J., Gildea R. J., Howard J. A. K., Puschmann H. (2009). OLEX2: A Complete
Structure Solution, Refinement and Analysis Program. J. Appl. Crystallogr..

[46] Sheldrick G. M. (2008). A Short
History of SHELX. Acta Crystallogr., Sect. A.

[47] Van
der Sluis P., Spek A. L. (1990). BYPASS: An Effective Method for the
Refinement of Crystal Structures Containing Disordered Solvent Regions. Acta Crystallogr., Sect. A.

[48] Neese F. (2012). The ORCA Program
System. Wiley Interdiscip. Rev.: Comput. Mol.
Sci..

[49] Neese F., Wennmohs F., Becker U., Riplinger C. (2020). The ORCA Quantum
Chemistry Program Package. J. Chem. Phys..

[50] Neese F. (2018). Software Update:
The ORCA Program System, Version 4.0. Wiley
Interdiscip. Rev.: Comput. Mol. Sci..

[51] Pantazis D. A., Chen X.-Y., Landis C. R., Neese F. (2008). All-Electron Scalar
Relativistic Basis Sets for Third-Row Transition Metal Atoms. J. Chem. Theory Comput..

[52] Bühl M., Reimann C., Pantazis D. A., Bredow T., Neese F. (2008). Geometries
of Third-Row Transition-Metal Complexes from Density-Functional Theory. J. Chem. Theory Comput..

[53] van
Wüllen C. (1998). Molecular Density Functional Calculations in the Regular
Relativistic Approximation: Method, Application to Coinage Metal Diatomics,
Hydrides, Fluorides and Chlorides, and Comparison with First-Order
Relativistic Calculations. J. Chem. Phys..

[54] Perdew J.
P., Yue W. (1986). Accurate and
Simple Density Functional for the Electronic Exchange
Energy: Generalized Gradient Approximation. Phys. Rev. B.

[55] Becke A. D. (1988). Density-Functional
Exchange-Energy Approximation with Correct Asymptotic Behavior. Phys. Rev. A.

