# Supplementary material for: Rhenium Chalcogenide Clusters Containing para-Substituted Phenylacetylide Ligands: Synthesis, Characterization, and Investigation of Substituent Effects on Spectroscopic and Electrochemical Properties
Source: Organometallics. 2025 Sep 25;44(19):2209–18. doi: 10.1021/acs.organomet.5c00245 (PMC12522138; doi:10.1021/acs.organomet.5c00245)

Supporting Information

Rhenium chalcogenide clusters containing *para*-substituted phenylacetylide ligands: Synthesis, characterization, and investigation into substituent effects on spectroscopic and electrochemical properties

Katherine L. Helmink[a], Cory A. Hicks[a], B. Sage Lauper-Cook[a], Steven J. Peters[a], Christopher J. A. Daley[b], Yann Molard[c] and Lisa F. Szczepura[a]*

[a]*Department of Chemistry, Illinois State University, Normal, IL 61790-4160, United States.*
[b]*Department of Chemistry and Biochemistry, University of San Diego, 5998 Alcal´a Park, San Diego, CA 92110, United States.*
[c]*Université de Rennes, CNRS, ISCR – UMR 6226, ScanMAT – UAR 2025, Rennes F-35000, France.*
*Email: lfszcze@ilstu.edu

Table of Contents

Table S1. Crystallographic Data S2

Figure S1. Cyclic voltammogram of **2** (black) and **4** (red). S3

Figure S2. Cyclic voltammogram of **1** (full scan) in MeCN. S4

Figure S3. Plot of peak oxidation potential ($E_{p,a}$) vs Hammett parameter σ_p. S5

Figure S4. Emission spectra for compounds **1** – **7** in powder phase. S6

Figure S5. Correlation between ln k_{nr} and E_{em} for compounds **1** – **7** in MeCN. S7

Figure S6. Hammett correlation between k_{nr} and σ_p for compounds **1** - **5**. S8

Figure S7. Hammett correlation between τ and σ_p for compounds **1** – **5**. S9

Figures S8 – S12. HOMO LUMO Diagrams of PMe_3 analogs of **1** – **5**. S10 – S14

Characterization data for **1**, **2**, **4**, and **5** (**Figures S13 – S32**) S15 – S34

Emission Data (**Figures S33- S53**) S35 – S48

Table S1. Crystallographic Data for $[Re_6Se_8(PEt_3)_5(C{\equiv}C\text{-}C_6H_4\text{-}NO_2)](SbF_6)$ (**1**), $[Re_6Se_8(PEt_3)_5(C{\equiv}C\text{-}C_6H_4\text{-}CO_2Me)](SbF_6)$ (**2**), and $(Re_6Se_8(PEt_3)_5(C{\equiv}C\text{-}C_6H_4\text{-}CH_3)](SbF_6)$ (**4**).

	**1**	**2**[a]	**4**[b]
Formula	$C_{38}H_{79}F_6NO_2P_5Re_6SbSe_8$	$C_{50}H_{96}F_6O_3P_5Re_6SbSe_8$	$C_{53}H_{98.75}F_6O_{0.38}P_5Re_6SbSe_8$
Fw	2721.50	2884.74	2881.62
Cryst. Syst.	Monoclinic	Orthorhombic	Triclinic
Space group	P2/c	$P2_12_12_1$	P–1
a (Å)	15.8193(7)	15.9835(12)	16.3284(9)
b (Å)	11.6352(5)	19.5895(14)	20.2337(11)
c (Å)	34.2693(15)	22.3835(15)	22.8800(13)
α (deg)	90	90	109.755(2)
β (deg)	92.0646(7)	90	91.923(2)
γ (deg)	90	90	94.096(2)
Volume ($Å^3$)	6303.5(5)	7008.5(9)	7082.7(7)
T (K)	173.15	173.00	173.00
Z	4	4	4
ρ_{calc} (g/cm^3)	2.866	2.734	2.702
$\mu_{Mo\ K\alpha}$ (mm^{-1})	16.691	15.021	14.861
F(000)	4928	5288	5287
crystal size (mm^3)	0.182 x 0.104 x 0.047	0.154 x 0.056 x 0.055	0.202 x 0.136 x 0.024
θ scan range (deg)	1.288 – 27.508	1.879 – 28.343	1.983 – 26.500
Index ranges	$-20 \leq h \leq 20$<br>$-15 \leq k \leq 15$<br>$-44 \leq l \leq 44$	$-21 \leq h \leq 20$<br>$-26 \leq k \leq 24$<br>$-29 \leq l \leq 29$	$-20 \leq h \leq 20$<br>$-25 \leq k \leq 25$<br>$-28 \leq l \leq 28$
reflections collected	56337	82310	422138
indepn reflections	14489 [R(int) = 0.0275]	17438 [R(int) = 0.0703]	29354 [R(int) = 0.0608]
GOF on F^2	1.037	1.119	1.081
final R [$I \geq 2\sigma(I)$]	R1 = 0.0231,<br>*w*R2 = 0.0475	R1 = 0.0515,<br>*w*R2 = 0.1266	R1 = 0.0392,<br>*w*R2 = 0.1015
final R [all data]	R1 = 0.0312,<br>*w*R2 = 0.0499	R1 = 0.0860,<br>*w*R2 = 0.1567	R1 = 0.0511,<br>*w*R2 = 0.1198
$\Delta\rho$ (e $Å^{-3}$), min/max	3.196 / –2.126	2.627 / –1.803	3.355 / –2.023

[a] Solvent mask was applied via BYPASS[1] to account for disordered toluene (x 1) and acetone (x 1) solvent per asymmetric unit.

[b] Solvent mask was applied via BYPASS [1] to account for disordered toluene (x 2) and water (x 0.375) solvent per asymmetric unit

[1]Van der Sluis, P.; Spek, A. L. *Acta Cryst.* **1990**, *A46*, 194-201.

Figure S1. Cyclic voltammogram of **2** (black) and **4** (red) in deaerated 0.2 M Bu_4NBF_4 MeCN at 100 mV/sec.

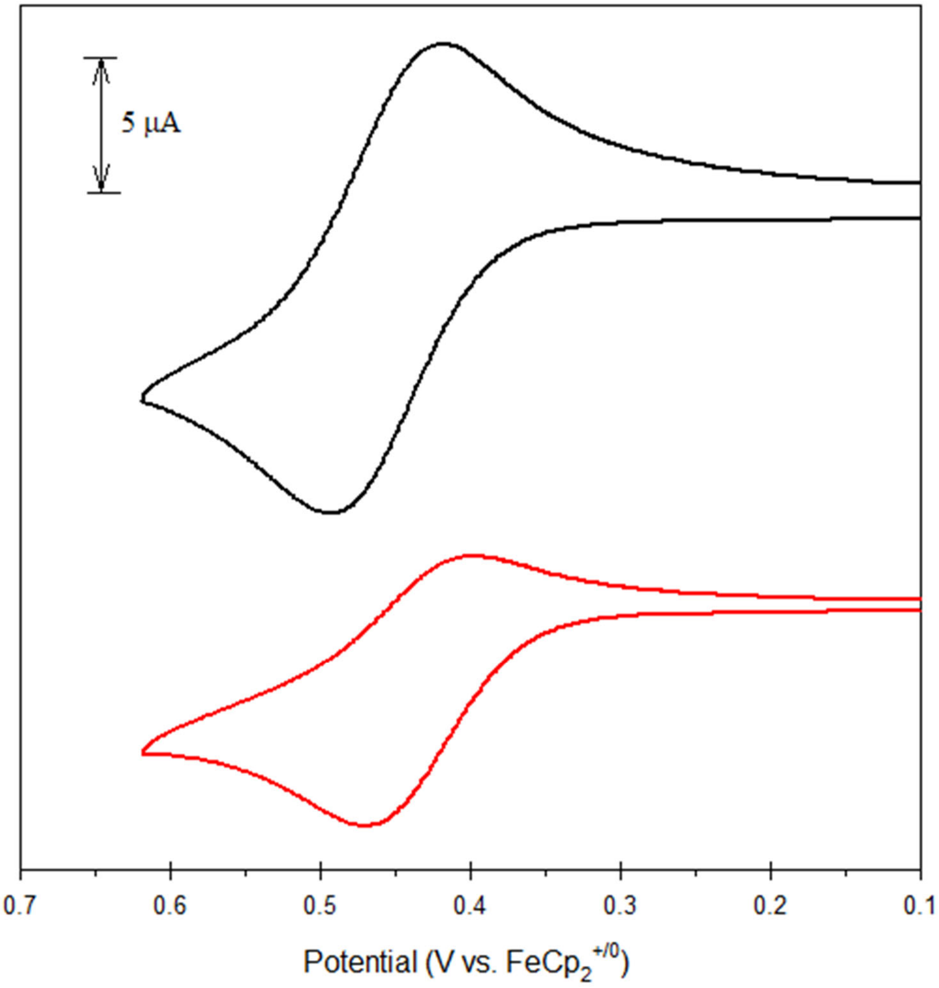

Figure S2. Cyclic voltammogram of **1** (full scan) in deaerated 0.2 M Bu_4NBF_4 MeCN at 100 mV/sec.

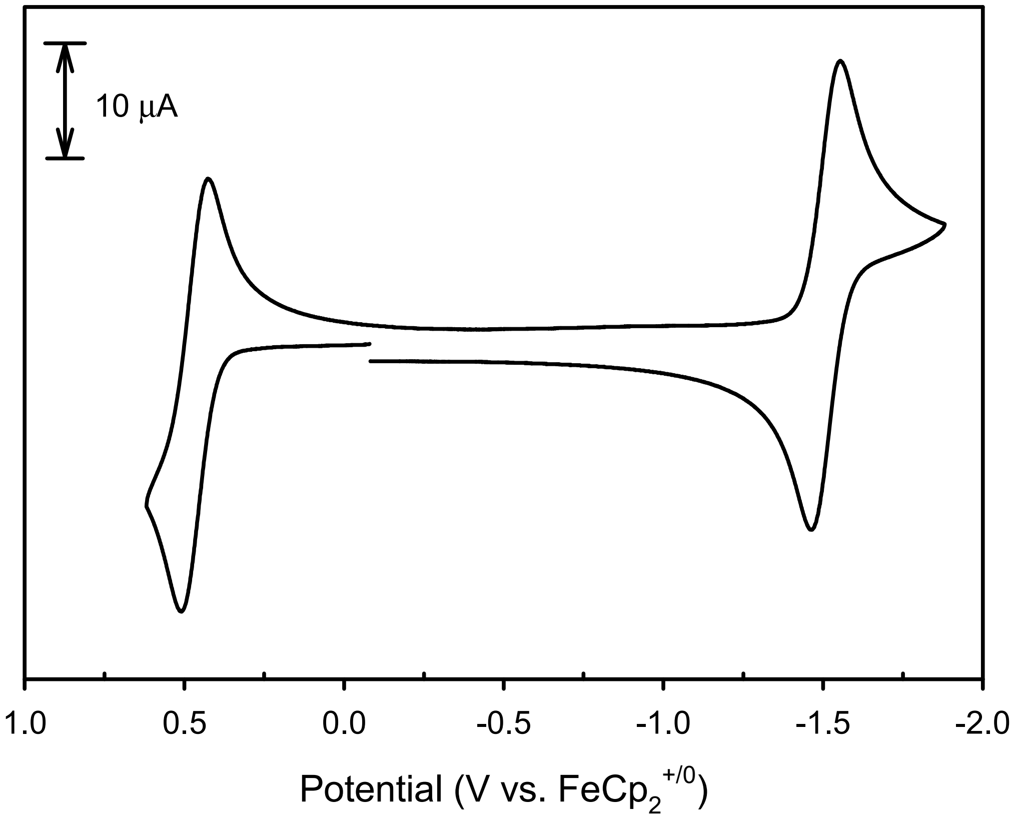

Figure S3. Plot of peak oxidation potential ($E_{p,a}$) vs Hammett parameter σ_p for **1** – **5**.

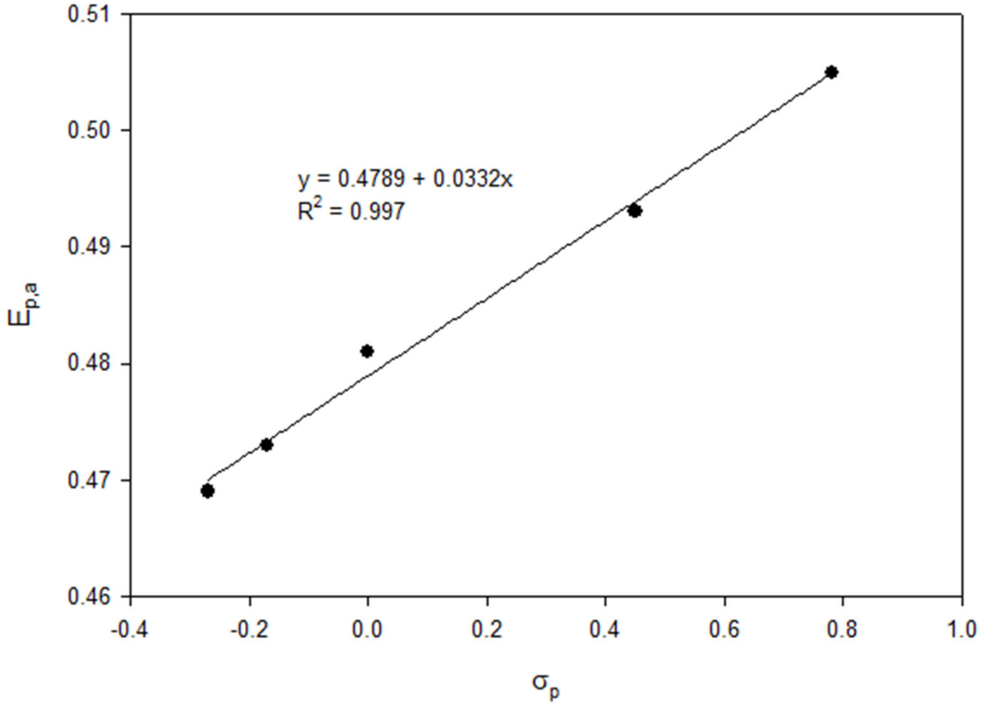

Figure S4. Emission spectra for compounds **1** – **7** in powder phase.

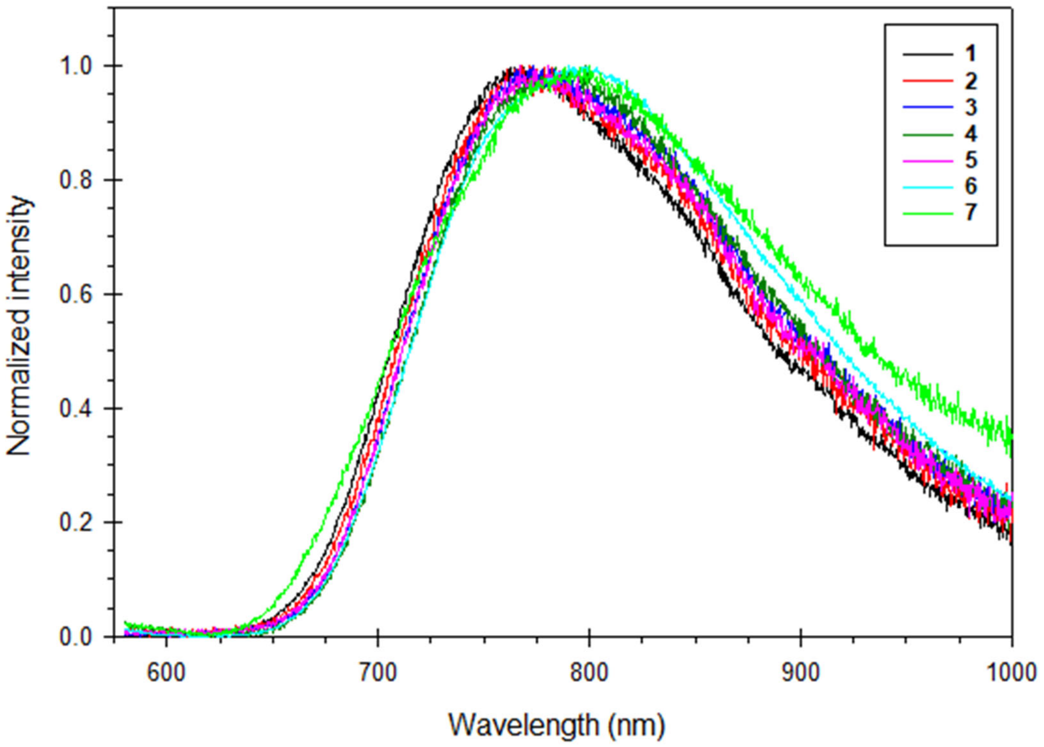

Figure S5. Correlation between ln k_{nr} and E_{em} for compounds **1** – **7**.

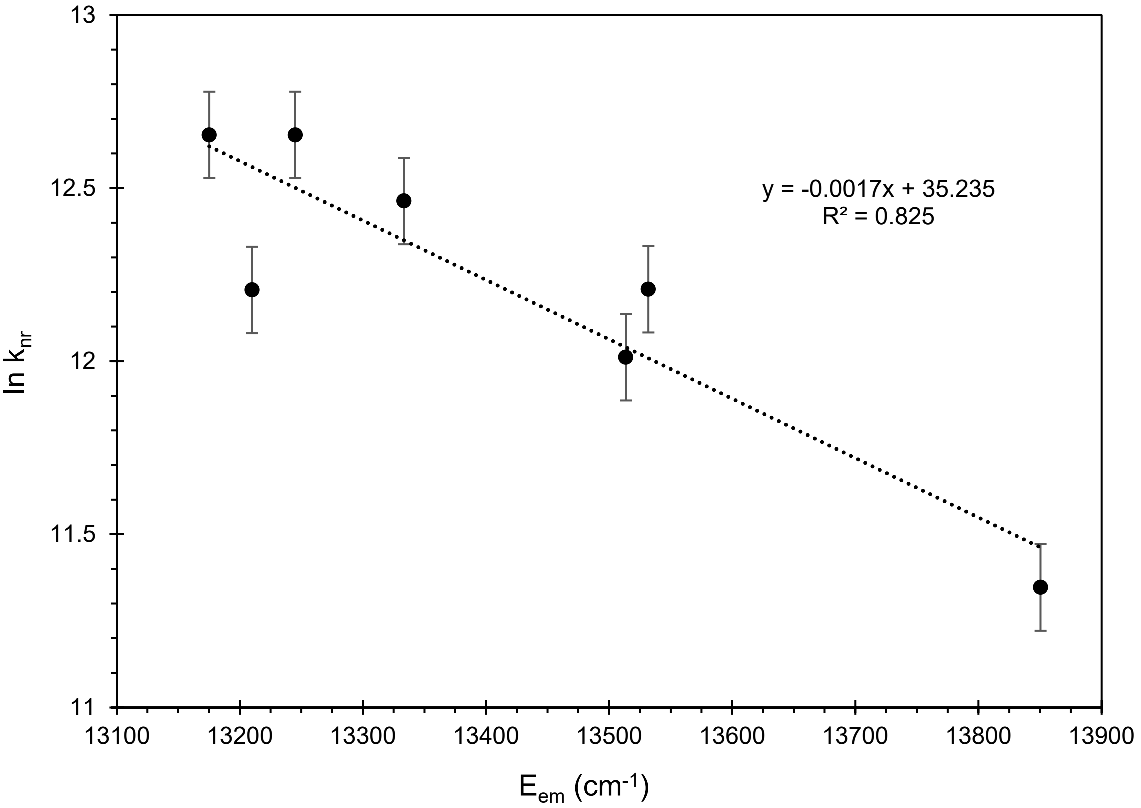

Figure S6. Hammett correlation between k_{nr} and σ_p for compounds **1 - 5**.

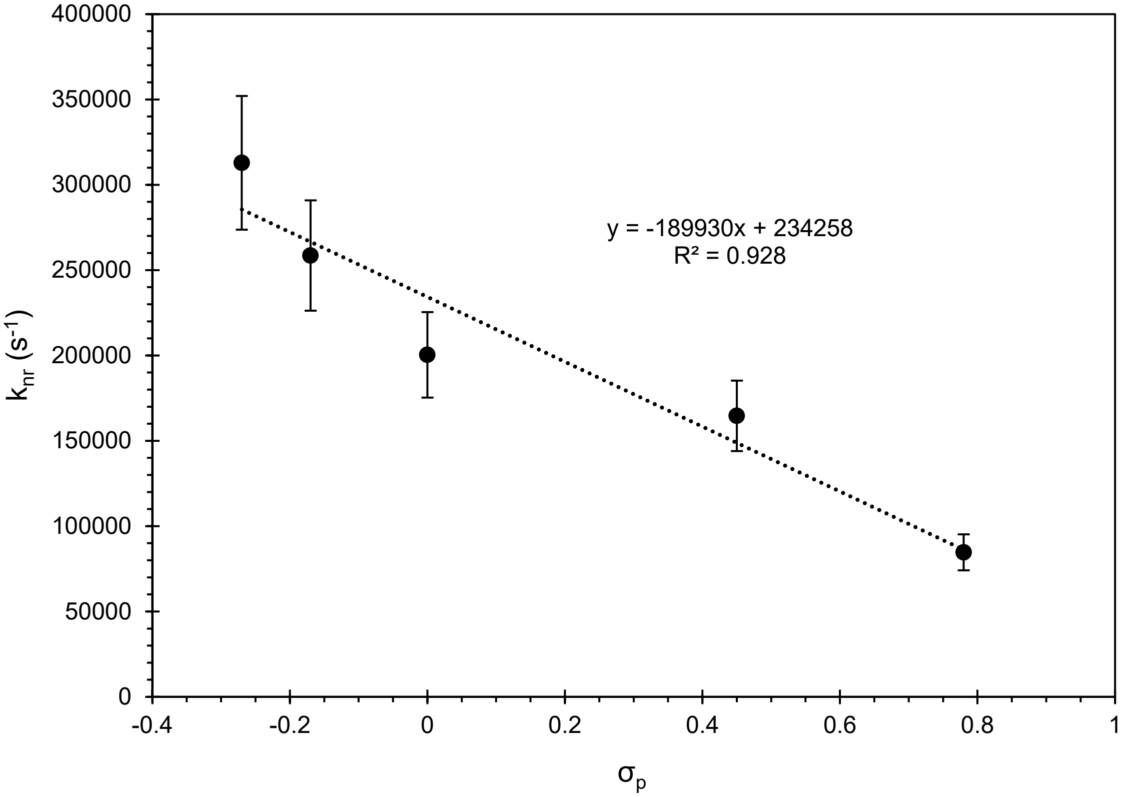

Figure S7. Hammett correlation between τ and σ_p for compounds **1** – **5**.

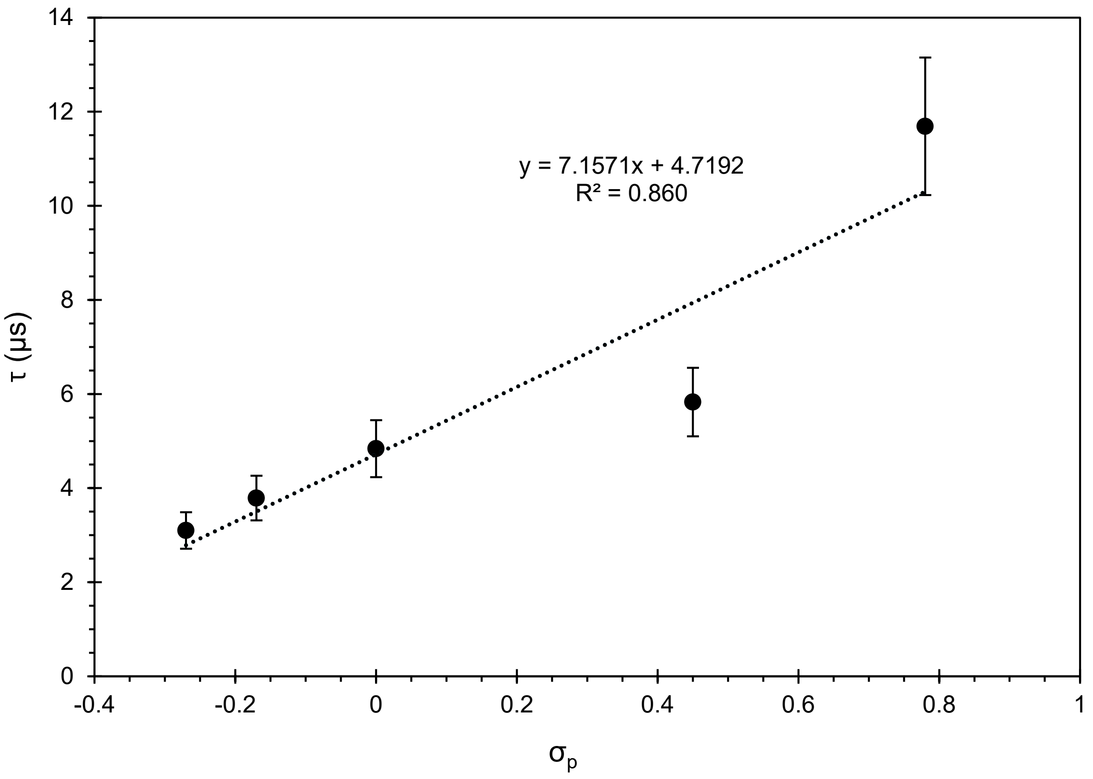

Computational Data for 1-5

Figure S8. HOMO (top) and LUMO (bottom) for the PMe_3 analogue of **1** $[Re_6Se_8(PEt_3)_5(C{\equiv}C\text{-}C_6H_4\text{-}NO_2)]^+$ from RHF/DFT ZORA calculations.

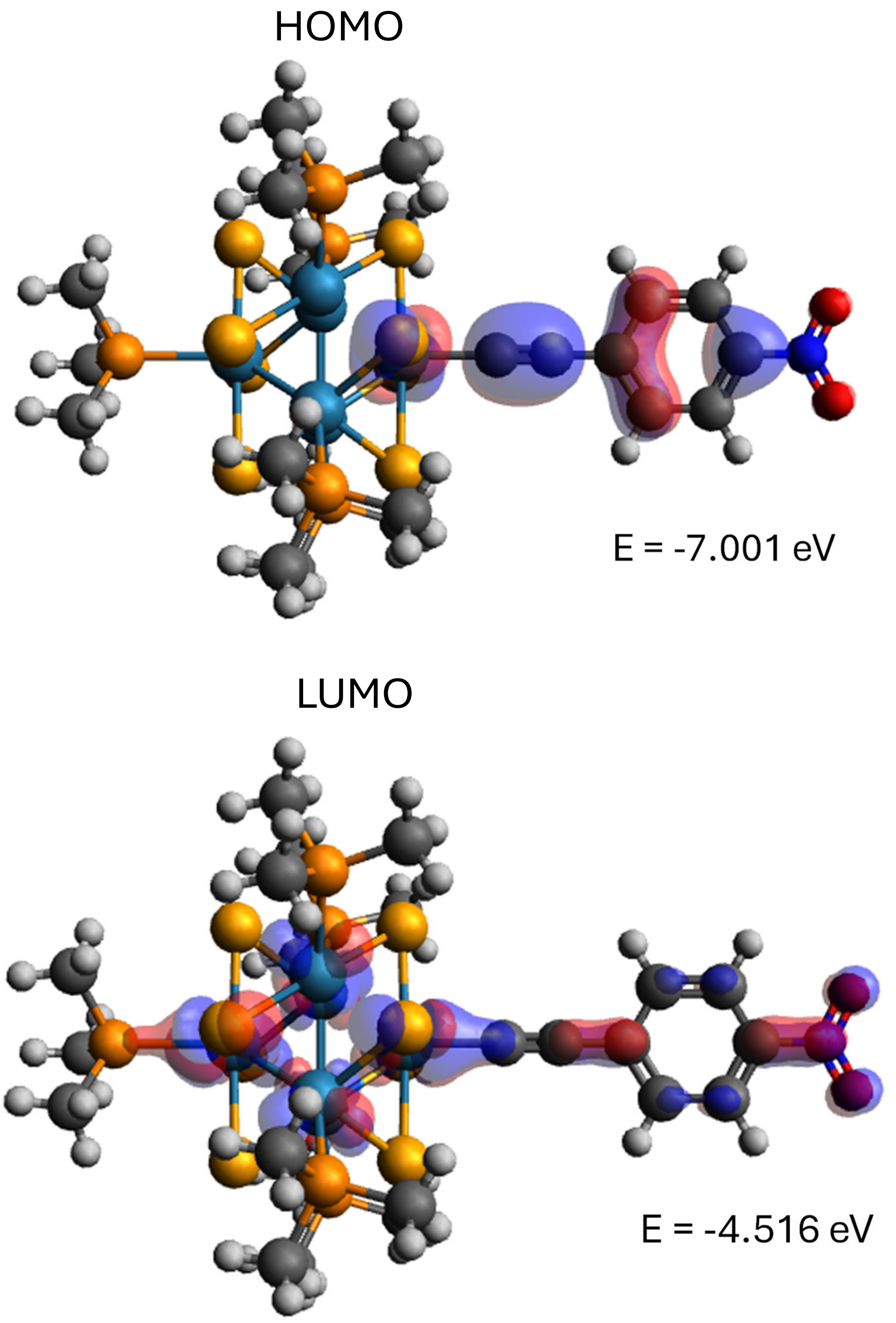

Figure S9. HOMO (top) and LUMO (bottom) for the PMe_3 analogue of **2** $[Re_6Se_8(PEt_3)_5(C{\equiv}C\text{-}C_6H_4\text{-}CO_2Me)]^+$ from RHF/DFT ZORA calculations.

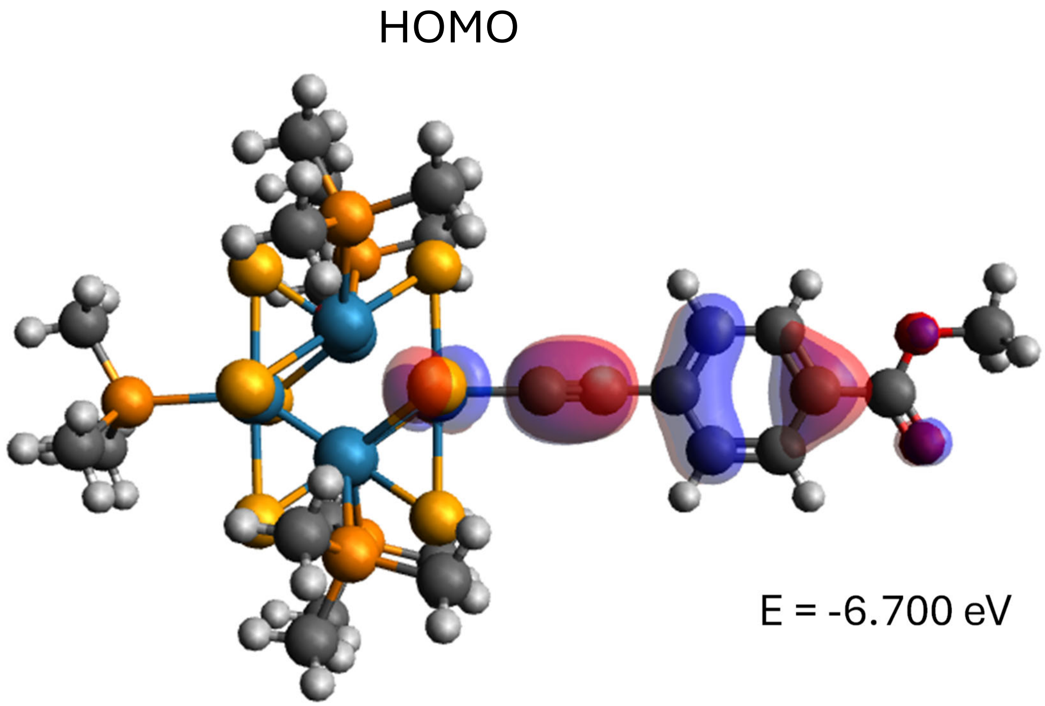

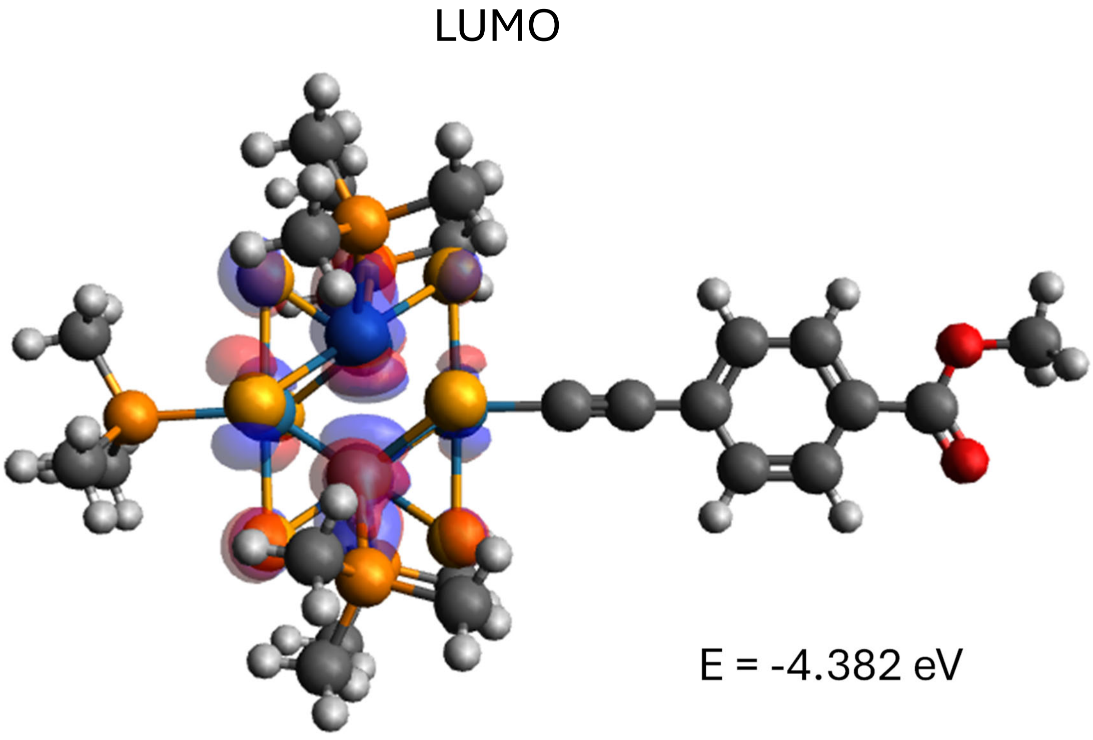

Figure S10. HOMO (top) and LUMO (bottom) for the PMe_3 analogue of **3** $[Re_6Se_8(PEt_3)_5(C{\equiv}CC_6H_5)]^+$ from RHF/DFT ZORA calculations.

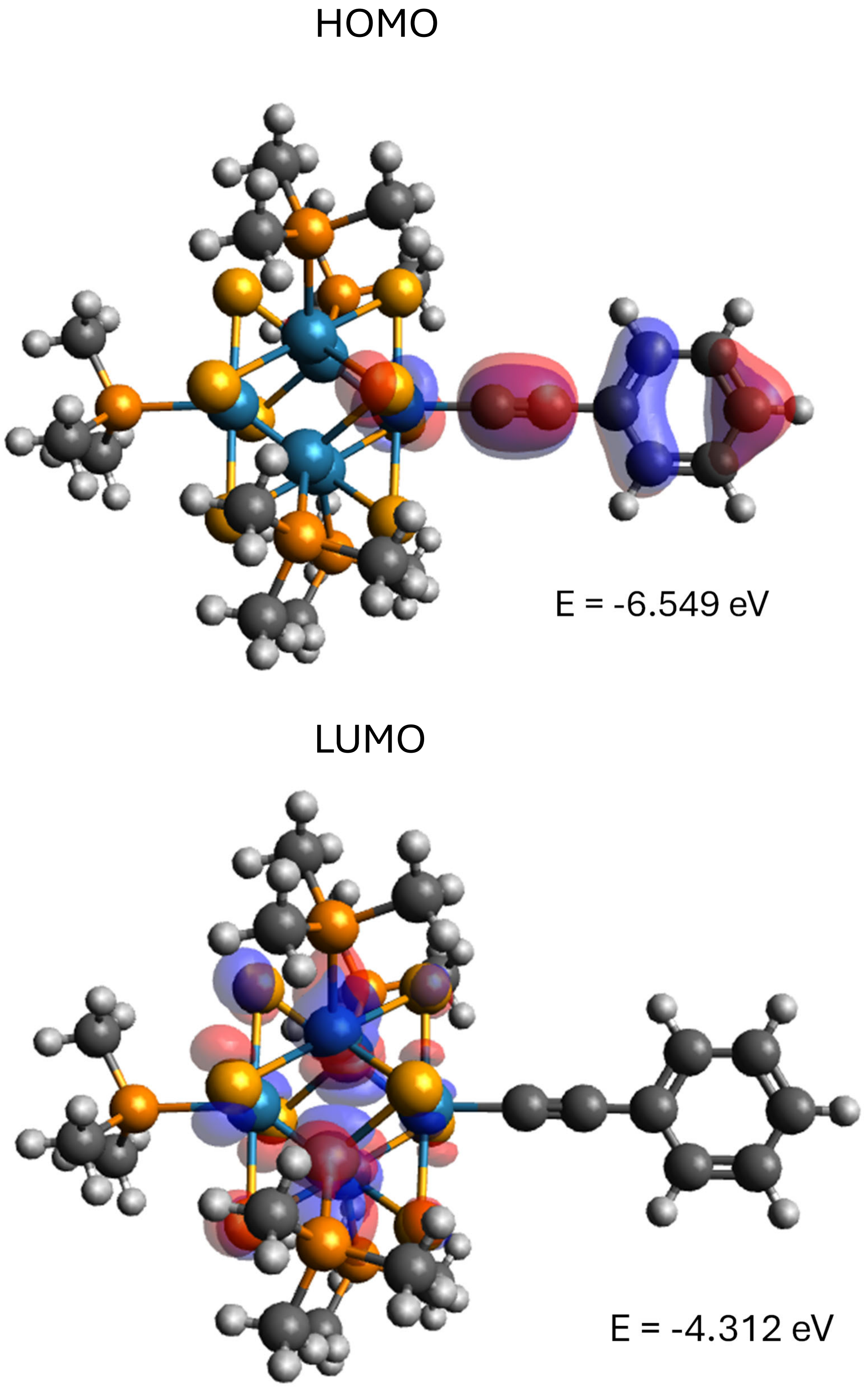

Figure S11. HOMO (top) and LUMO (bottom) for the PMe_3 analogue of **4** $[Re_6Se_8(PEt_3)_5(C{\equiv}C\text{-}C_6H_4\text{-}CH_3)]^+$ from RHF/DFT ZORA calculations.

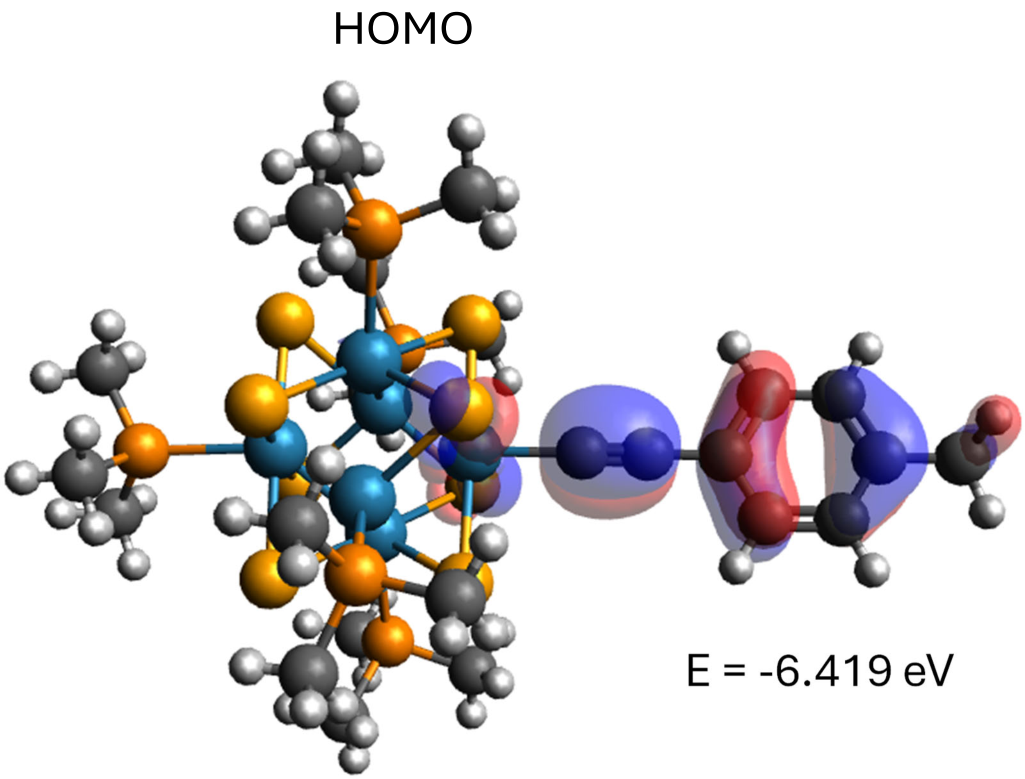

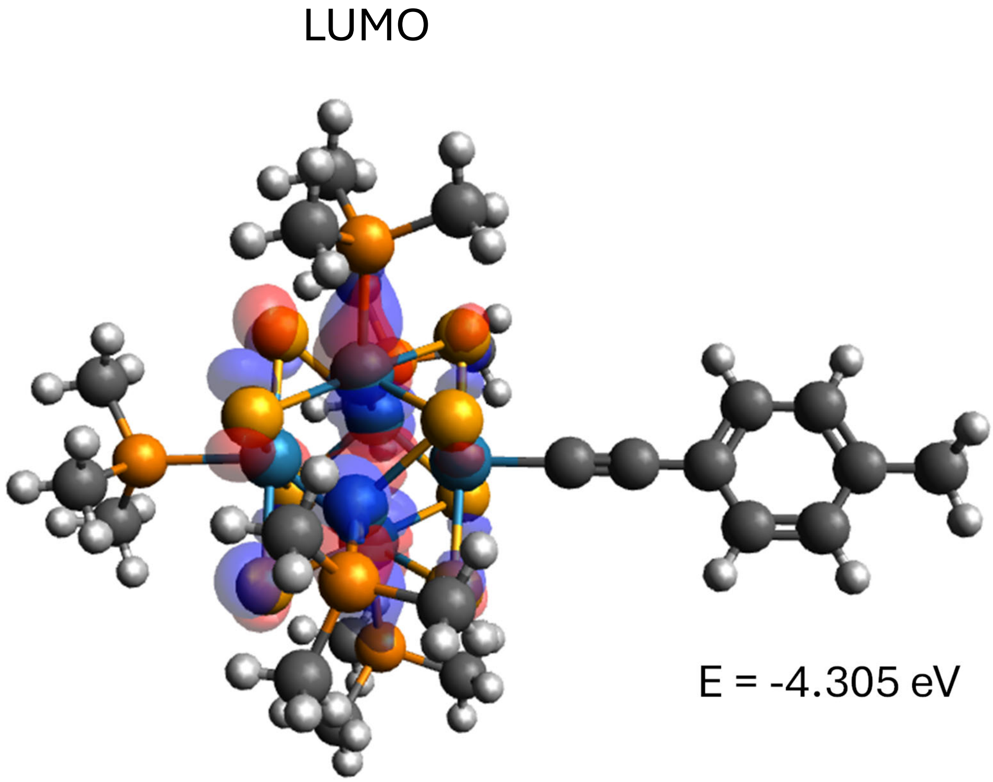

Figure S12. HOMO (top) and LUMO (bottom) for the PMe_3 analogue of **5** $[Re_6Se_8(PEt_3)_5(C{\equiv}C\text{-}C_6H_4\text{-}OMe)]^+$ from RHF/DFT ZORA calculations.

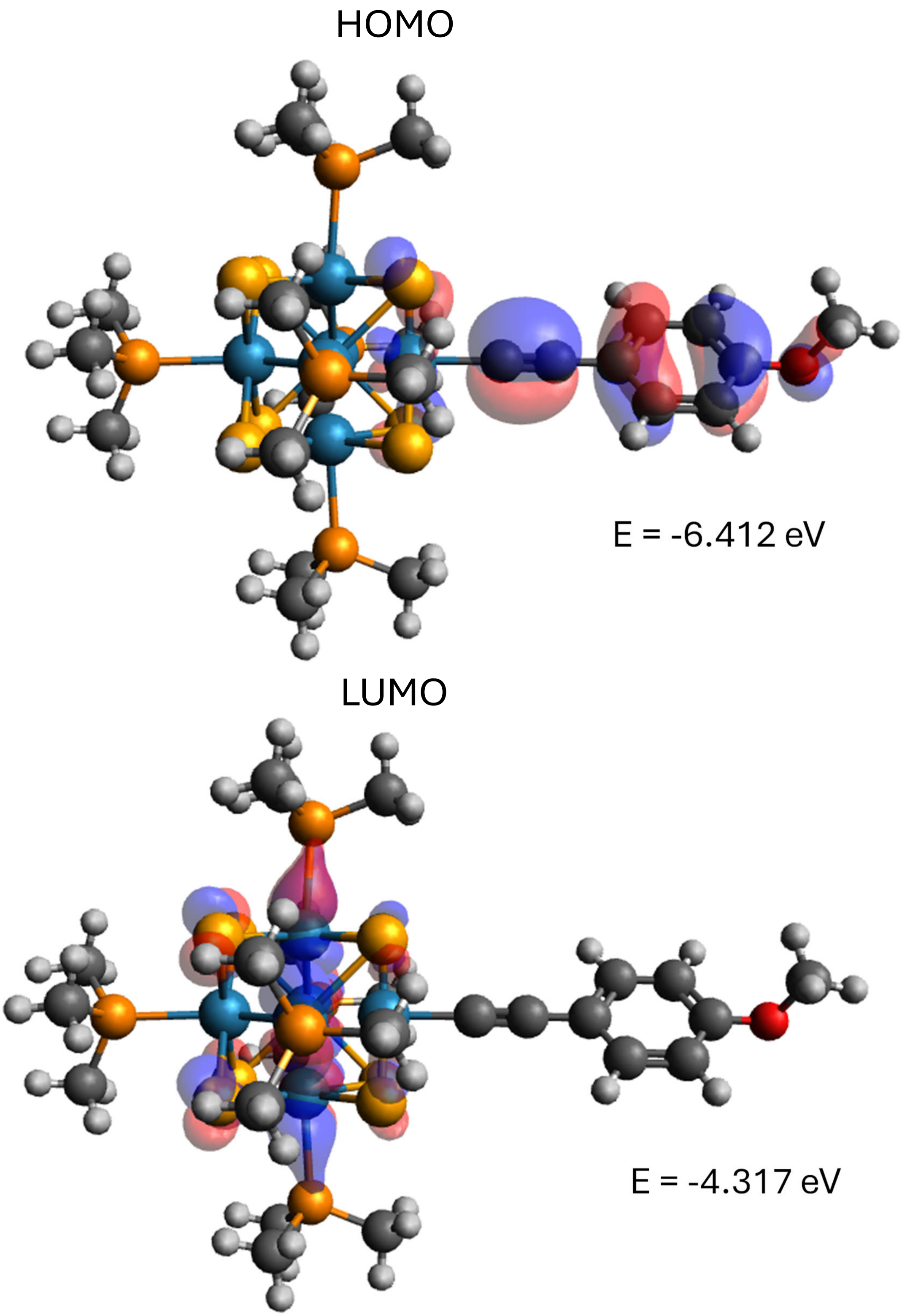

Characterization Data of 1, 2, 4, and 5

Figure S13. 500 MHz ^{1}H NMR spectrum of **1** $[Re_6Se_8(PEt_3)_5(C{\equiv}C\text{-}C_6H_4\text{-}NO_2)](SbF_6)$ in acetone-d_6 (solvent and water peaks are noted with an asterisk).

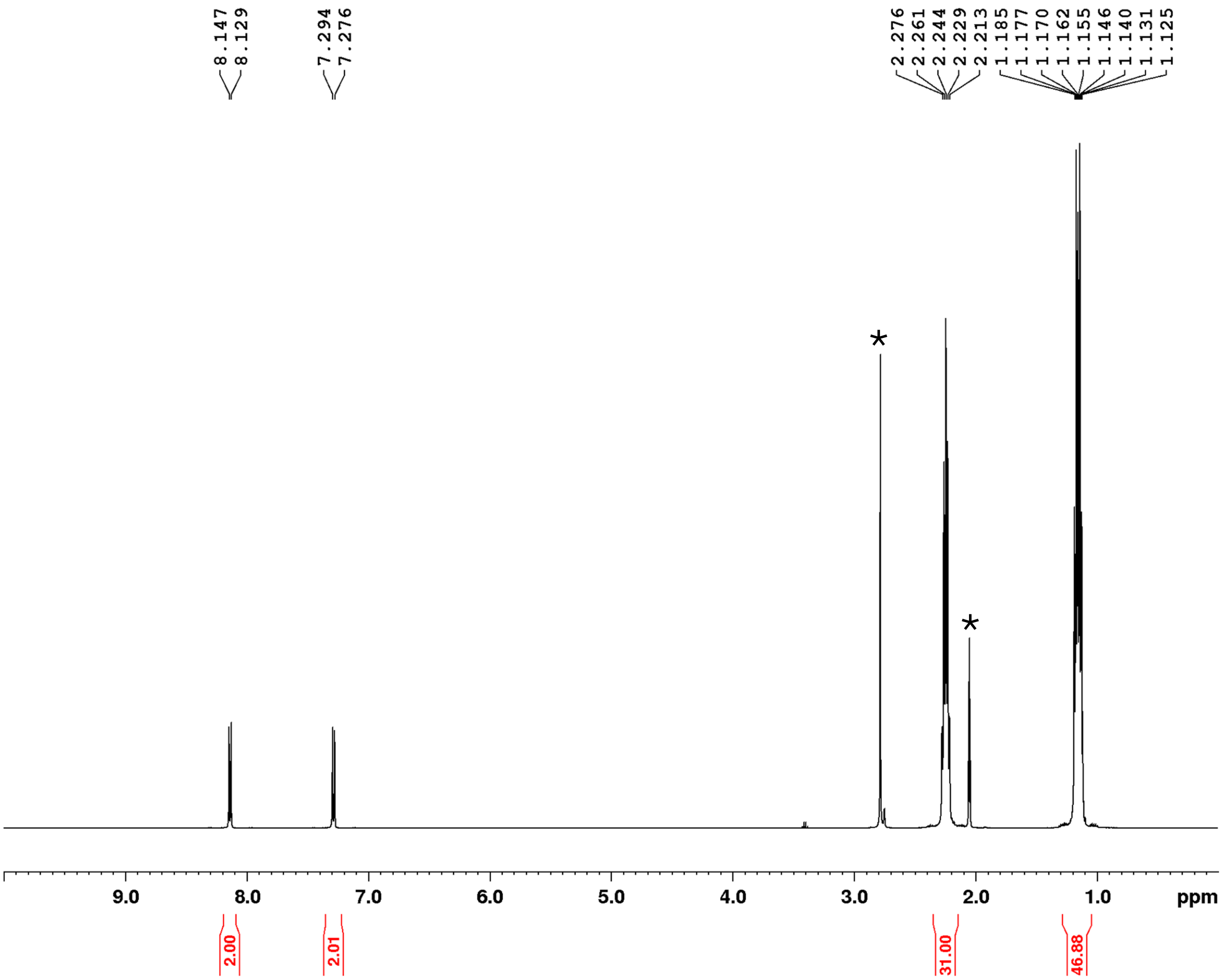

Figure S14. 202.5 MHz $^{31}P\{^{1}H\}$ NMR spectrum of **1** $[Re_6Se_8(PEt_3)_5(C{\equiv}C\text{-}C_6H_4\text{-}NO_2)](SbF_6)$ in acetone-d_6.

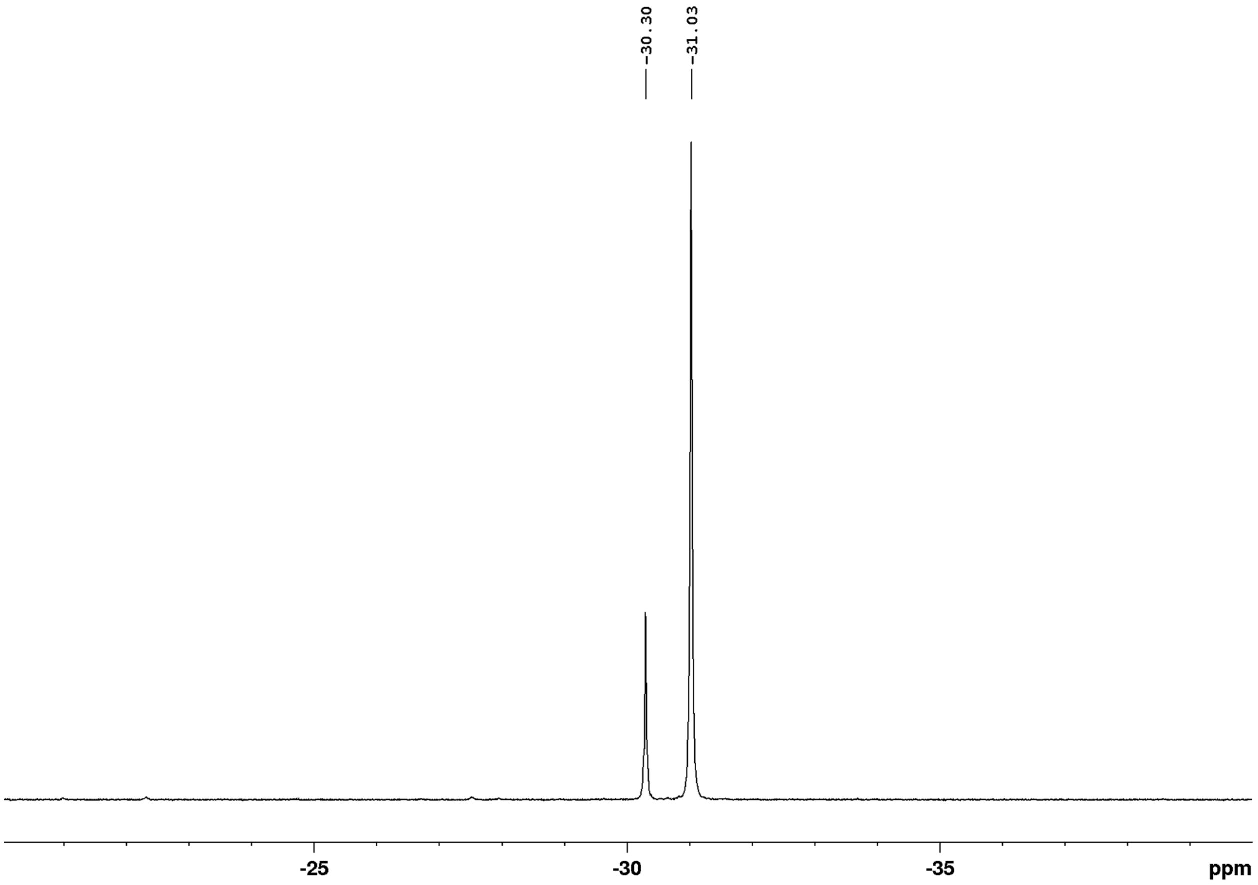

Figure S15. 125 MHz $^{13}C\{^1H\}$ NMR spectrum of **1** $[Re_6Se_8(PEt_3)_5(C{\equiv}C\text{-}C_6H_4\text{-}NO_2)](SbF_6)$ in acetone-d_6 (solvent peaks are noted with an asterisk). The insets show C_α at 102.8 ppm as well as an expanded region around 135 ppm.

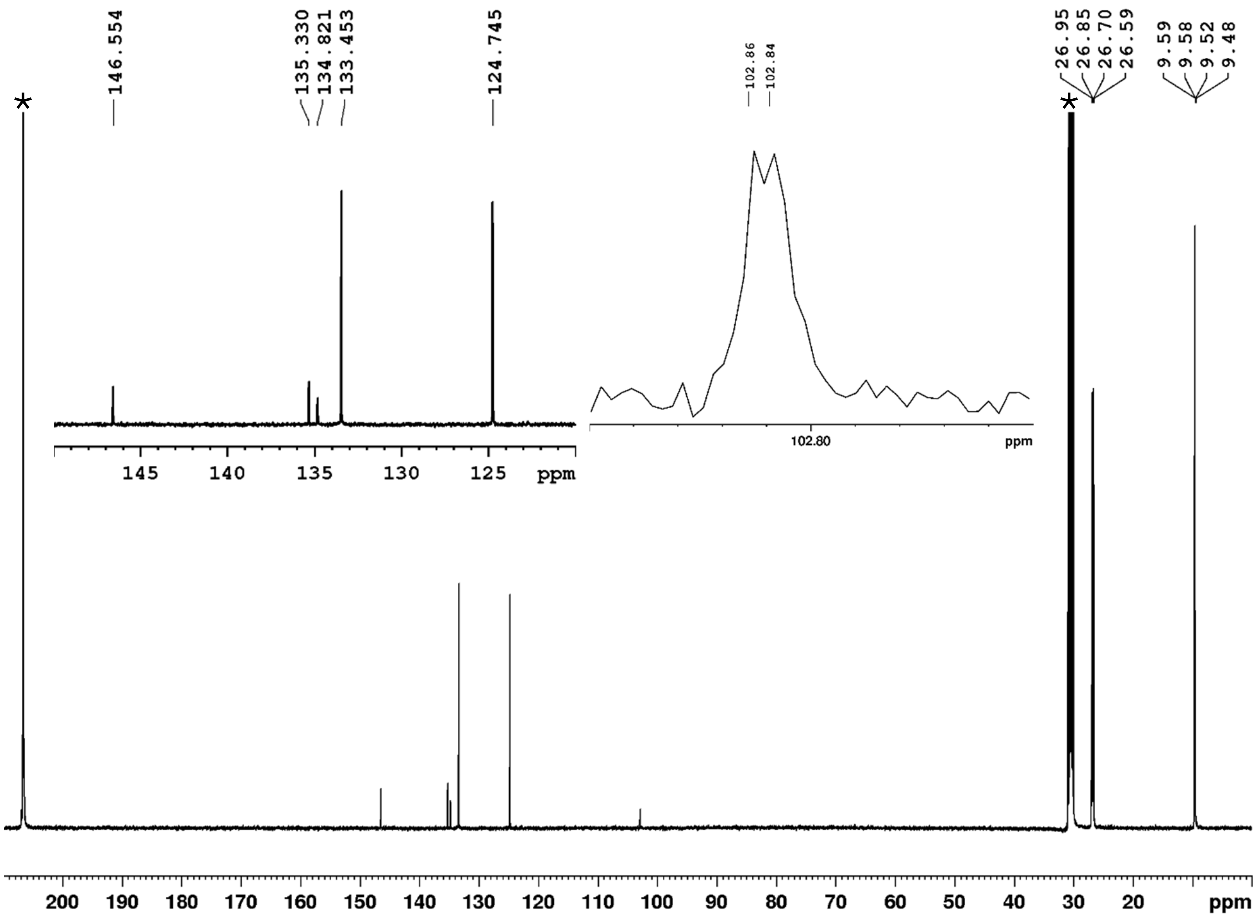

Figure S16. a) Experimental ESI mass spectrometry data and b) theoretical distribution of **1** $[Re_6Se_8(PEt_3)_5(C{\equiv}C\text{-}C_6H_4\text{-}NO_2)]^+$.

a)

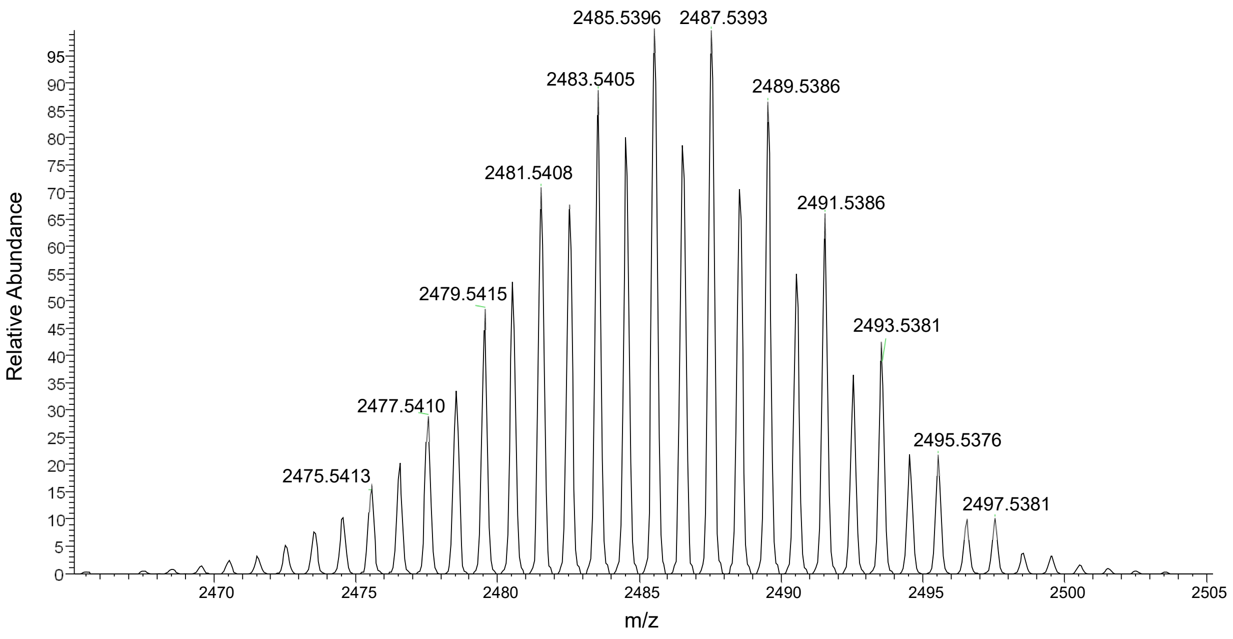

b)

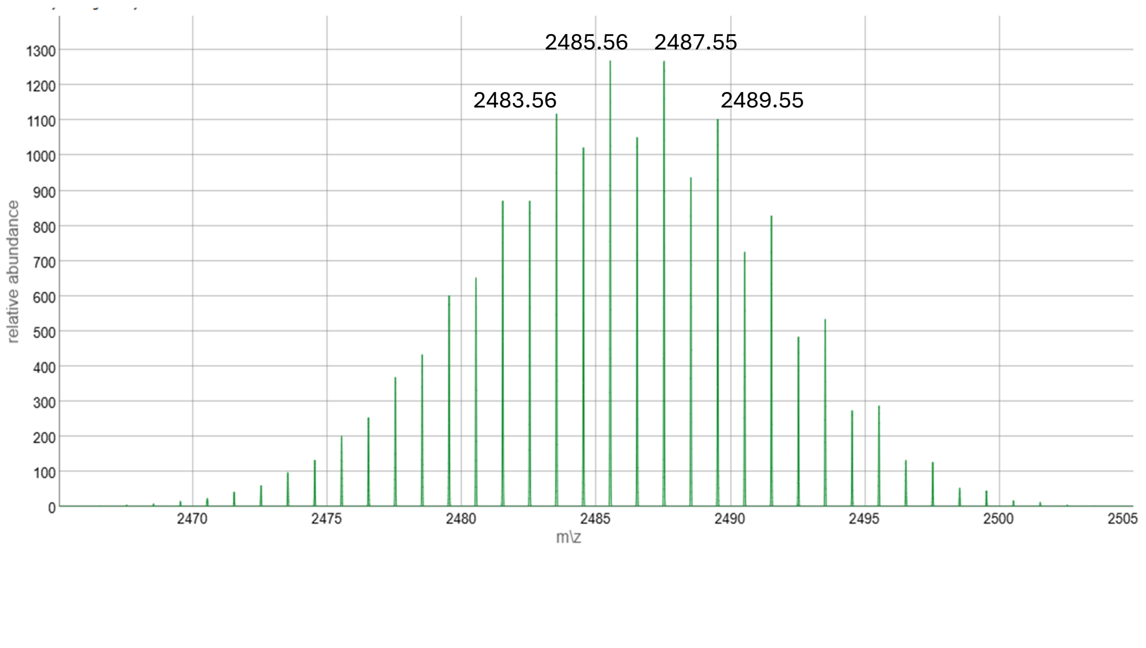

Figure S17. IR-ATR (ZnSe) spectrum of **1** [$Re_6Se_8(PEt_3)_5(C{\equiv}C\text{-}C_6H_4\text{-}NO_2)$]($SbF_6$).

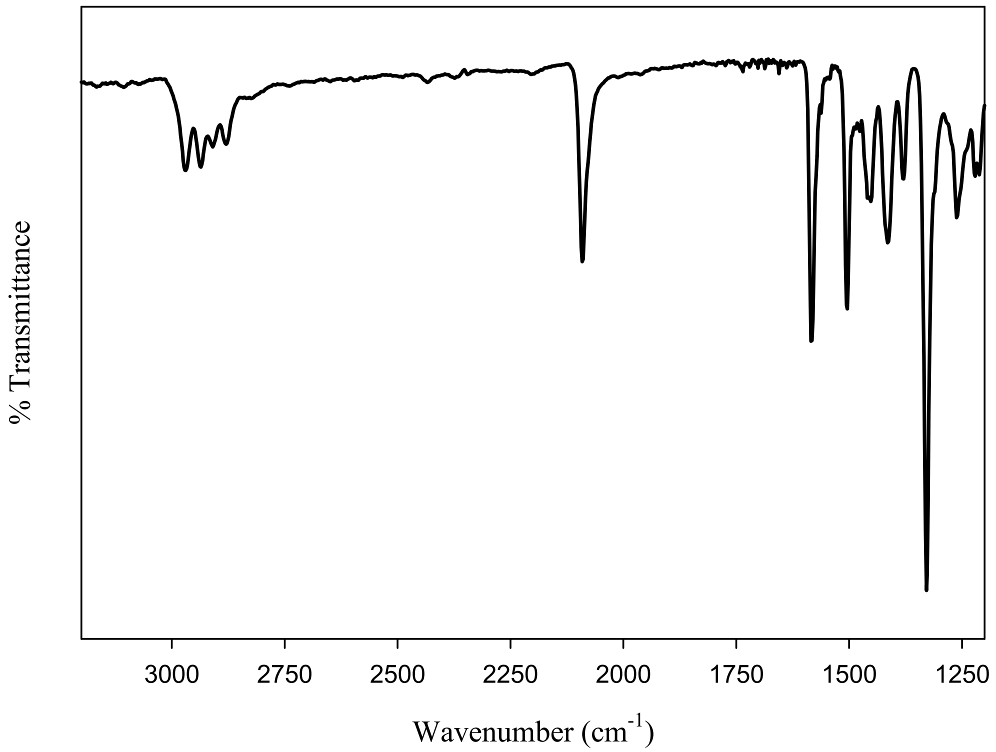

Figure S18. 500 MHz ^{1}H NMR spectrum of **2** [$Re_6Se_8(PEt_3)_5(C{\equiv}C\text{-}C_6H_4\text{-}CO_2Me)$]($SbF_6$) in acetone-$d_6$ (solvent and water peaks are noted with an asterisk).

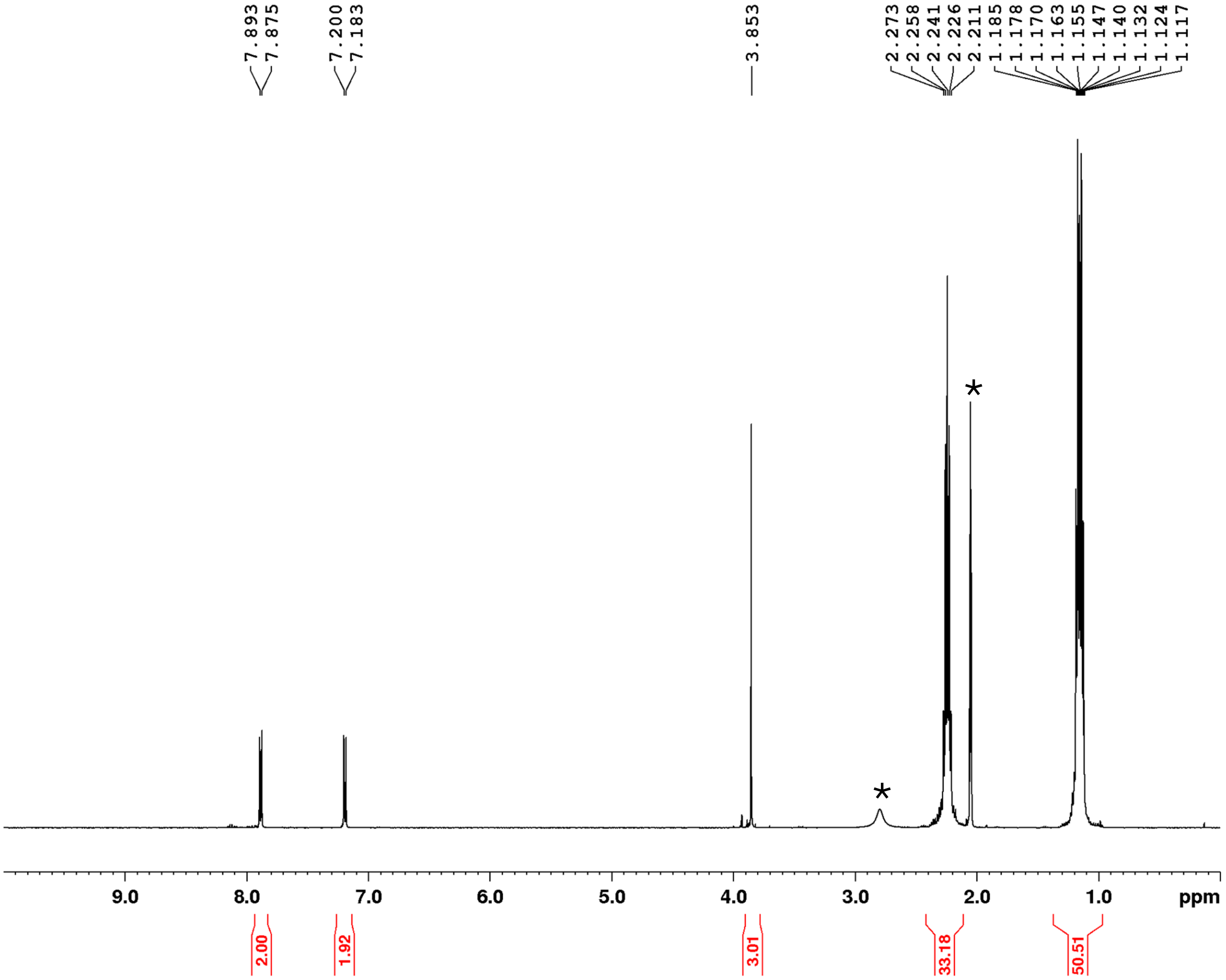

Figure S19. 202.5 MHz $^{31}P\{^{1}H\}$ NMR spectrum of **2** $[Re_6Se_8(PEt_3)_5(C{\equiv}C\text{-}C_6H_4\text{-}CO_2Me)](SbF_6)$ in acetone-d_6.

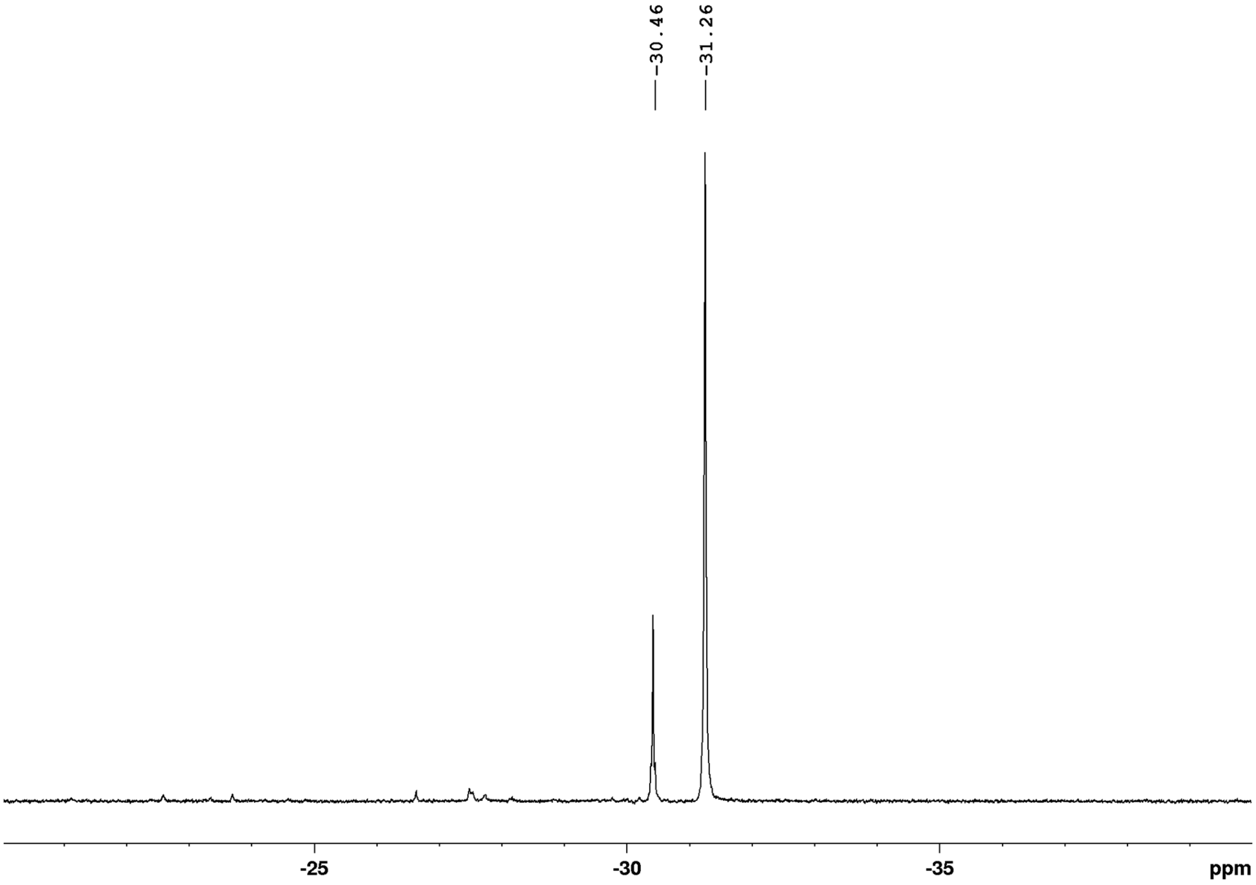

Figure S20. 125 MHz $^{13}C\{^{1}H\}$ NMR spectrum of **2** $[Re_6Se_8(PEt_3)_5(C{\equiv}C\text{-}C_6H_4\text{-}CO_2Me)](SbF_6)$ in acetone-d_6 (solvent peaks are noted with an asterisk). The insets show C_α at 97.3 ppm as well as an expanded region from 120 to 170 ppm.

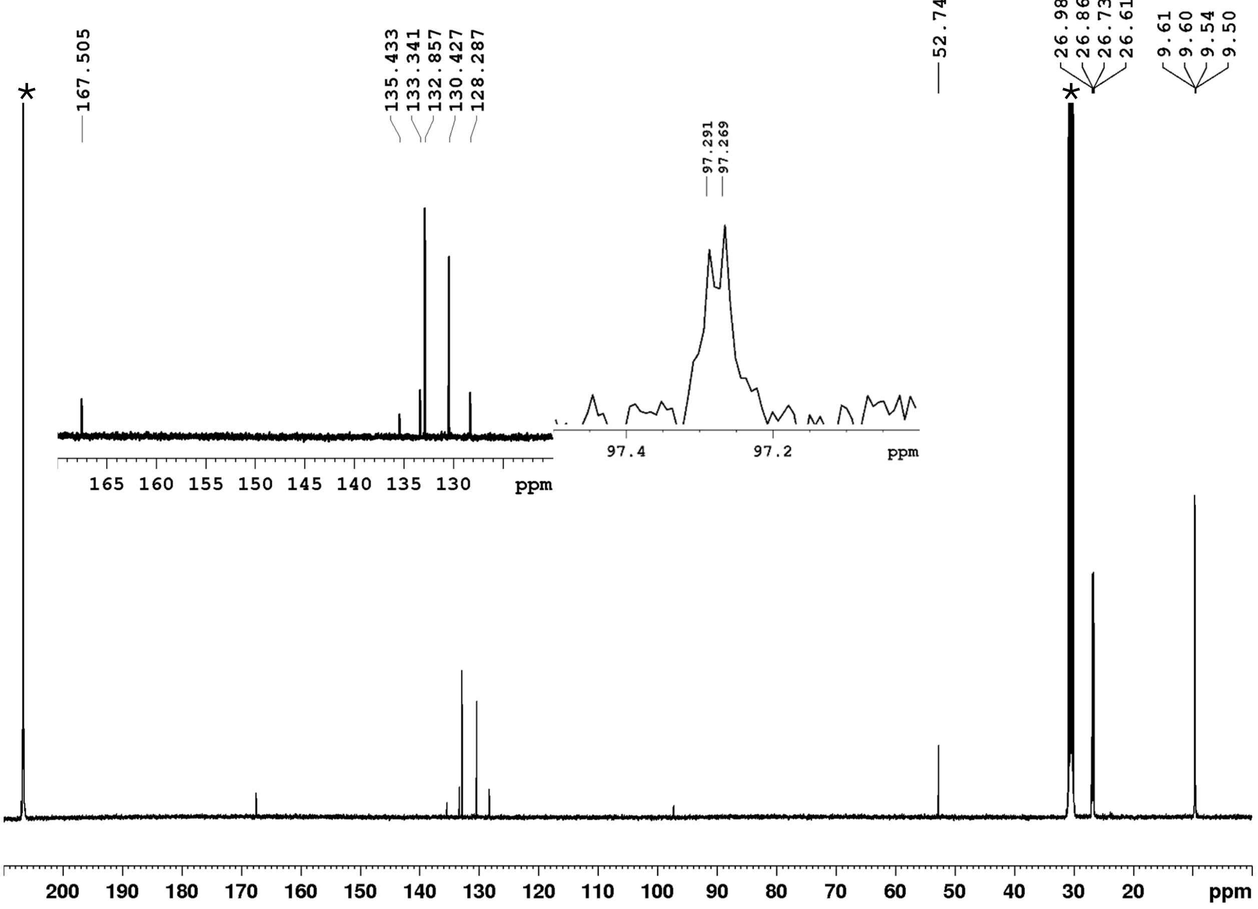

Figure S21. a) Experimental ESI mass spectrometry data and b) theoretical distribution of **2** $[Re_6Se_8(PEt_3)_5(C{\equiv}C\text{-}C_6H_4\text{-}CO_2Me)]^+$.

a)

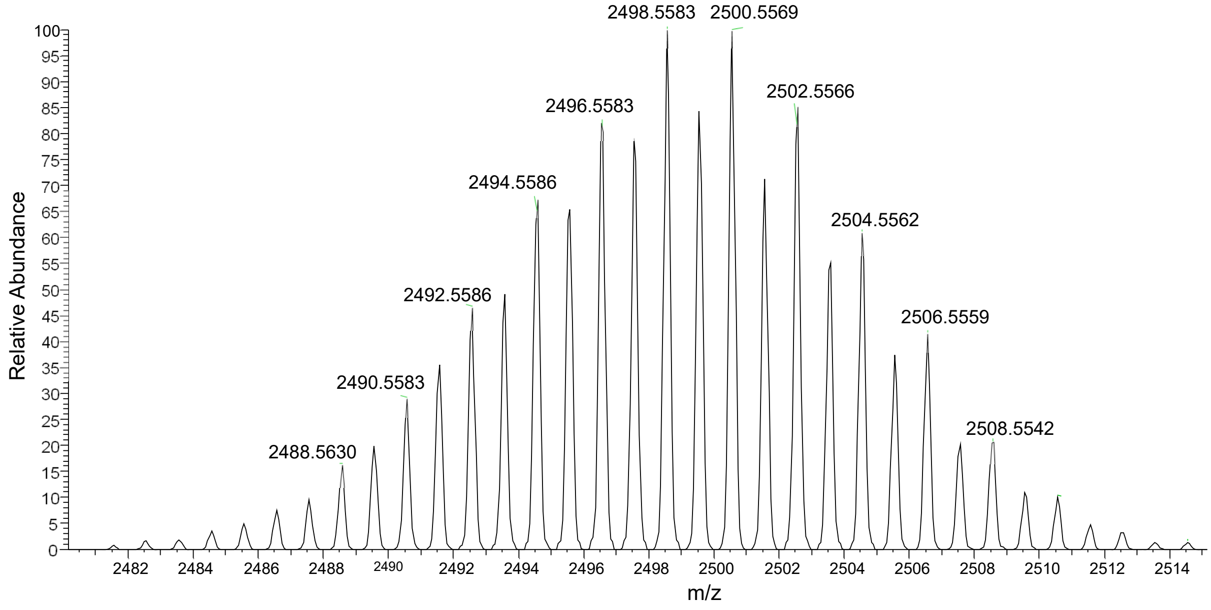

b)

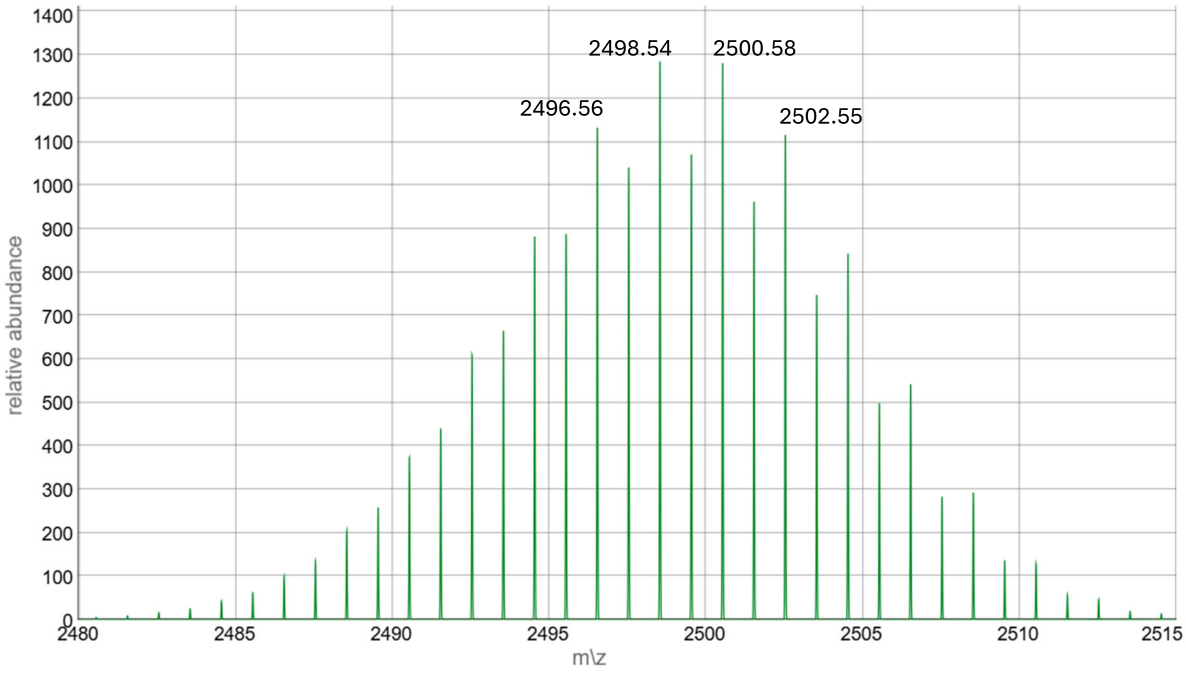

Figure S22. IR-ATR (ZnSe) spectrum of **2** $[Re_6Se_8(PEt_3)_5(C{\equiv}C\text{-}C_6H_4\text{-}CO_2Me)](SbF_6)$.

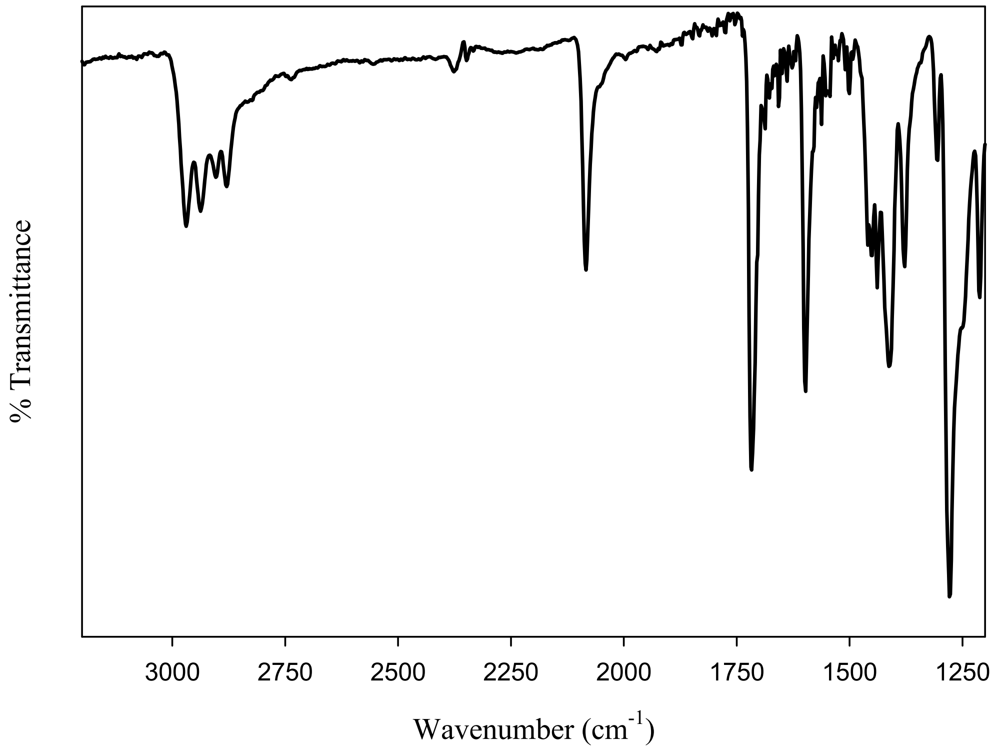

Figure S23. 500 MHz ^{1}H NMR spectrum of **4** $[Re_6Se_8(PEt_3)_5(C{\equiv}C\text{-}C_6H_4\text{-}CH_3)](SbF_6)$ in acetone-d_6 (solvent and water peaks are noted with an asterisk).

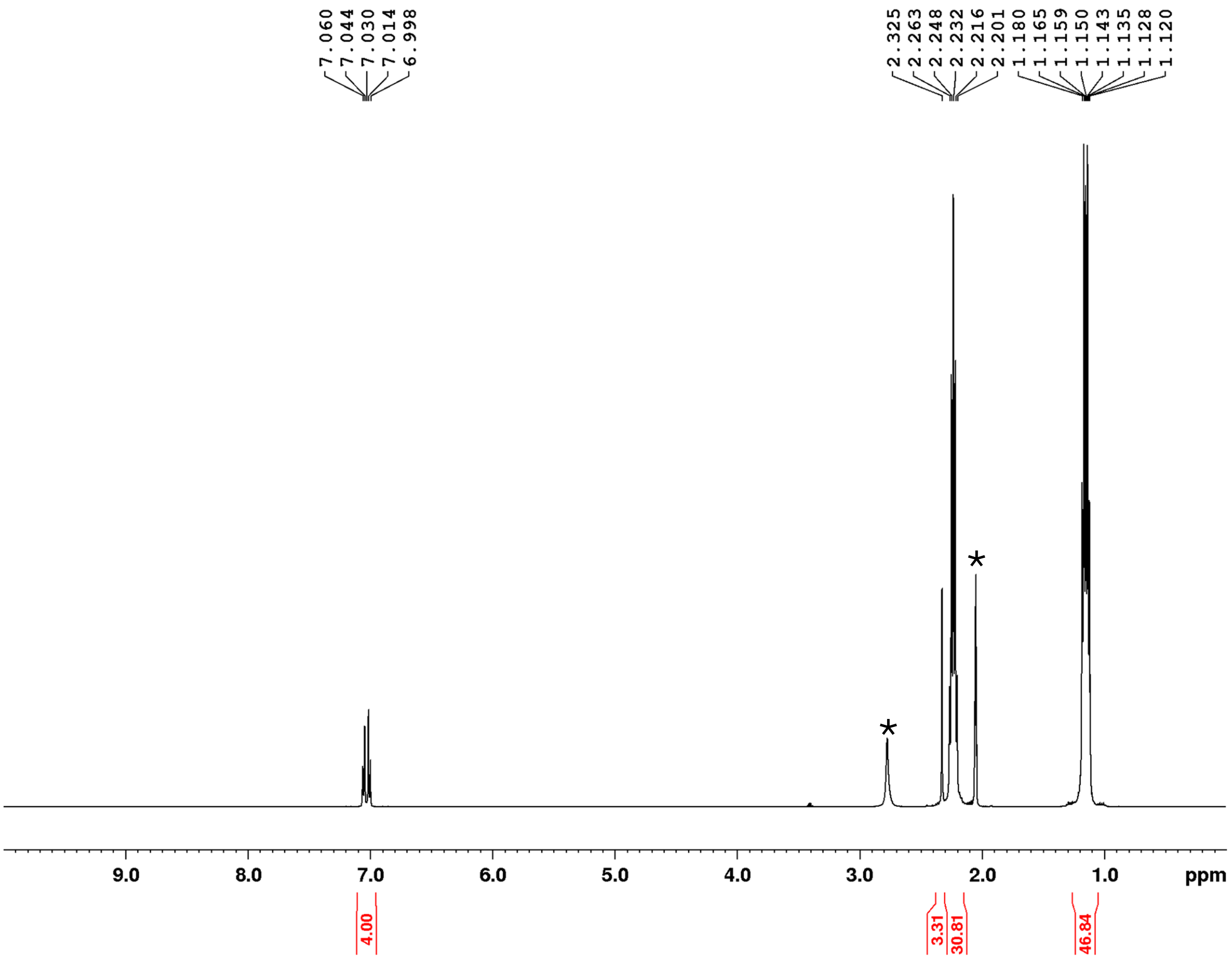

Figure S24. 202.5 MHz $^{31}P\{^{1}H\}$ NMR spectrum of **4** $[Re_6Se_8(PEt_3)_5(C{\equiv}C\text{-}C_6H_4\text{-}CH_3)](SbF_6)$ in acetone-d_6.

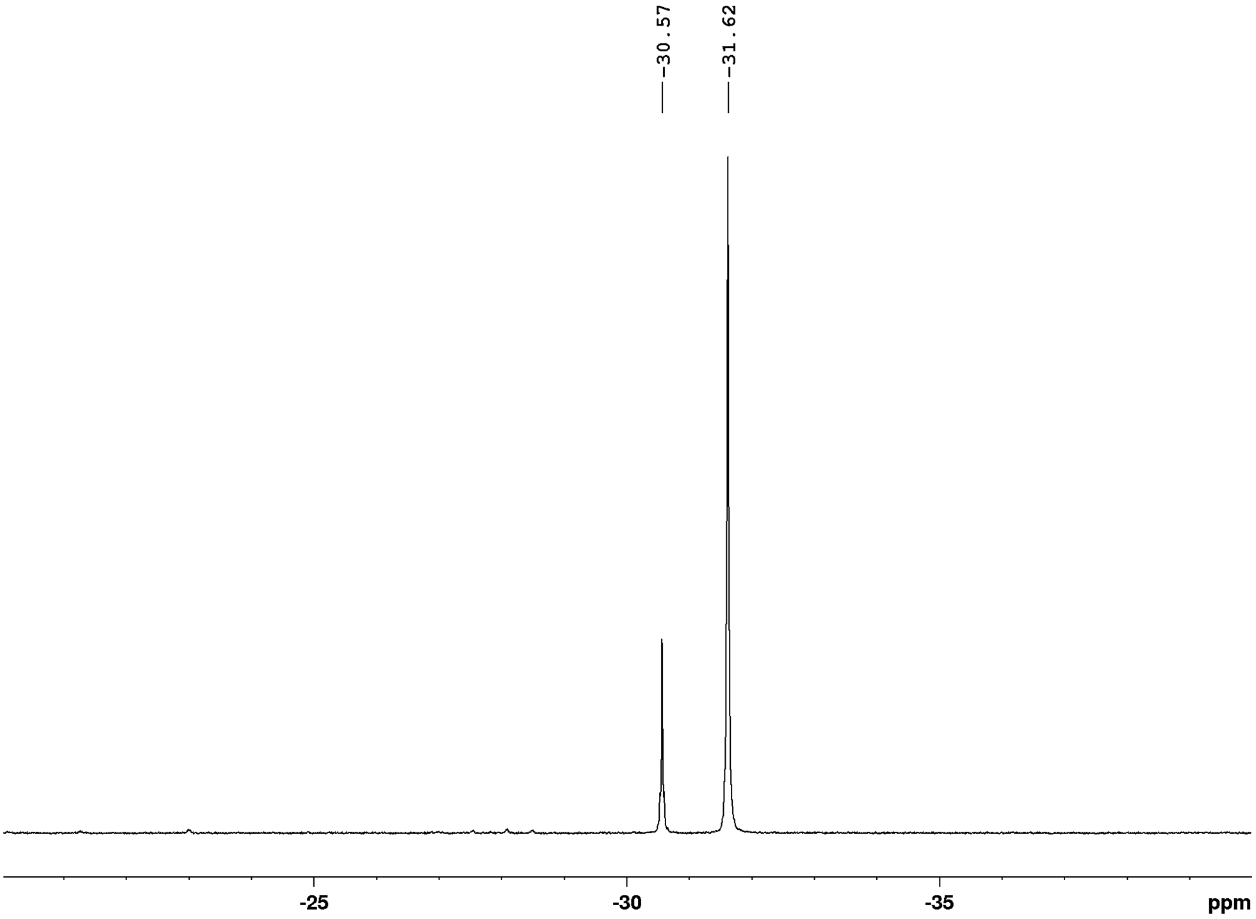

Figure S25. 125 MHz $^{13}C\{^1H\}$ NMR spectrum of **4** [$Re_6Se_8(PEt_3)_5(C{\equiv}C\text{-}C_6H_4\text{-}CH_3)$]($SbF_6$) in acetone-$d_6$ (solvent peaks are noted with an asterisk). The insets show C_α at 88.4 ppm as well as an expanded region around 130 ppm.

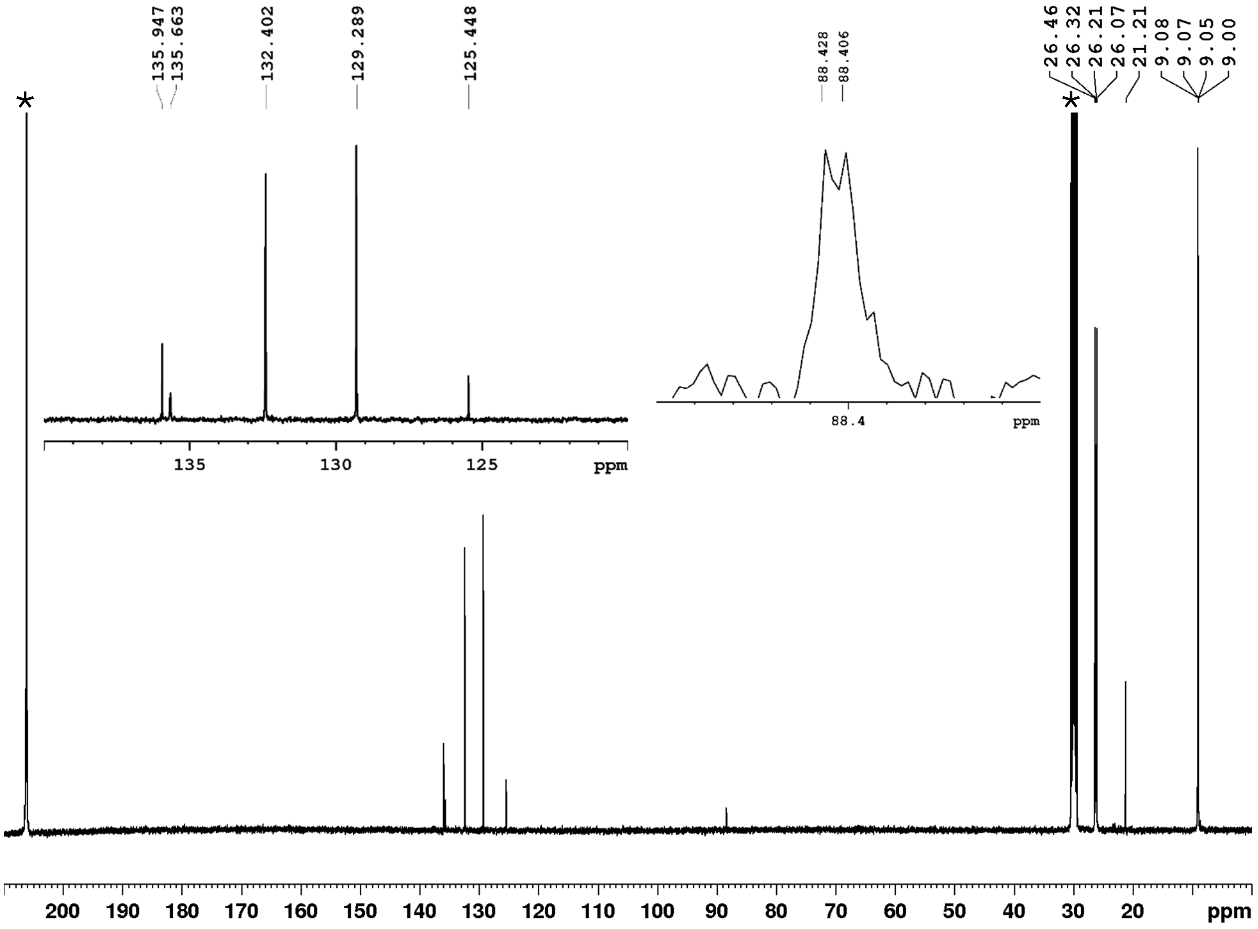

Figure S26. a) Experimental ESI mass spectrometry data and b) theoretical distribution of **4** $[Re_6Se_8(PEt_3)_5(C{\equiv}C\text{-}C_6H_4\text{-}CH_3)]^+$.

a)

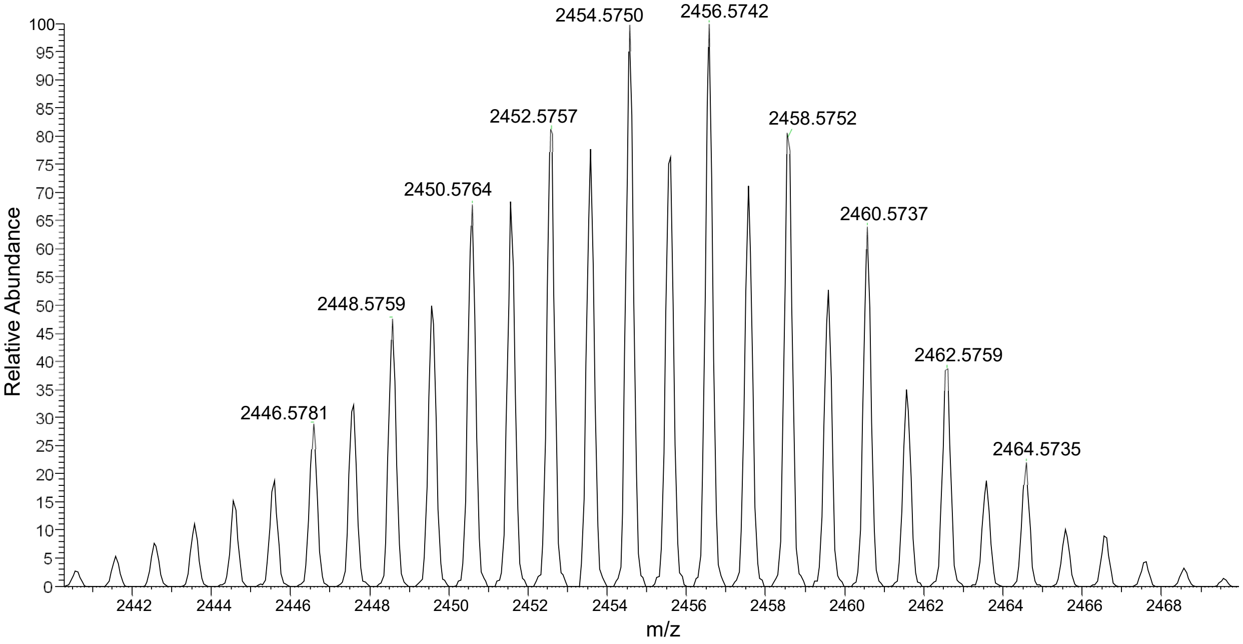

b)

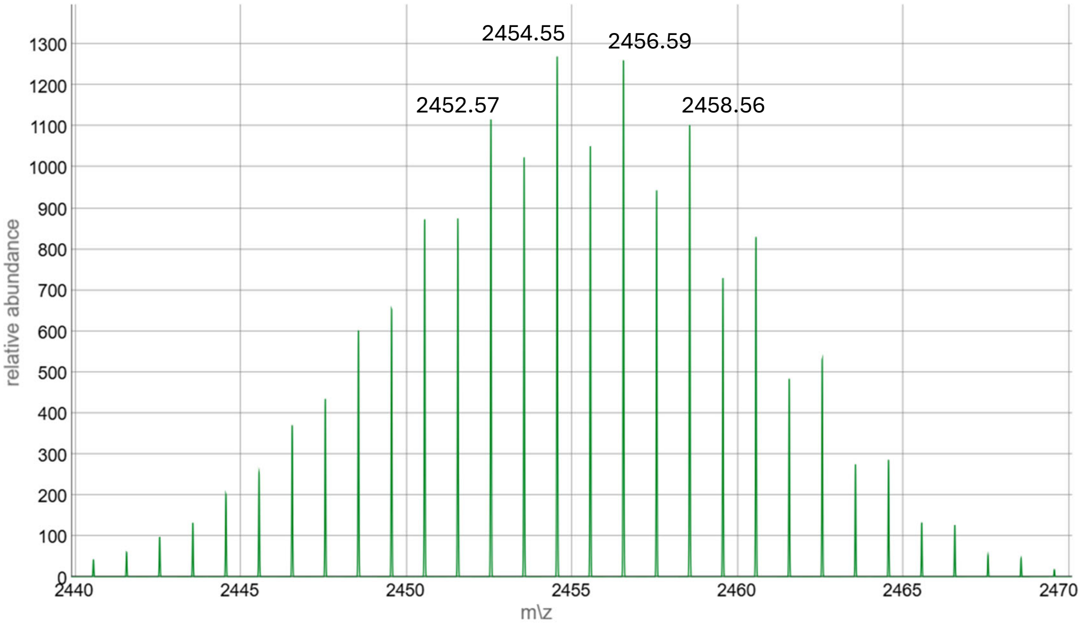

Figure S27. IR-ATR (ZnSe) spectrum of **4** $[Re_6Se_8(PEt_3)_5(C{\equiv}C\text{-}C_6H_4\text{-}CH_3)](SbF_6)$.

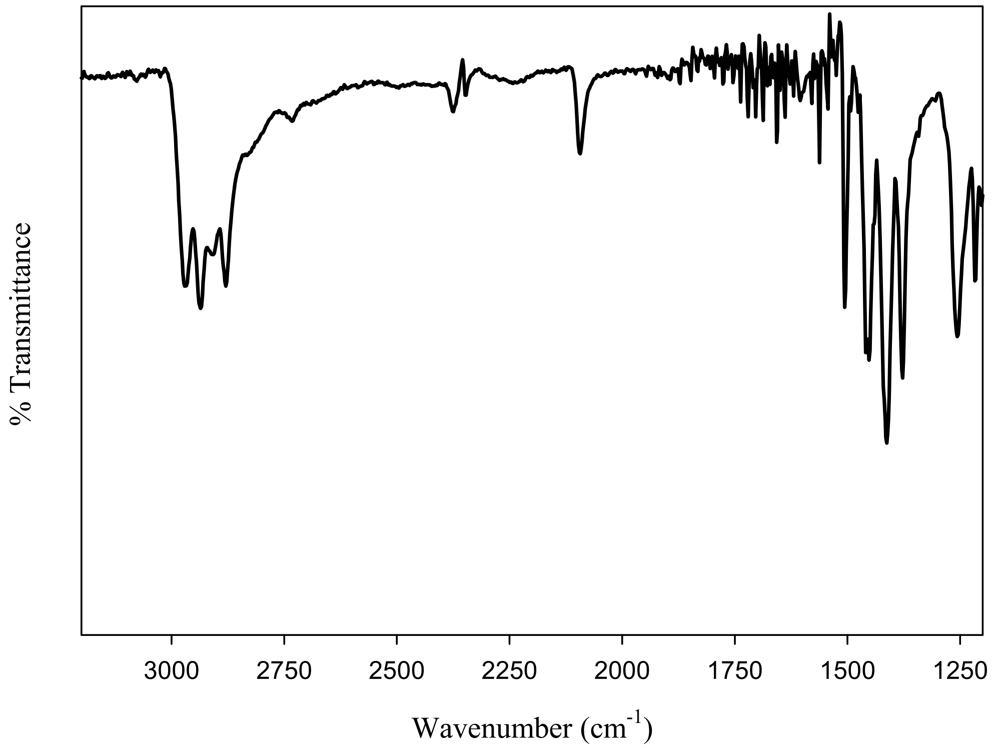

Figure S28. 500 MHz ^{1}H NMR spectrum of **5** $[Re_6Se_8(PEt_3)_5(C{\equiv}C\text{-}C_6H_4\text{-}OMe)](SbF_6)$ in acetone-d_6 (solvent and water peaks are noted with an asterisk).

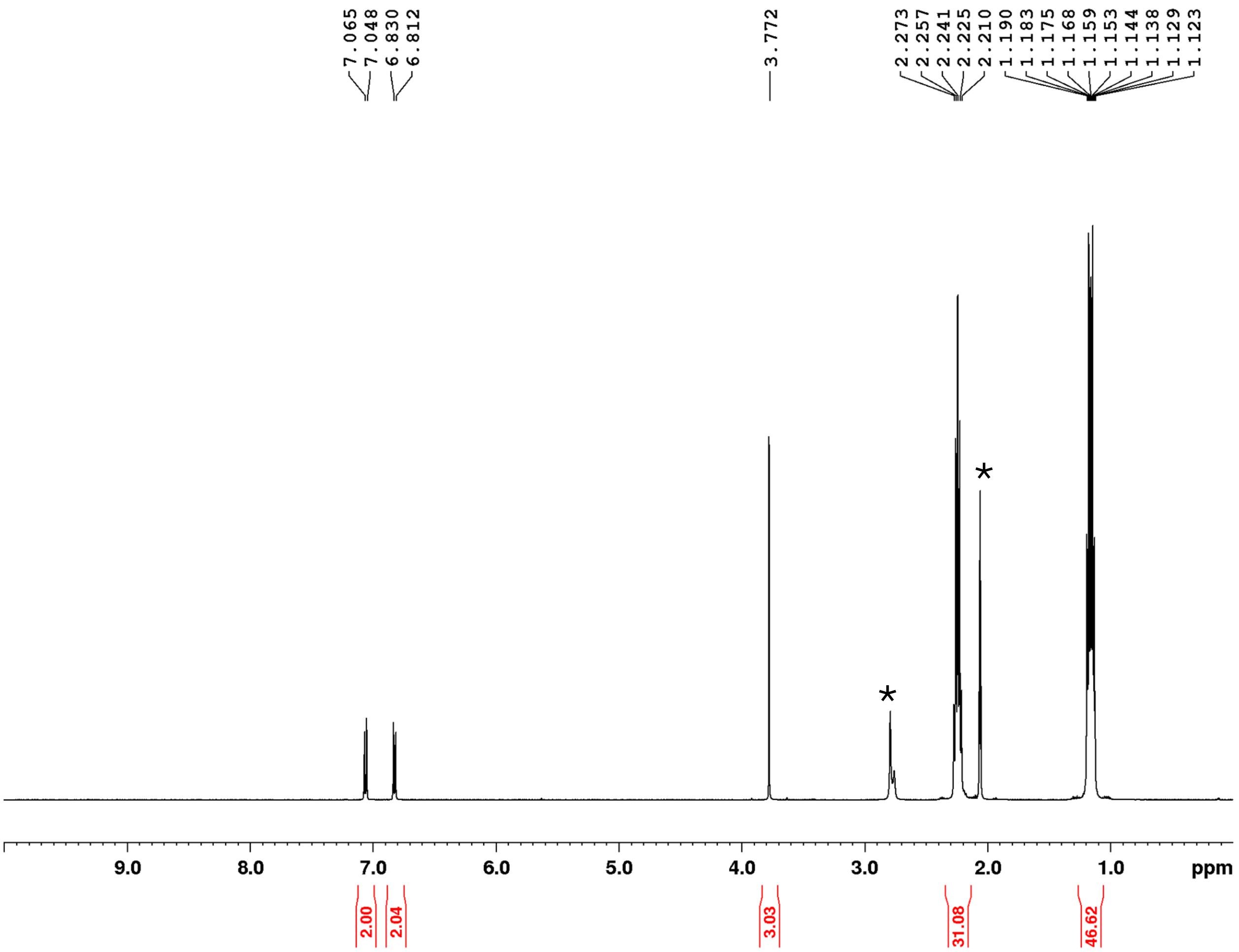

Figure S29. 202.5 MHz $^{31}P\{^1H\}$ NMR spectrum of **5** $[Re_6Se_8(PEt_3)_5(C{\equiv}C\text{-}C_6H_4\text{-}OMe)](SbF_6)$ in acetone-d_6.

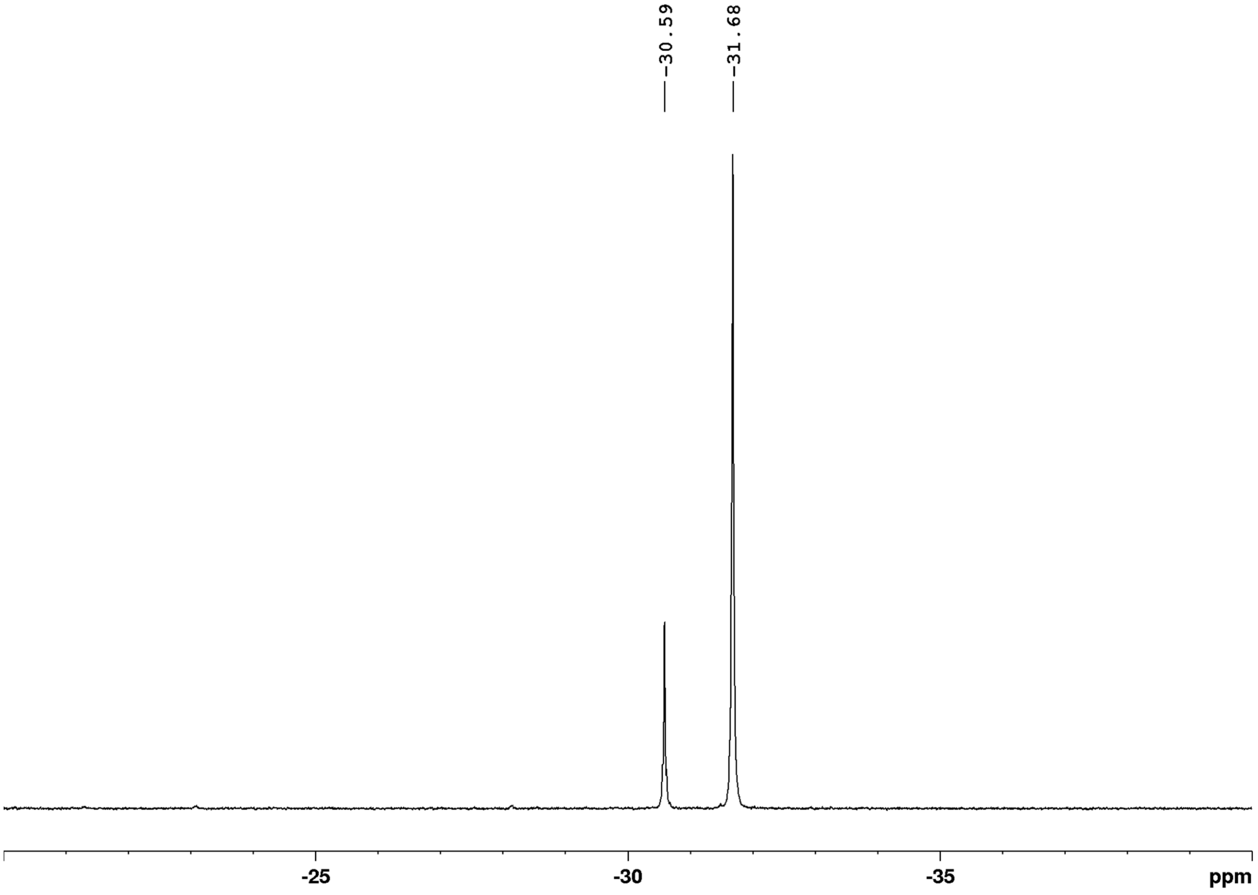

Figure S30. 125 MHz $^{13}C\{^{1}H\}$ NMR spectrum of **5** $[Re_6Se_8(PEt_3)_5(C{\equiv}C\text{-}C_6H_4\text{-}OMe)](SbF_6)$ in acetone-d_6 (solvent peaks are noted with an asterisk). The insets show C_α at 87.3 ppm as well as an expanded region around 140 ppm.

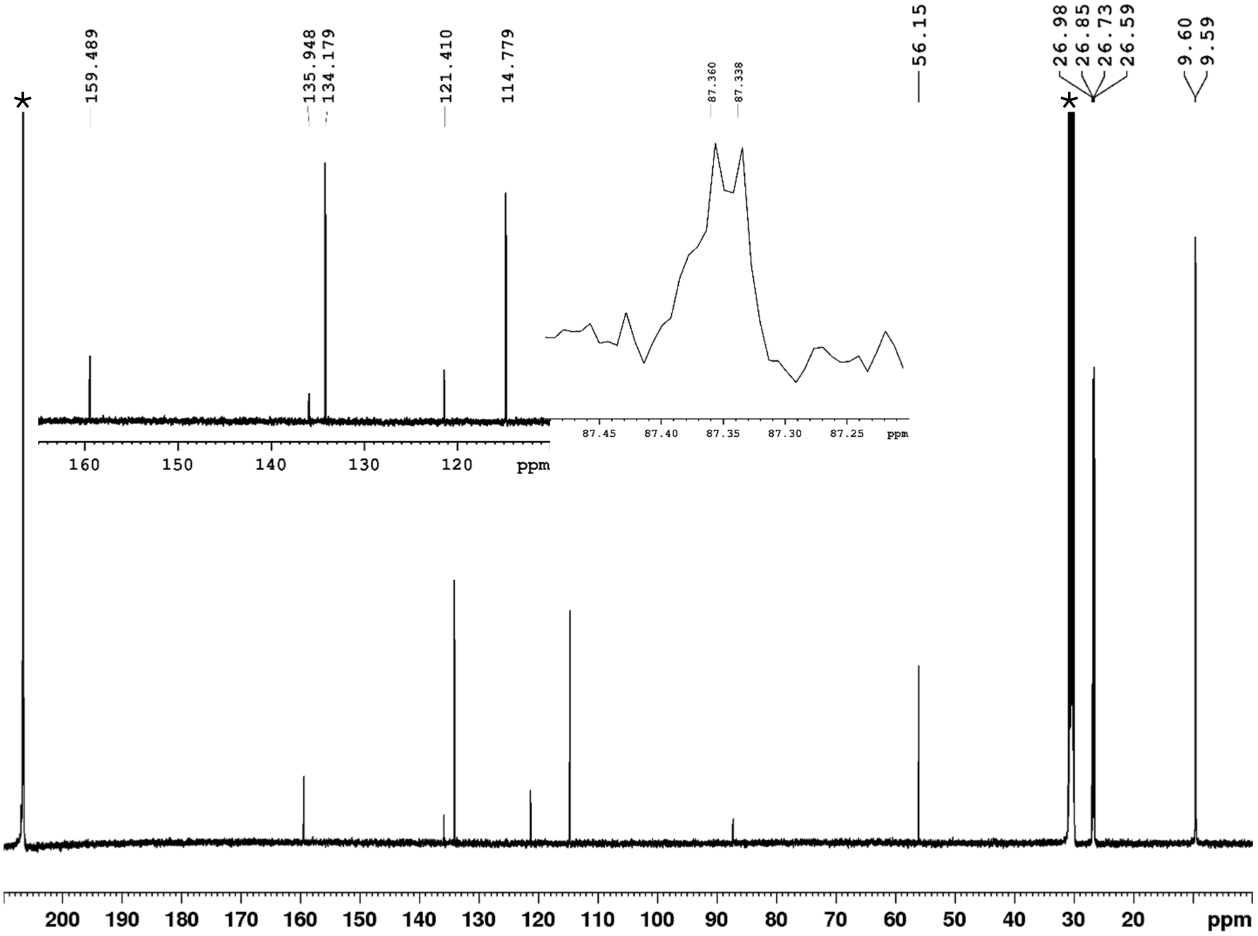

Figure S31. a) Experimental ESI mass spectrometry data and b) theoretical distribution of **5** $[Re_6Se_8(PEt_3)_5(C{\equiv}C\text{-}C_6H_4\text{-}OMe)]^+$.

a)

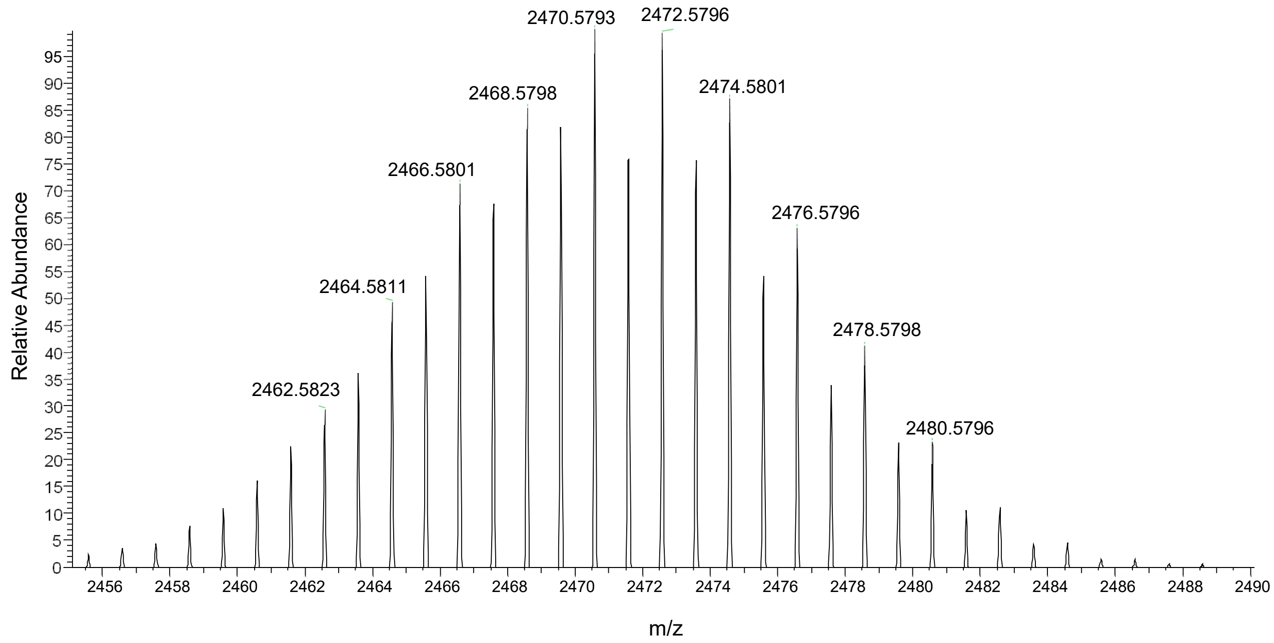

b)

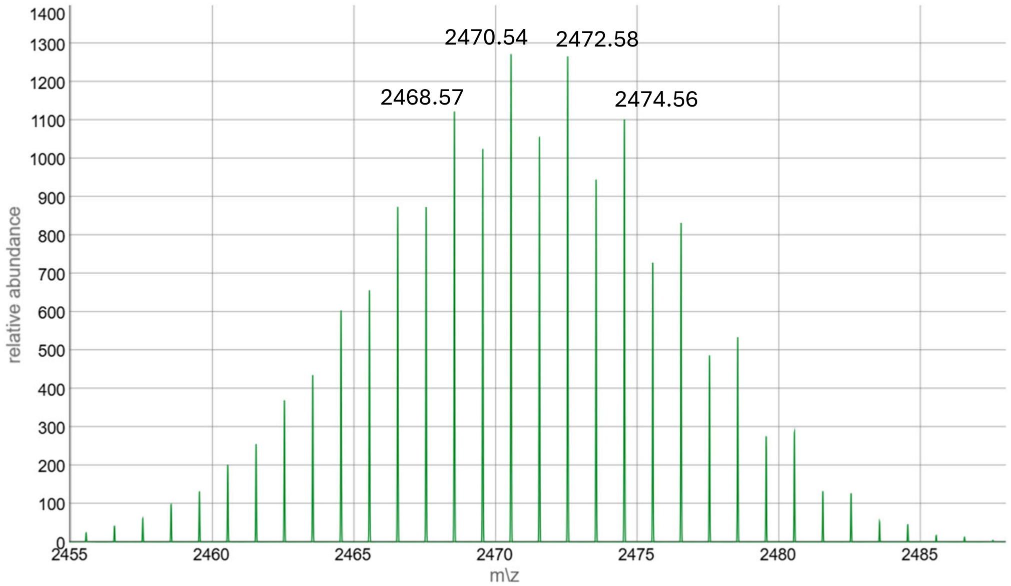

Figure S32. IR-ATR (ZnSe) spectrum of **5** [$Re_6Se_8(PEt_3)_5(C{\equiv}C\text{-}C_6H_4\text{-}OMe)$]($SbF_6$).

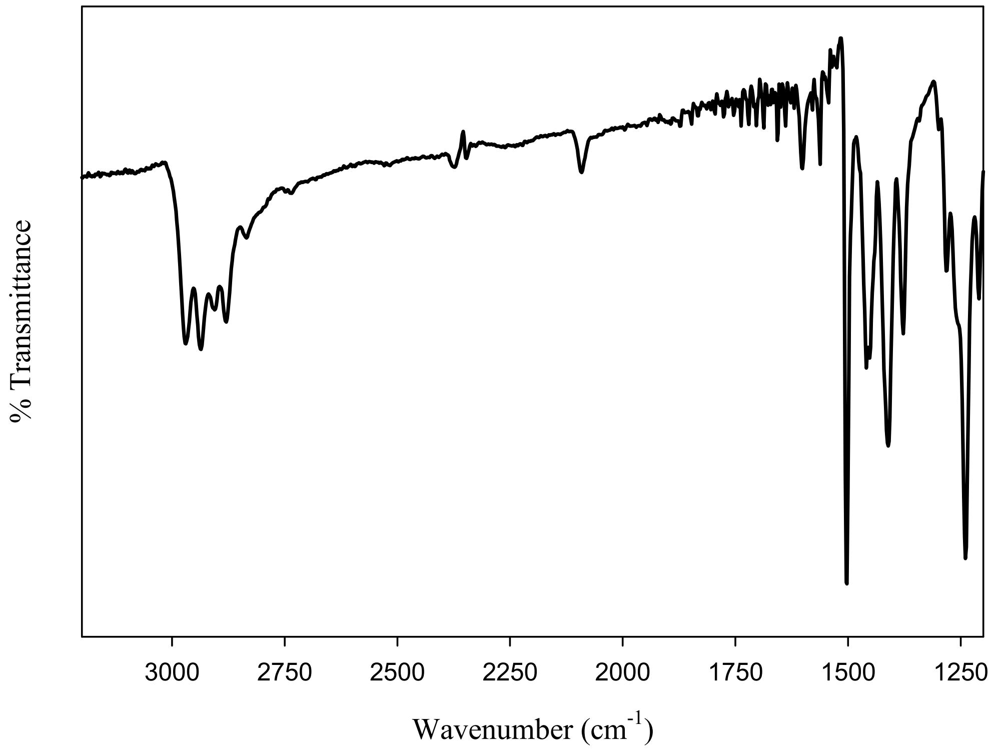

Emission data for 1-7

Figure S33. a) Excitation (solid line, λ_{obs} = 715 nm) and emission (dashed line, λ_{ex} = 375 nm) spectra and b) the corresponding emission vs. excitation map of **1** $[Re_6Se_8(PEt_3)_5(C{\equiv}C\text{-}C_6H_4\text{-}NO_2)](SbF_6)$ in deaerated CH_3CN.

a) b)

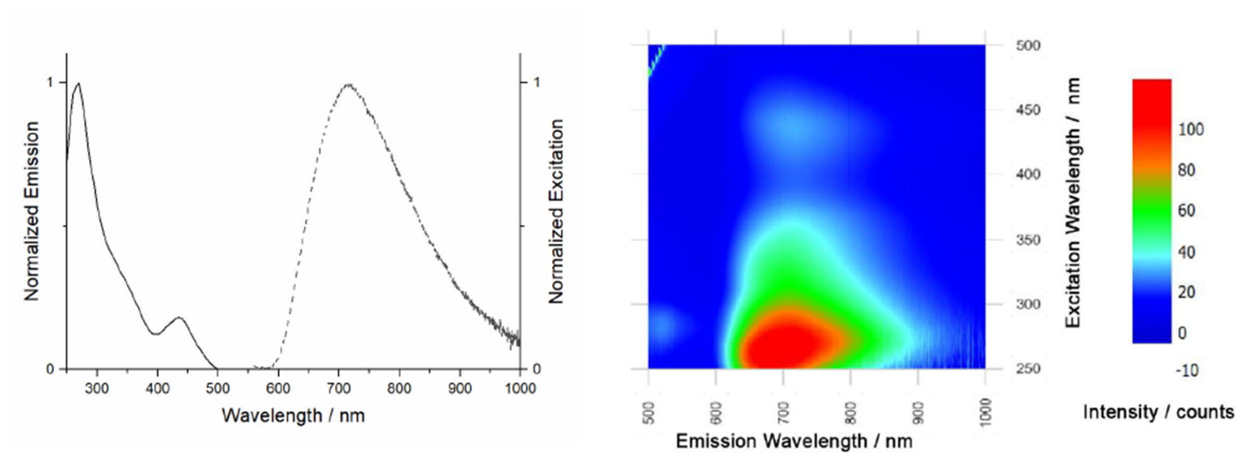

Figure S34. a) Excitation (solid line, λ_{obs} = 740 nm) and emission (dashed line, λ_{ex} = 410 nm) spectra and b) the corresponding emission vs. excitation map of **1** $[Re_6Se_8(PEt_3)_5(C{\equiv}C\text{-}C_6H_4\text{-}NO_2)](SbF_6)$ in powder phase.

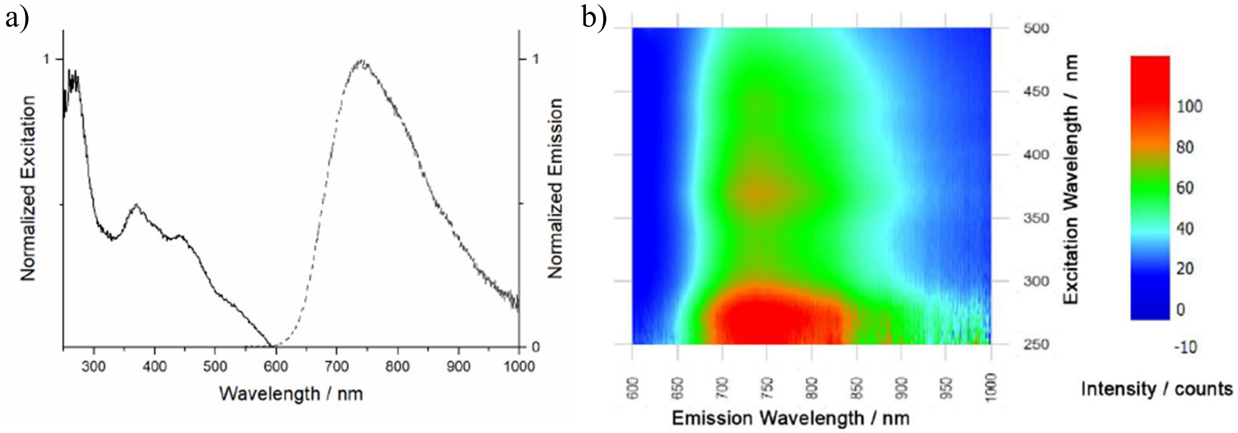

Figure S35. Integrated emission decay profile of **1** $[Re_6Se_8(PEt_3)_5(C{\equiv}C\text{-}C_6H_4\text{-}NO_2)](SbF_6)$ in a) deaerated CH_3CN and b) powder phase.

a)

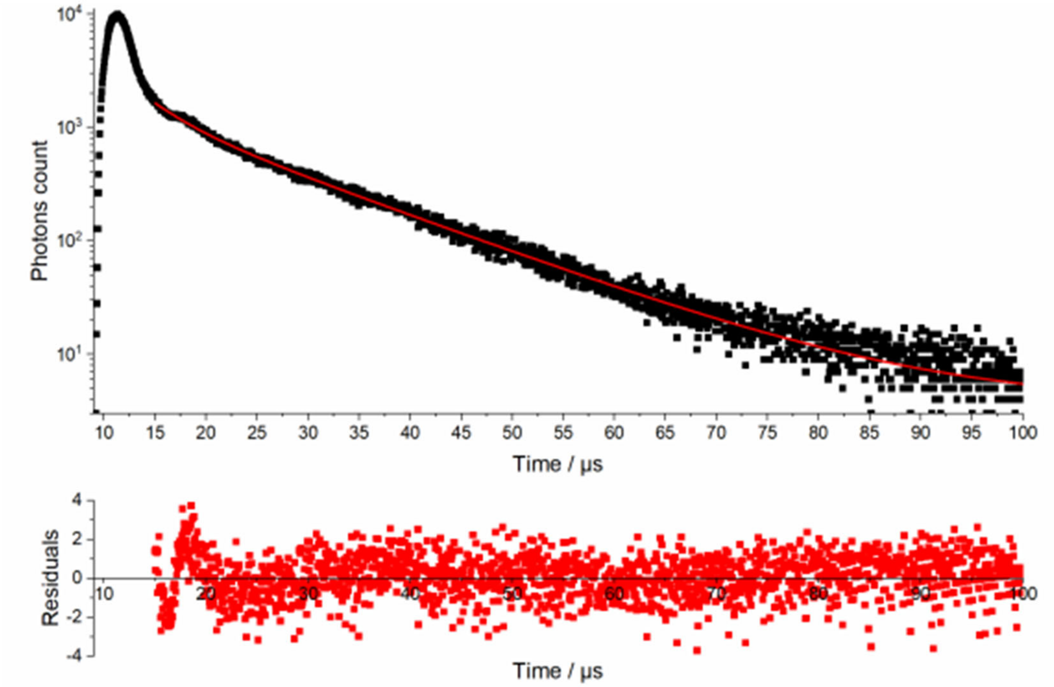

b)

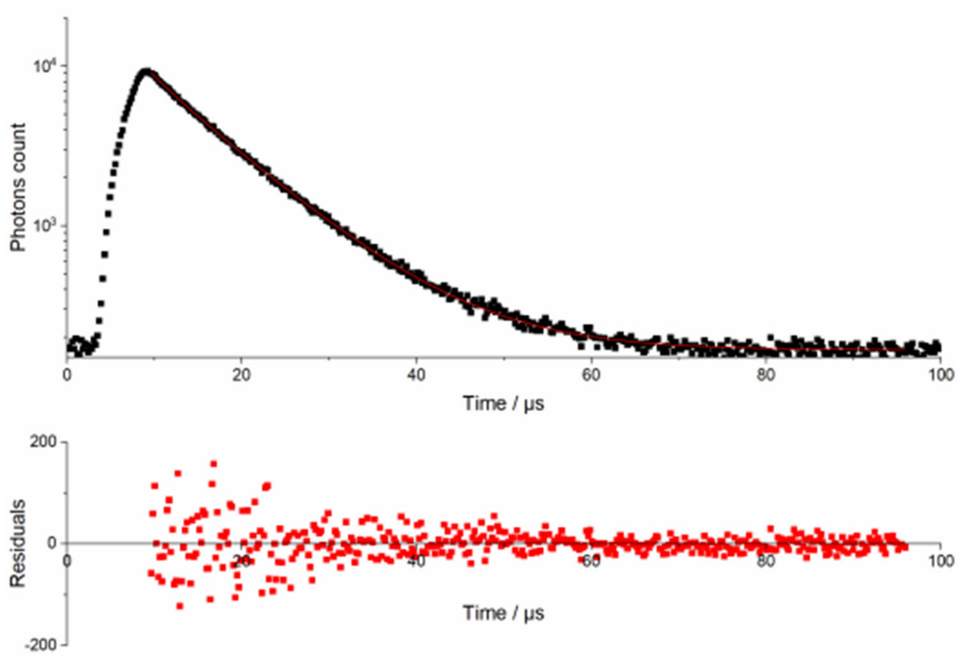

Figure S36. a) Excitation (plain line, λ_{obs} = 760 nm) and emission (dashed line, λ_{ex} = 420 nm) spectra and b) the corresponding emission vs. excitation map of **2** [$Re_6Se_8(PEt_3)_5(C{\equiv}C\text{-}C_6H_4\text{-}CO_2Me)$]($SbF_6$) in deaerated CH_3CN.

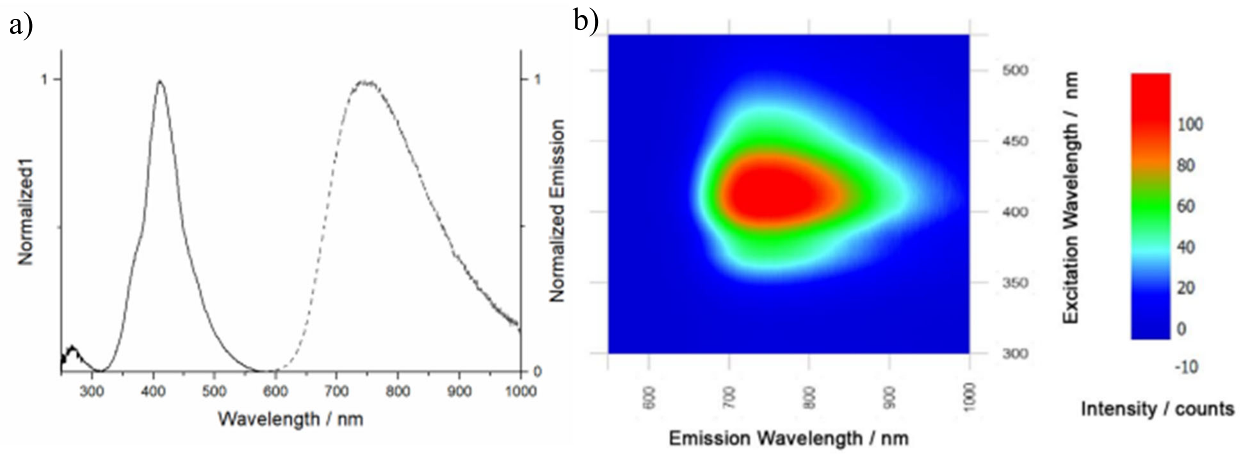

Figure S37. a) Excitation (plain line, λ_{obs} = 760 nm) and emission (dashed line, λ_{ex} = 375 nm) spectra and b) the corresponding emission vs. excitation map of **2** [$Re_6Se_8(PEt_3)_5(C{\equiv}C\text{-}C_6H_4\text{-}CO_2Me)$]($SbF_6$) in powder phase.

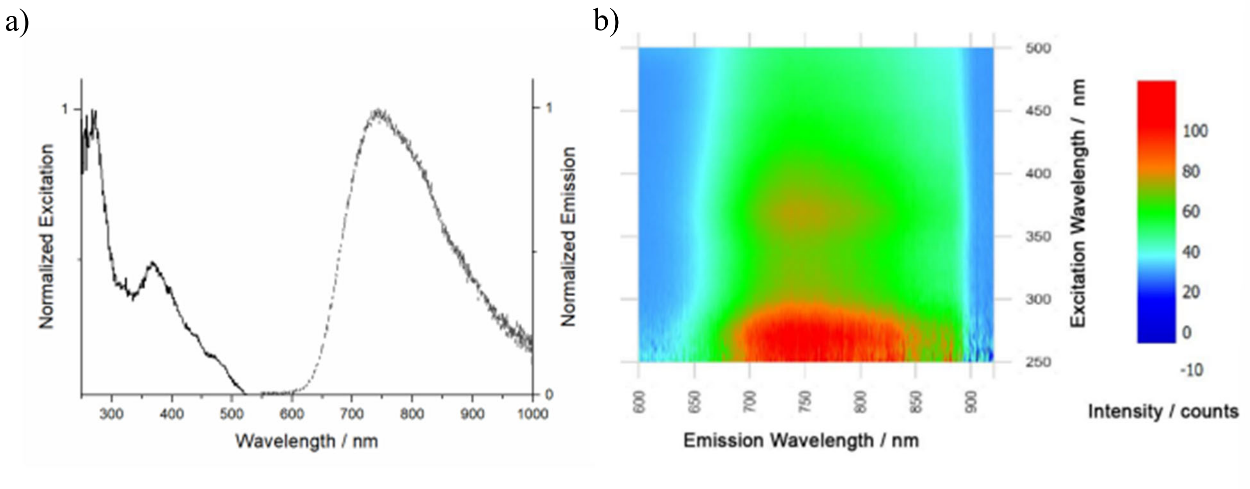

Figure S38. Integrated emission decay profile of **2** [$Re_6Se_8(PEt_3)_5(C{\equiv}C\text{-}C_6H_4\text{-}CO_2Me)$]($SbF_6$) in a) deaerated CH_3CN and b) powder phase.

a)

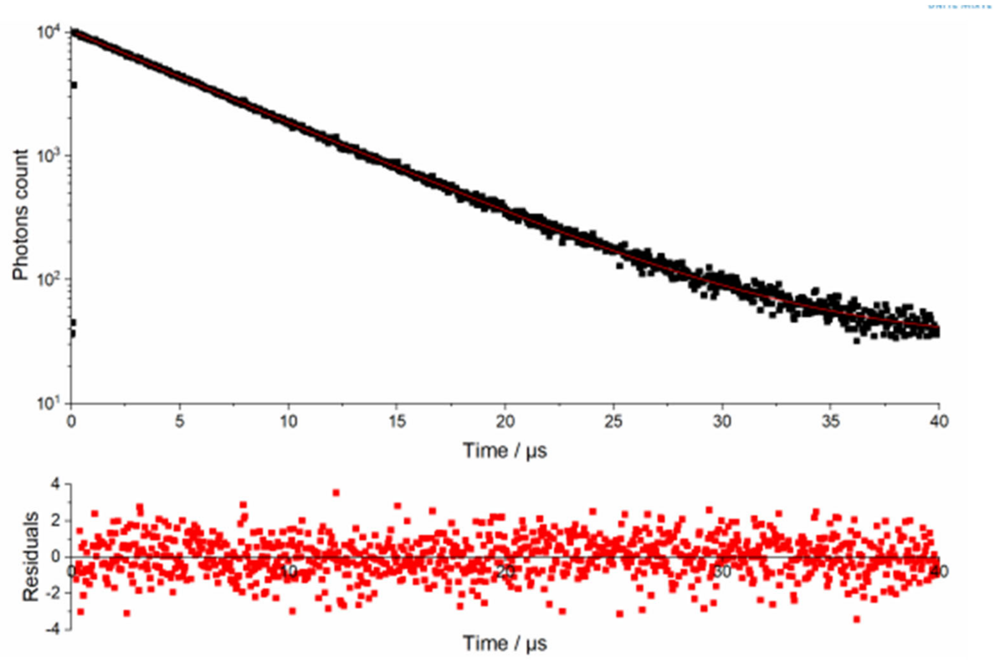

b)

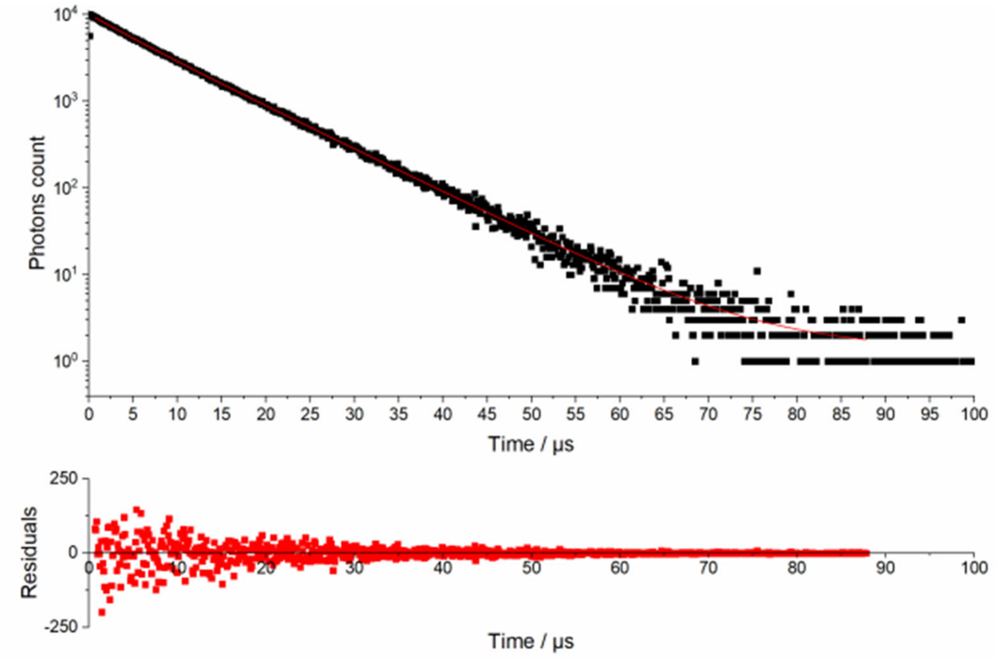

Figure S39. a) Excitation (plain line, λ_{obs} = 750 nm) and emission (dashed line, λ_{ex} = 410 nm) spectra and b) the corresponding emission vs. excitation map of **3** $[Re_6Se_8(PEt_3)_5(C{\equiv}C\text{-}C_6H_5)](SbF_6)$ in deaerated CH_3CN.

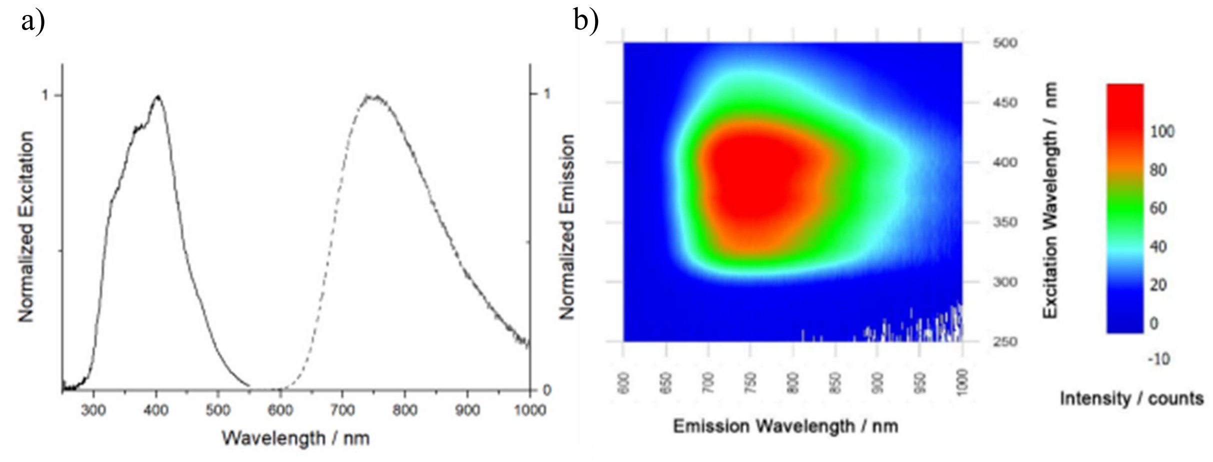

Figure S40. a) Excitation (plain line, λ_{obs} = 750 nm) and emission (dashed line, λ_{ex} = 410 nm) spectra and b) the corresponding emission vs. excitation map of **3** $[Re_6Se_8(PEt_3)_5(C{\equiv}C\text{-}C_6H_5)](SbF_6)$ in powder phase.

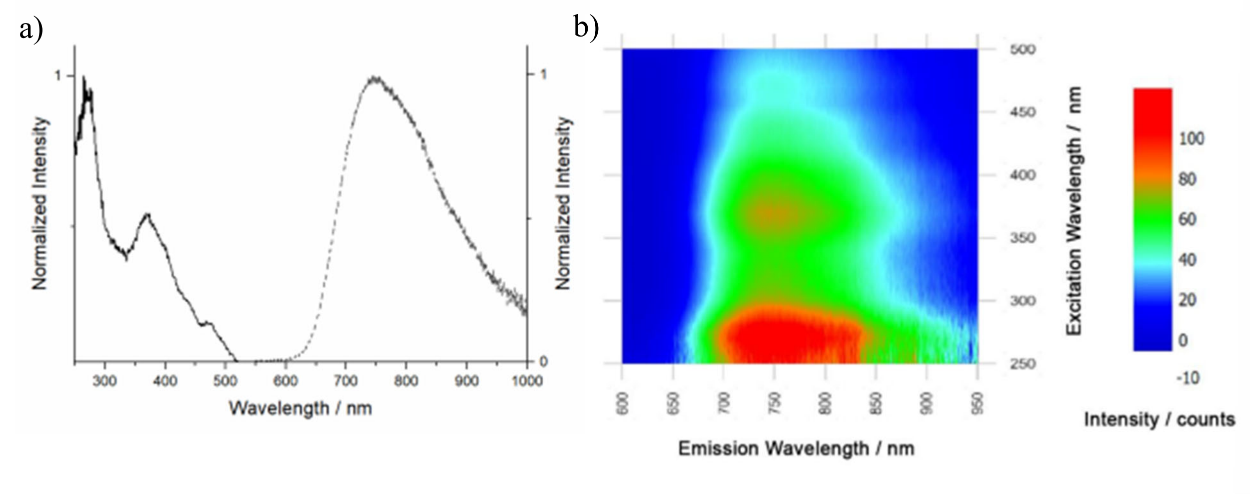

Figure S41. Integrated emission decay profile of **3** [$Re_6Se_8(PEt_3)_5(C\equiv C\text{-}C_6H_5)$]($SbF_6$) in a) deaerated CH_3CN and b) powder phase.

a)

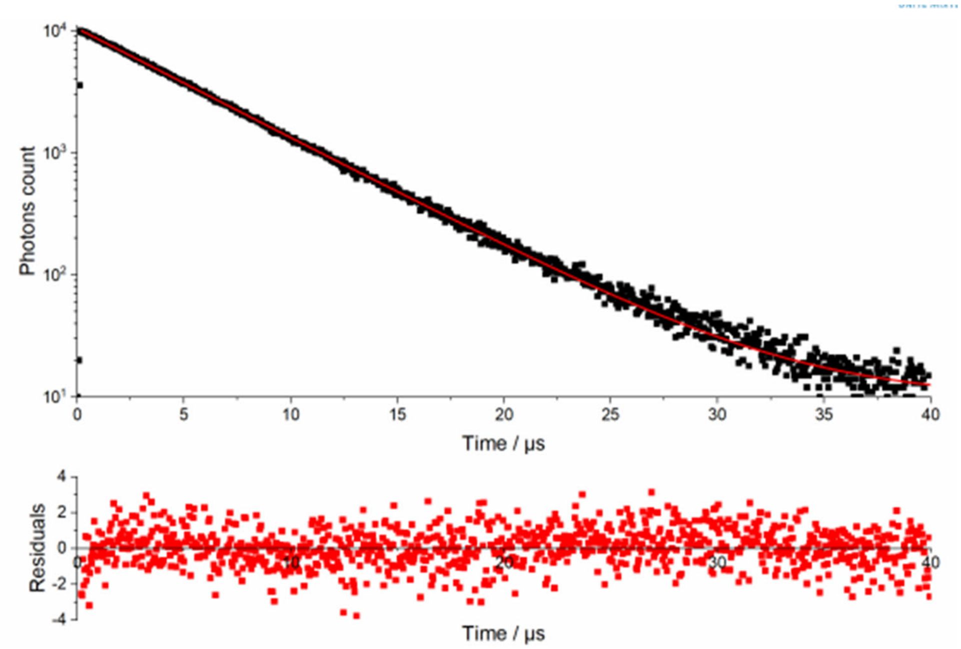

b)

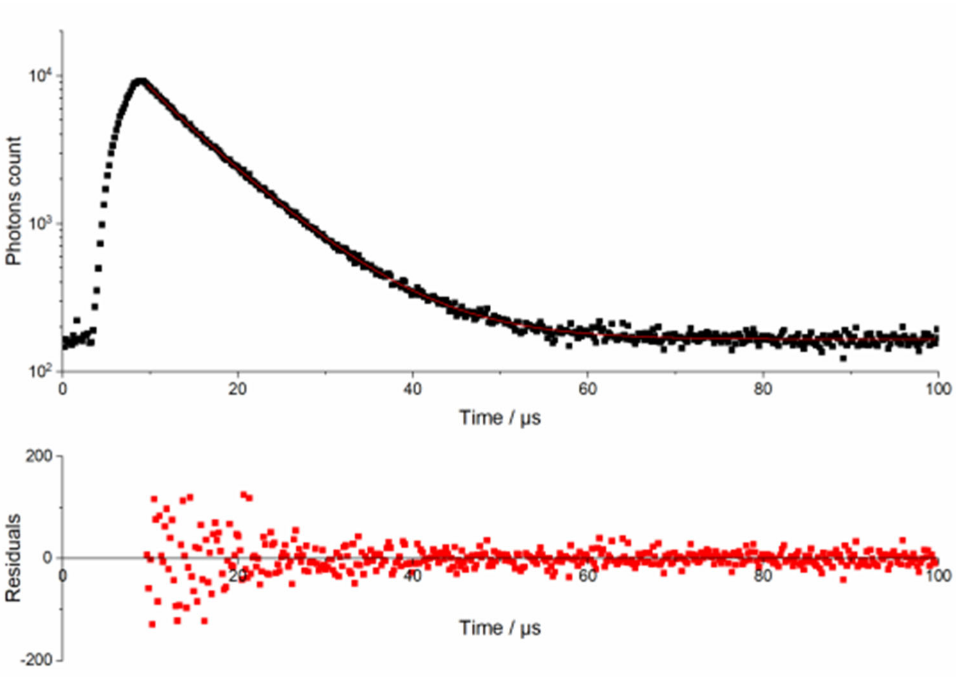

Figure S42. a) Excitation (plain line, λ_{obs} = 760 nm) and emission (dashed line, λ_{ex} = 420 nm) spectra and b) the corresponding emission vs. excitation map of **4** $[Re_6Se_8(PEt_3)_5(C{\equiv}C\text{-}C_6H_4\text{-}CH_3)](SbF_6)$ in deaerated CH_3CN.

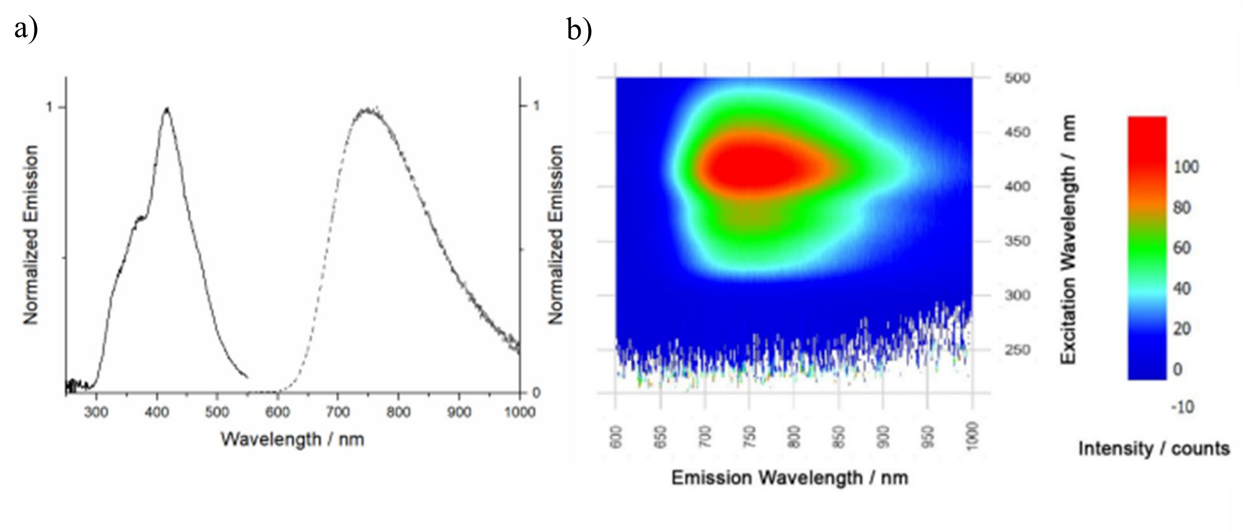

Figure S43. a) Excitation (plain line, λ_{obs} = 760 nm) and emission (dashed line, λ_{ex} = 420 nm) spectra and b) the corresponding emission vs. excitation map of **4** $[Re_6Se_8(PEt_3)_5(C{\equiv}C\text{-}C_6H_4\text{-}CH_3)](SbF_6)$ in powder phase.

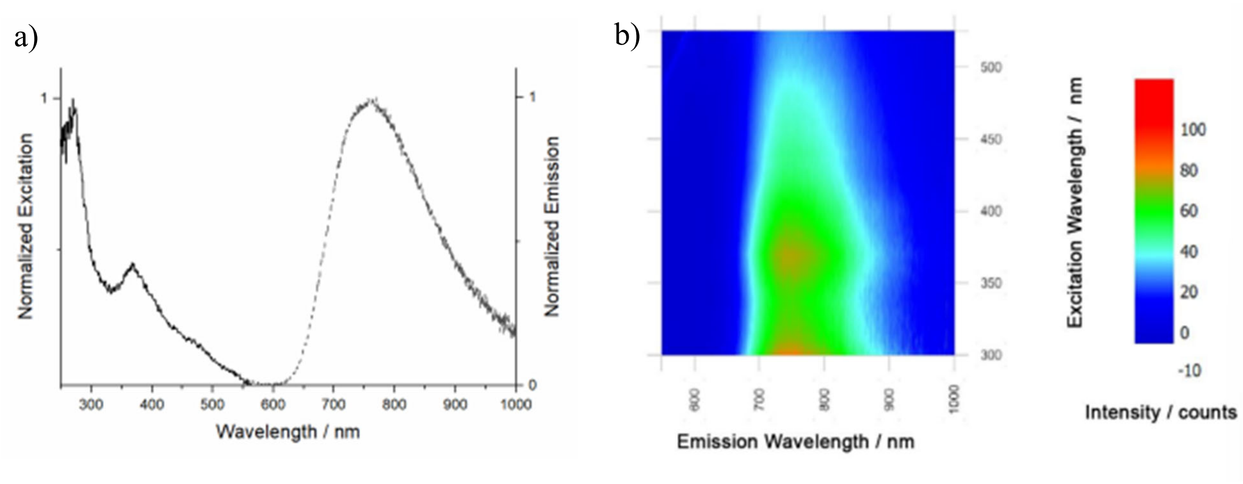

Figure S44. Integrated emission decay profile of **4** $[Re_6Se_8(PEt_3)_5(C{\equiv}C\text{-}C_6H_4\text{-}CH_3)](SbF_6)$ in a) deaerated CH_3CN and b) powder phase.

a)

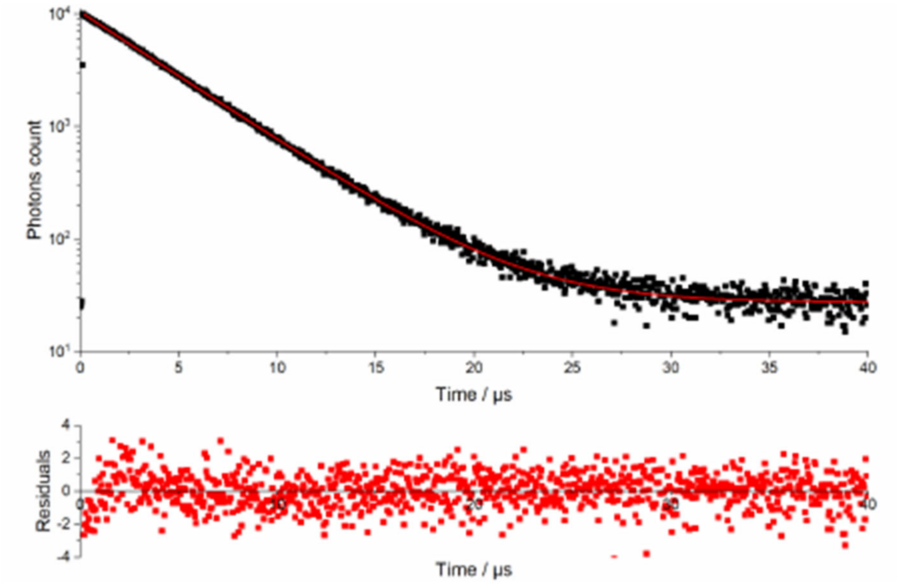

b)

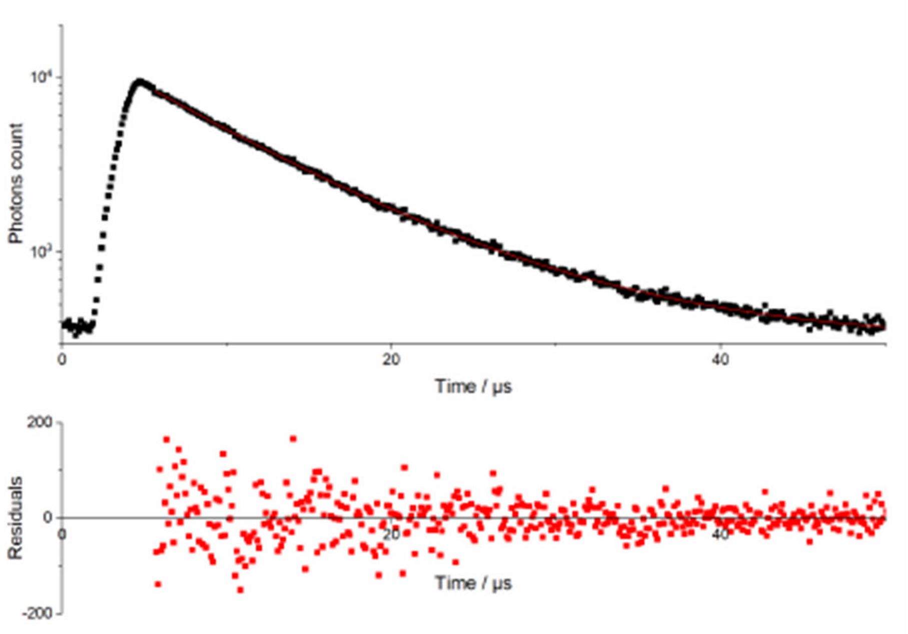

Figure S45. a) Excitation (plain line, λ_{obs} = 760 nm) and emission (dashed line, λ_{ex} = 420 nm) spectra and b) the corresponding emission vs. excitation map of **5** $[Re_6Se_8(PEt_3)_5(C{\equiv}C\text{-}C_6H_4\text{-}OMe)](SbF_6)$ in deaerated CH_3CN.

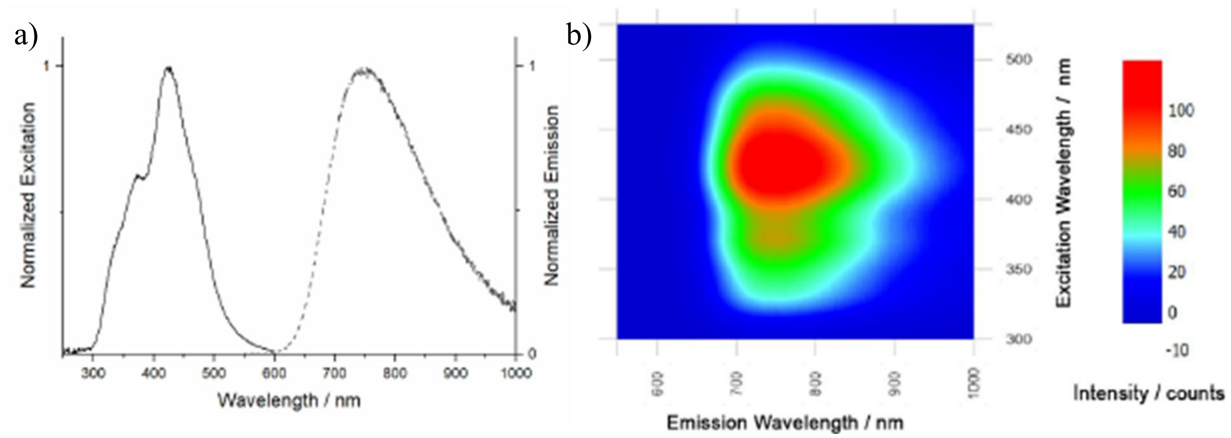

Figure S46. a) Excitation (plain line, λ_{obs} = 760 nm) and emission (dashed line, λ_{ex} = 420 nm) spectra and b) the corresponding emission vs. excitation map of **5** $[Re_6Se_8(PEt_3)_5(C{\equiv}C\text{-}C_6H_4\text{-}OMe)](SbF_6)$ in powder phase.

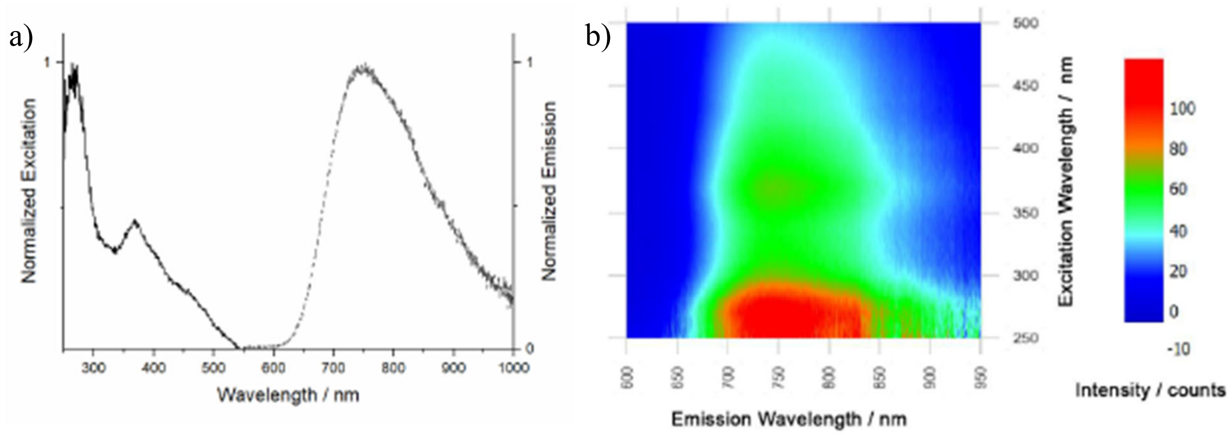

Figure S47. Integrated emission decay profile of **5** $[Re_6Se_8(PEt_3)_5(C{\equiv}C\text{-}C_6H_4\text{-}OMe)](SbF_6)$ in a) deaerated CH_3CN and b) powder phase.

a)

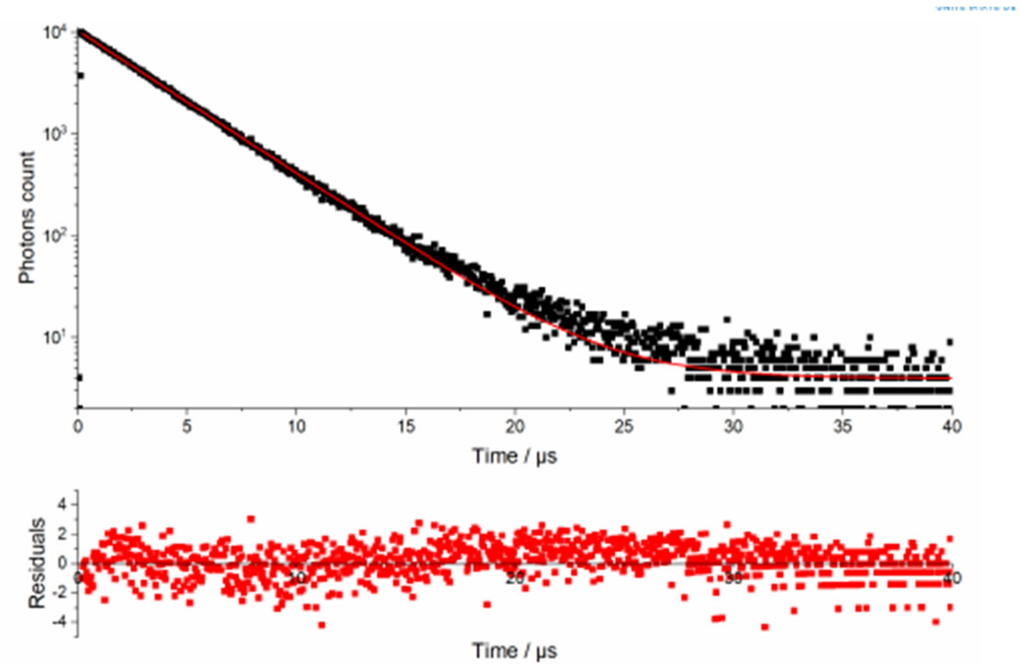

b)

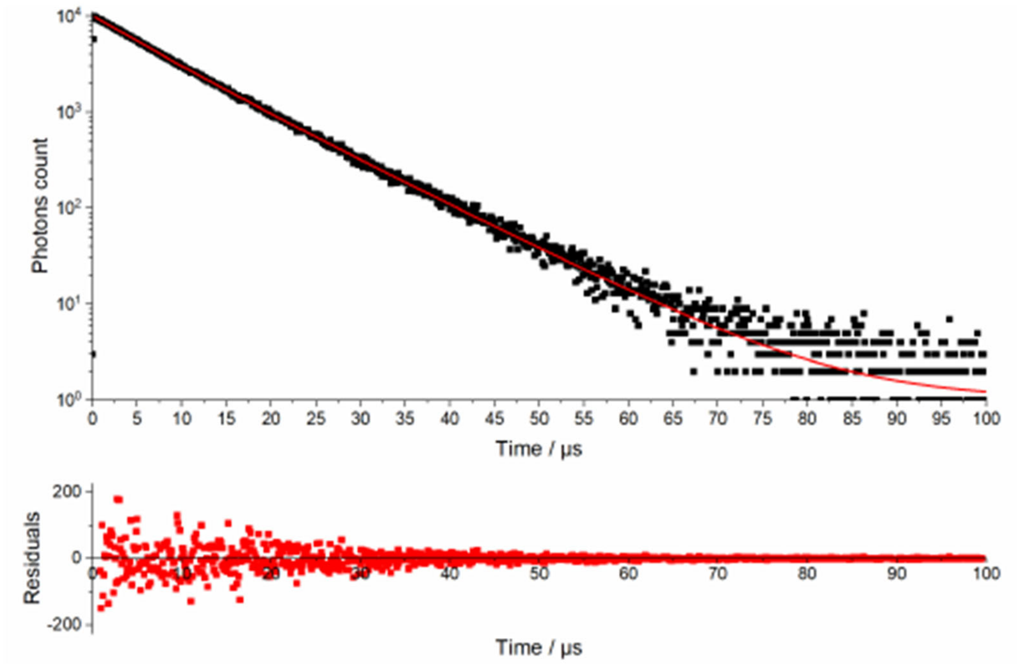

Figure S48. a) Excitation (plain line, λ_{obs} = 760 nm) and emission (dashed line, λ_{ex} = 410 nm) spectra and b) the corresponding emission vs. excitation map of **6** *cis*-[$Re_6Se_8(PEt_3)_4(C{\equiv}CC_6H_5)_2$] in deaerated CH_2Cl_2.

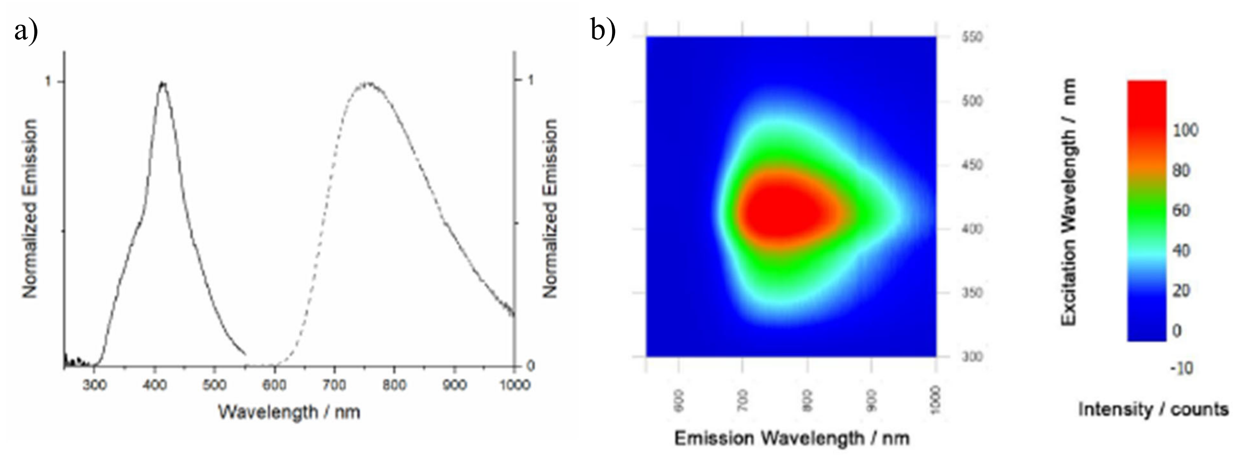

Figure S49. a) Excitation (plain line, λ_{obs} = 760 nm) and emission (dashed line, λ_{ex} = 375 nm) spectra and b) the corresponding emission vs. excitation map of **6** *cis*-[$Re_6Se_8(PEt_3)_4(C{\equiv}CC_6H_5)_2$] in powder phase.

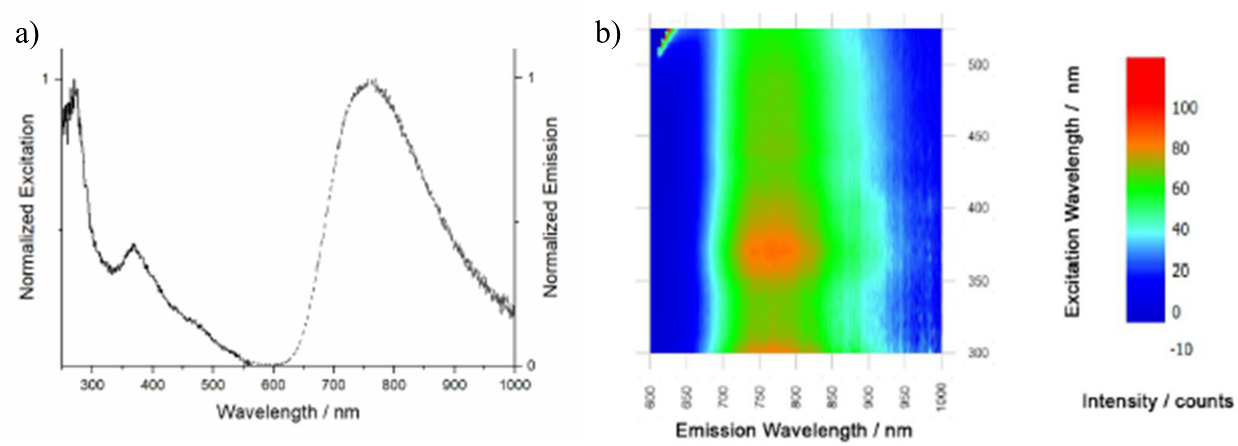

Figure S50. Integrated emission decay profile of **6** *cis*-[$Re_6Se_8(PEt_3)_4(C{\equiv}CC_6H_5)_2$] in a) deaerated CH_2Cl_2 and b) powder phase.

a)

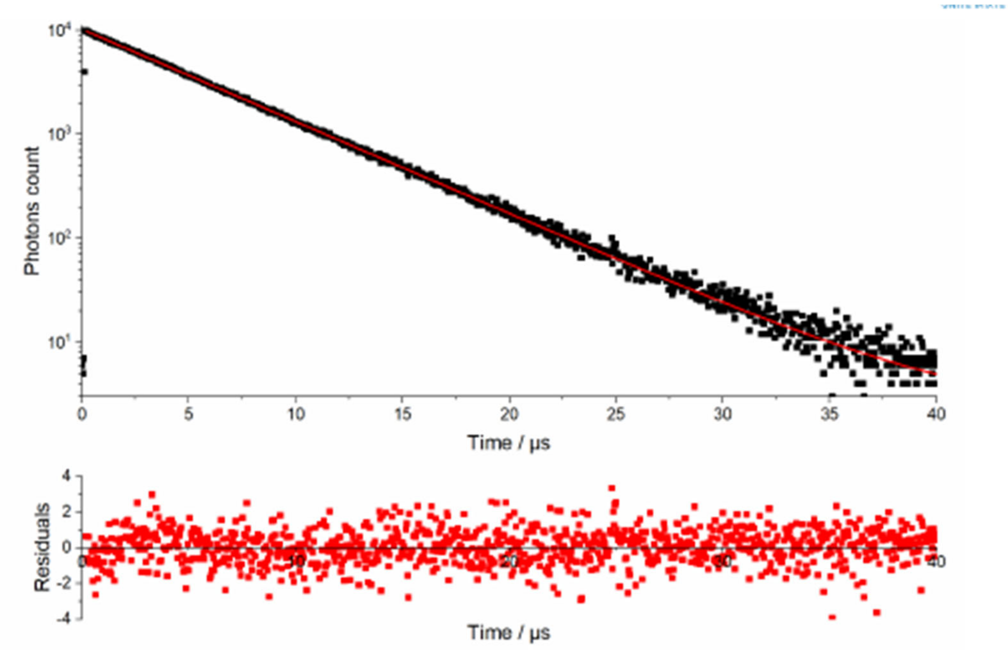

b)

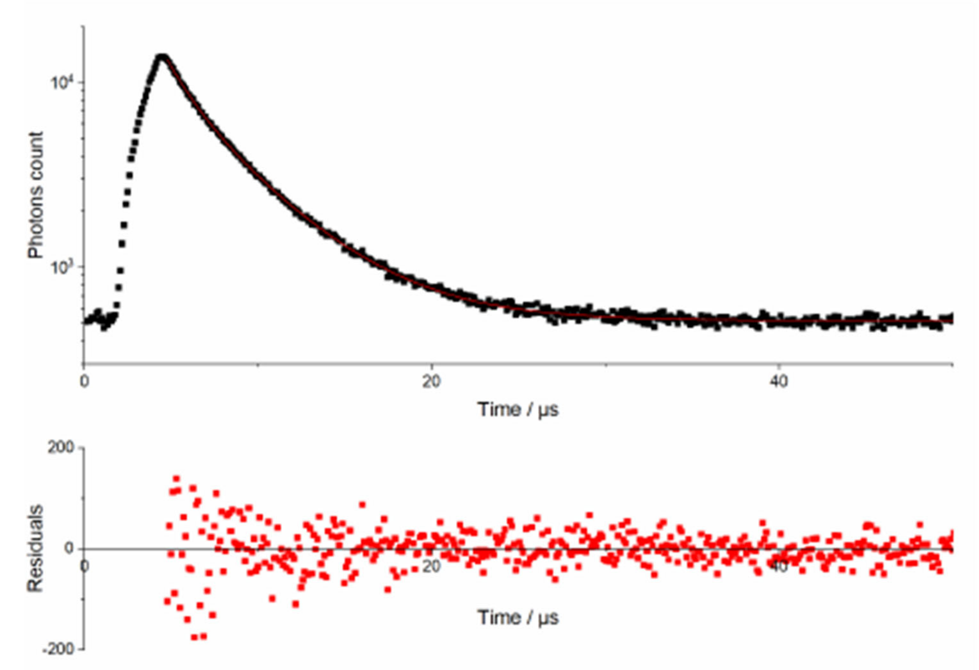

Figure S51. a) Excitation (plain line, λ_{obs} = 750 nm) and emission (dashed line, λ_{ex} = 410 nm) spectra and b) the corresponding emission vs. excitation map of **7** *trans*-[$Re_6Se_8(PEt_3)_4(C{\equiv}CC_6H_5)_2$] in deaerated CH_2Cl_2.

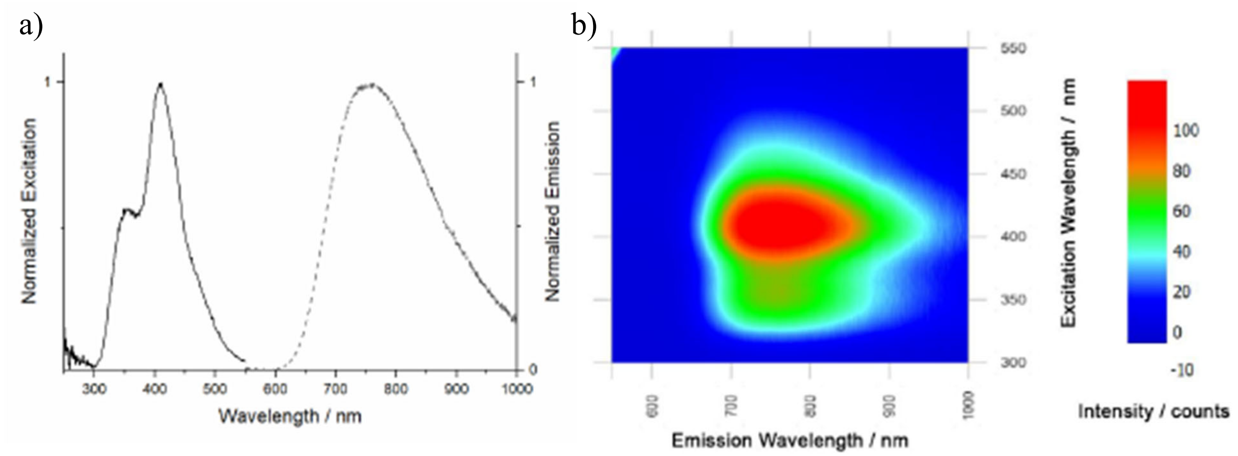

Figure S52. a) Excitation (plain line, λ_{obs} = 790 nm) and emission (dashed line, λ_{ex} = 410 nm) spectra and b) the corresponding emission vs. excitation map of **7** *trans*-[$Re_6Se_8(PEt_3)_4(C{\equiv}CC_6H_5)_2$] in powder phase.

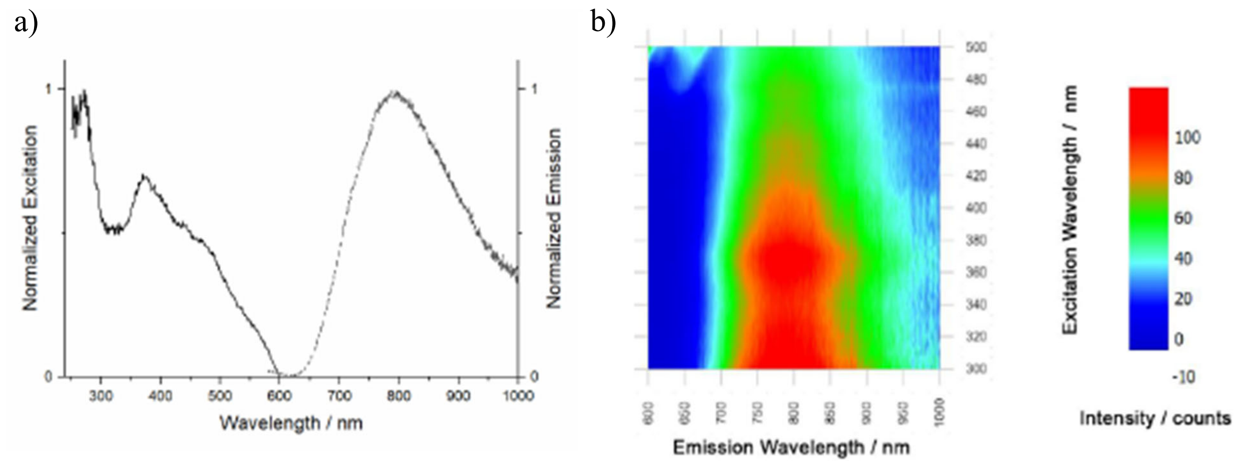

Figure S53. Integrated emission decay profile of **7** *trans*-[$Re_6Se_8(PEt_3)_4(C{\equiv}CC_6H_5)_2$] in a) deaerated CH_2Cl_2 and b) powder phase.

a)

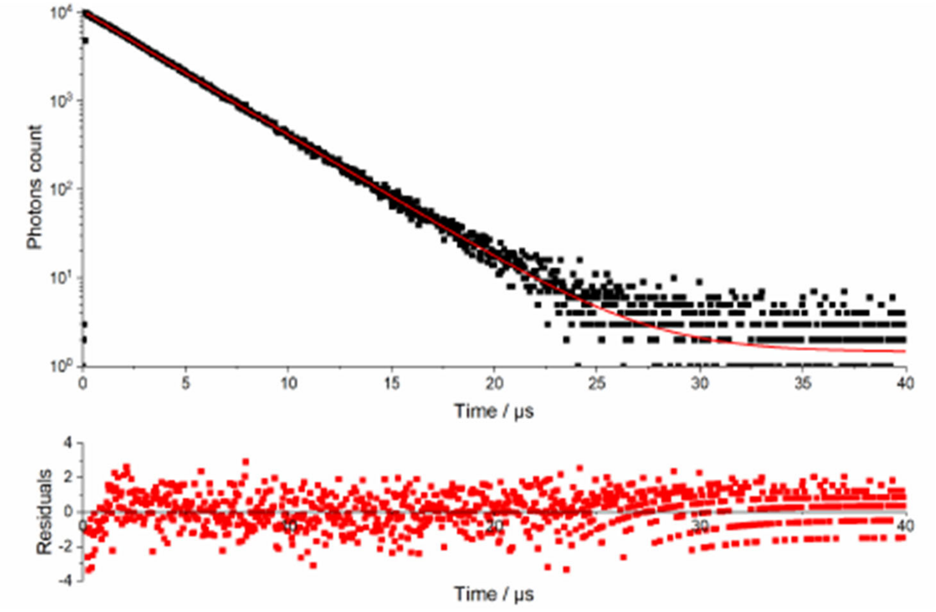

b)

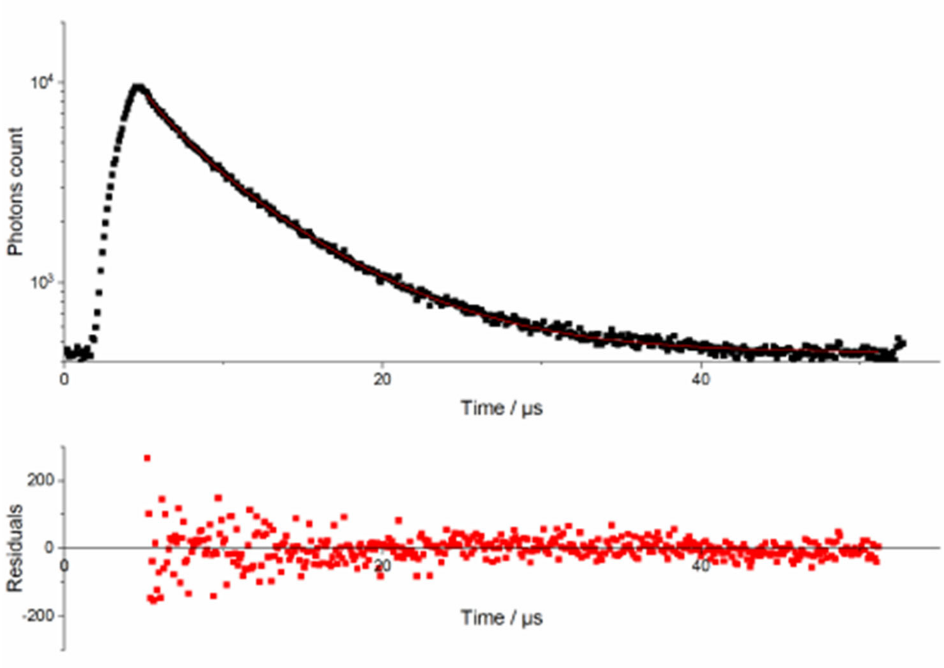

Supplement: Supplementary file 1 [file om5c00245_si_001.pdf]
